# Structure, function and evolution of the bacterial DinG-like proteins

**DOI:** 10.1016/j.csbj.2025.03.023

**Published:** 2025-03-17

**Authors:** Kaiying Cheng

**Affiliations:** a Zhejiang Key Laboratory of Medical Epigenetics, Department of Immunology and Pathogen Biology, School of Basic Medical Sciences, Affiliated Hospital of Hangzhou Normal University, Hangzhou Normal University, Hangzhou 311121, China; b State Key Laboratory for Diagnosis and Treatment of Infectious Diseases, The First Affiliated Hospital, College of Medicine, Zhejiang University, Hangzhou 310003, China

**Keywords:** DinG, Structure, SOS response, DNA repair, CRISPR interference

## Abstract

The damage-inducible G (DinG)-like proteins represent a widespread superfamily 2 (SF2) of DNA helicases, exhibiting remarkable diversity in domain architecture, substrate specificity, regulatory mechanisms, biological functions, interaction partners, and taxonomic distribution. Many characterized DinG-like proteins play critical roles in bacterial stress responses and immunity, including the SOS response, DNA repair, and phage interference. This review aims to provide a summary of bacterial DinG-like proteins, categorizing them into subgroups such as DinG, YoaA, CasDinG, CasDinG-HNH, ExoDinG, pExoDinG, EndoDinG, RadC-like DinG, sDinG, and others. This classification provides an analysis of sequence–structure–function relationships within this superfamily. Further sequence clustering revealed inter-cluster relationships and subgroup heterogeneity, suggesting potential functional divergence. Integrating sequence analysis, domain architecture, structural data, and genomic context enabled functional predictions for these DinG-like protein subgroups, shedding light on their evolutionary and biological significance.

## Graphical Abstract

**Figure:**
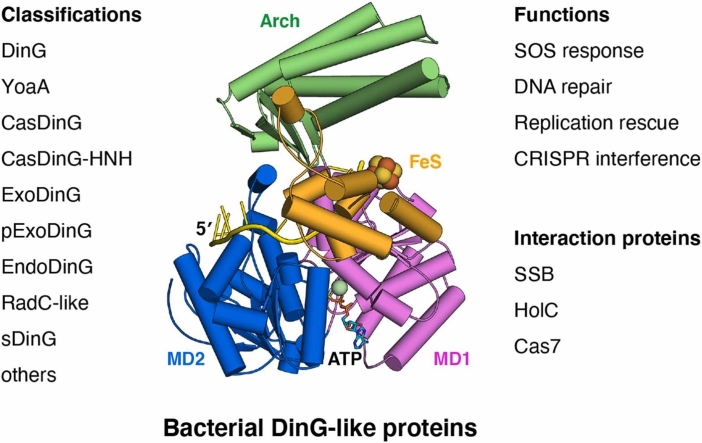

## 1 Introduction

The damage-inducible G (DinG)-like proteins are members of superfamily 2 (SF2) DNA helicases, characterized by their 5′–3′ unidirectional single-stranded DNA (ssDNA) translocation activity [1]. This protein family typically exhibits a four-domain structure comprising two canonical helicase motor domains, helicase motor domain 1 (MD1) and helicase motor domain 2 (MD2), along with two unique domains: the iron–sulfur-cluster-binding (FeS) and Arch domains [2], [3], [4], [5]. The FeS domain generally coordinates an FeS cluster for redox signal sensing, although certain subgroups have evolved a pseudo-FeS (pFeS) domain that lacks this cluster. Both the FeS and Arch domains are inserted within MD1.

*Escherichia coli* DinG (*Ec*DinG), the founding member of the DinG-like family, was the first bacterial DinG protein to be characterized [1]. Its expression is significantly upregulated in response to the SOS system, a conserved bacterial defense mechanism for DNA damage repair [6], [7]. Although *Ec*DinG is known to play a crucial role in DNA repair, its exact function remains incompletely understood [7], [8], [9]. Another DinG-like protein in *E. coli*, YoaA, is also regulated by the SOS response. YoaA physically interacts with HolC, the chi subunit of the DNA polymerase III holoenzyme, suggesting a distinct yet complementary role in DNA repair alongside *Ec*DinG [10], [11], [12], [13]. In some bacteria, DinG-like proteins have lost their FeS clusters and acquired additional N- or C-terminal extensions, enabling them to function within the clustered regularly interspaced short palindromic repeat (CRISPR) system [14], [15], [16]. Additionally, many DinG-like proteins have evolved unique motifs or domains, whose biochemical properties and biological functions remain largely uncharacterized. To date, structural information is available only for *Ec*DinG and a CRISPR-associated DinG-like protein from *Pseudomonas aeruginosa* [14], [15], [17].

This review systematically classifies bacterial DinG-like proteins into distinct subgroups. Protein sequences within each subgroup underwent alignment, and structural features were compared using either reported experimental structures or predicted structures obtained from AlphaFold3 when no structural information was available [18]. For functionally characterized subgroups without structural data, we employed structural modeling using homologous proteins from the corresponding species. For entirely unstudied DinG-like subgroups, proteins from well-established model organisms were selected for structural prediction to facilitate future functional investigations. This selection criterion was designed to facilitate subsequent functional investigations by enabling researchers to capitalize on the established genetic tools, standardized experimental protocols, and accumulated biological knowledge available for these model species. Furthermore, biochemical properties and biological functions were comprehensively analyzed by integrating existing literature with structural predictions derived from this study.

## 2 Distribution and evolution of DinG-like proteins in bacteria

To elucidate the phylogenetic distribution and evolutionary trajectory of DinG-like proteins across bacterial taxa, we performed a systematic genome-wide identification of DinG homologs, followed by multiple sequence alignment and phylogenetic analysis. The protein sequence of *Ec*DinG was used as a query for homology searches against the Kyoto Encyclopedia of Genes and Genomes (KEGG) database (https://www.kegg.jp), a manually curated resource for taxonomy-based genomic analysis [19]. Homologous sequences were identified using an E-value threshold of 1e-5, selecting representative strains by prioritizing the initial strain listed in each bacterial order directory (Table S1). Multiple sequence alignment and phylogenetic tree construction were conducted using Clustal Omega (https://www.ebi.ac.uk/jdispatcher), which employs seeded guide trees and hidden Markov model profile–profile techniques [20]. The resulting phylogenetic tree was visualized and annotated using ChiPlot (https://www.chiplot.online) [21].

**Fig. 1:**
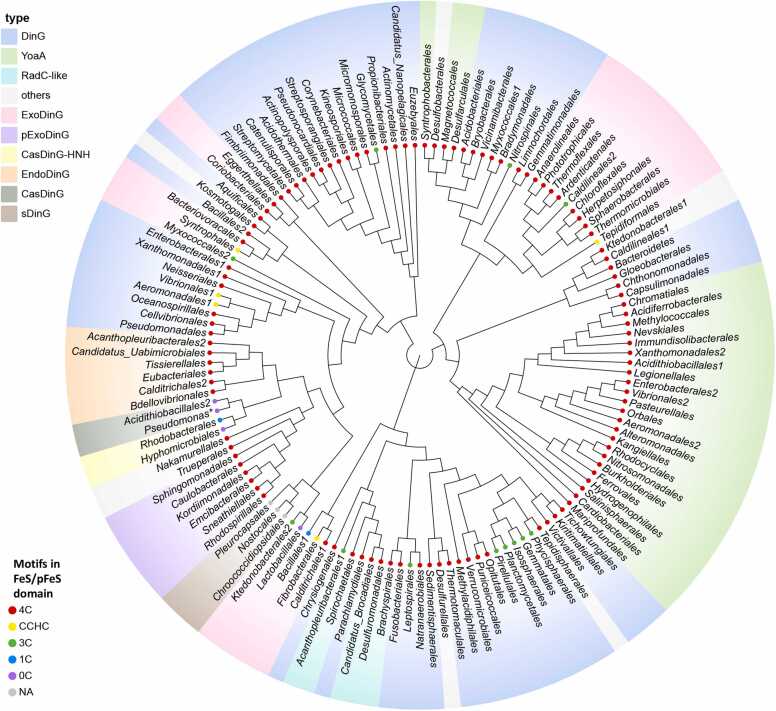
Phylogenetic tree of the DinG-like proteins from different bacteria orders. The DinG-like protein sequences used for constructing the phylogenetic tree were derived from representative bacteria across various bacterial orders. The leaves of the phylogenetic tree are labeled with the names of the bacterial orders, and the corresponding species names and protein sequences can be found in Table S1. Multiple sequence alignment and phylogenetic tree construction was performed using Clustal Omega. The phylogenetic tree was visualized using the ChiPlot online program. Various color blocks indicate the different subgroups of DinG-like proteins. The dots of varying colors denote the presence and number of conserved cysteines in FeS or pFeS, as well as their potential to coordinate with FeS clusters.

Among the 192 bacterial orders analyzed, 125 were found to contain DinG-like homologs (Table S1). Based on domain architecture and predicted function, these DinG-like proteins were classified into ten subgroups: DinG, the originally described DinG; YoaA, a DinG-like protein associated with HolC; CasDinG, a CRISPR-associated DinG; CasDinG-HNH, a CRISPR-associated DinG with a C-terminal HNH domain; ExoDinG, a DinG variant with an N-terminal exonuclease domain; pExoDinG, a DinG with an N-terminal pseudo-exonuclease domain; EndoDinG, a DinG with an N-terminal endonuclease domain; RadC-like DinG, a DinG variant featuring an N-terminal RadC-like domain; sDinG, a short-type DinG; and others, comprising stand-alone homologs that do not align with the defined subgroups (Fig. 1 and Table S1).

DinG-like proteins universally contain the conserved MD1 and MD2 helciase motor domains, along with an Arch domain exhibiting variable lengths and geometries. Most also feature an FeS domain characterized by a CCCC motif, essential for coordinating a [4Fe–4S] cluster. Variants with a single C residue substituted by either H, S, or D residues, such as CCHC or CCCS motifs, have also been identified (Fig. 1 and Table S1). These variants may still retain the ability to coordinate the FeS clusters [22]. In this study, we referred to domains containing four or three C residue, capable of coordinating FeS clusters, as FeS domains. In contrast, domains with only one C residue or none at all, and therefore unable to coordinate FeS clusters, are designated as pFeS domains. The pFeS domains are present in members of the CasDinG and CasDinG-HNH subgroups, as well as certain ExoDinG proteins. Notably, the sDinG subgroup lacks both FeS and pFeS domains entirely.

## 3 Characterizations of DinG-like subgroups

### 3.1 DinG

**Fig. 2:**
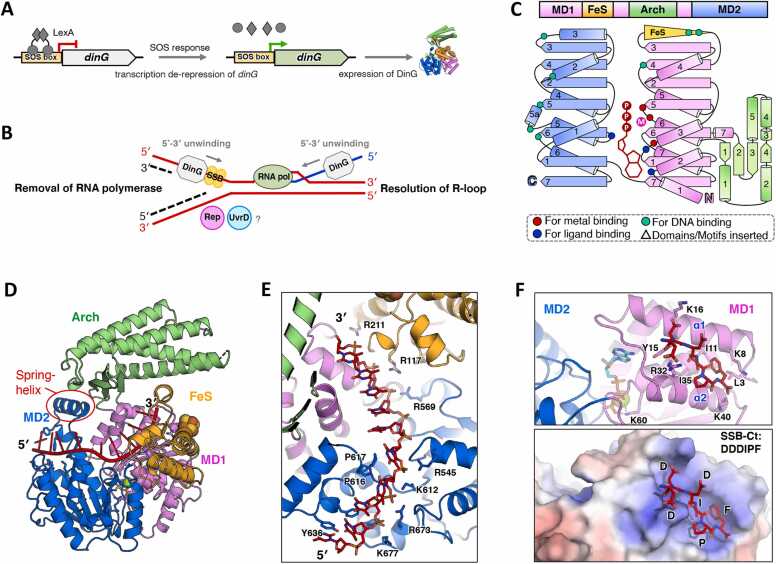
Summary of the biological function and structure of DinG. (A) The cartoon model illustrates the induction of DinG by the SOS response. During the SOS response, there is a de-repression of *dinG* transcription and an increase in DinG protein expression, which is controlled by the LexA repressor. (B) The cartoon model depicts how DinG participates in rescuing transcription-blocked replication forks. In highly transcribed regions, DinG assists in resolving R-loops and removing RNA polymerase, likely with the assistance of Rep and UvrD proteins, to ensure efficient replication. (C) The domain arrangement and the topology diagram of *Ec*DinG. *Ec*DinG was selected for structural analysis within DinG subgroup proteins. The MD1, MD2, Arch, and FeS domains were colored violet, marine, lime green, and orange, respectively. Key residues involved in metal ion coordination, ATP binding, and substrate interaction were highlighted as red dots, blue dots, and cyan dots, respectively. (D) The overall structure of the *Ec*DinG–ssDNA–ADPBeF_3_ complex. The structure (PDB ID: 6FWS) was shown in cartoon form, with each domain colored as described in (C). ssDNA and ADPBeF_3_·Mg^2^^+^ were colored red and green, respectively. (E) A zoomed-in view of the DNA binding mode of *Ec*DinG. The DNA and the key residues involved in DNA interaction were shown as sticks and labelled. (F) AlphaFold 3 predicted *Ec*DinG–SSB-Ct interaction. Upper, zoomed-in view of the predicted *Ec*DinG–SSB-Ct interaction interface. The predicted binding pocket (α1 and α2 of the *Ec*DinG MD1 domain) was illustrated as cartoon. SSB-Ct peptide (DDDIPF) and the potential residues essential for SSB-Ct interaction were depicted as sticks. The input information used for model prediction is detailed in the supplementary material. Bottom, an analysis of the electrostatic properties of the SSB-Ct binding pocket on *Ec*DinG. The electrostatic potential of the SSB interaction interface on the MD1 of *Ec*DinG was determined using APBS, which was then projected onto the solvent-accessible surface of the structure at contouring levels of ± 5 kT (depicted in blue/red). Amino acids of SSB-Ct were represented as sticks, colored red, and labelled.

The *dinG* gene, an acronym for *damage-inducible* G, was initially identified through genetic screening, which measured the transcriptional activation of galactokinase gene fusions in *E. coli* cells treated with mitomycin C (MMC) [7]. Its expression is upregulated in response to DNA damage induced by nalidixic acid in *E. coli* [8]. Subsequently, *dinG* was recognized as a LexA-regulated SOS regulon gene in both *E. coli* and *P. aeruginosa*, with LexA functioning as a repressor of the *dinG* promoter. During the bacterial SOS response, LexA undergoes self-degradation, thereby de-repressing *dinG* expression (Fig. 2A) [6], [7], [23]*.*

The deletion of *dinG* in *E. coli* does not lead to severe phenotypes, suggesting that it is nonessential for cell viability. Although *dinG* expression is induced by ultraviolet (UV) irradiation, mutants lacking this gene displayed only minor sensitivity to UV light and hydrogen peroxide (H_2_O_2_) [24]. Additionally, DinG contributes minimally to the *E. coli* tolerance against azidothymidine (AZT), a chain-terminating nucleoside and DNA replication inhibitor [25]. Functionally, DinG has been implicated in resolving R-loops and displacing RNA polymerase, thereby facilitating replication progression in highly transcribed genomic regions, potentially in cooperation with other DNA helicases such as Rep and UvrD (Fig. 2B) [26]. In *Neisseria meningitidis*, *dinG* deletion does not impair growth or transformation and does not affect sensitivity to oxidative stress (e.g., H_2_O_2_ and paraquat) or alkylating stress (e.g., methyl methanesulfonate, MMS) compared to the wild-type strain [27]. However, exposure to MMC and bleomycin results in significant survival differences between the wild-type and mutant strains, suggesting that DinG plays a role in specific DNA damage repair pathways in *N. meningitidis* [27]. Transcriptome analyses further indicate that *N. meningitidis* DinG (*Nm*DinG) may be involved in the regulation of metabolic pathways [27].

The biochemical properties of DinG have been extensively investigated in *E. coli* [24], [28], *Mycobacterium tuberculosis* [29], and *N. meningitidis* [27]. These investigations demonstrate that DinG can unwind various DNA structures, including overhangs, flap structures, forks, D-loops, R-loops, and G-quadruplex (G4) DNA in a 5′–3′ direction [9], [24], [27], [29], [30]. Additionally, *M. tuberculosis* DinG exhibits helicase activity on three-way junctions and Holliday junctions [29]. The single-stranded DNA-binding protein (SSB) from *E. coli* and *N. meningitidis* interacts with DinG, forming stable protein complexes that enhance its helicase activity (Fig. 2B) [27], [31].

Genomic analyses indicated that approximately 30 % of bacterial genomes encode DinG homologs (Fig. 1 and Table S1). However, detailed structural characterization has been conducted only for *Ec*DinG [17]. The structures of *Ec*DinG bound to ssDNA, in the absence and presence of a non-hydrolyzable ATP analog (PDB ID: 6FWR and 6FWS), have been determined using X-ray crystallography at 2.5 Å resolution [17].

*Ec*DinG consists of two helicase motor domains (MD1 and MD2), an Arch domain, and an FeS domain [17]. Each helicase motor domain comprises seven β-sheets and multiple α-helices (MD1 contains eight α-helices, while MD2 contains seven) (Figs. 2C, D). The Arch domain, inserted between α6 and α7 of MD1, consists of four β-sheets and five α-helices. The FeS domain, located between β3 and β4 of MD1, features multiple α-helices and a CCCC motif that coordinates a [4Fe–4S] cluster (Figs. 2C, D). *Ec*DinG interacts with ssDNA via a groove spanning the surfaces of MD1 and MD2 [17]. The ssDNA bases remain stacked until they encounter a proline-rich strand (P motif, situated between β5 and α5 of MD2). An aromatic residue (Y636 in *Ec*DinG, located before α5 of MD2) intercalates between the first and second bases at the 5′ end of the ssDNA on MD2, forming a stacking interaction (Fig. 2E). Two conserved R residues (R117 and R211 in *Ec*DinG) wihtin the FeS domain directly interact with ssDNA and contribute to DNA translocation (Fig. 2E) [17]. Electrochemical studies demonstrate that the [4Fe–4S] cluster of DNA-bound EcDinG remains redox-active under cellular conditions, with ATP hydrolysis enhancing DNA-mediated redox signaling [32]. Disruption of the FeS domain severely impairs *Ec*DinG helicase activity [17], [28].

Structural comparisons between the *Ec*DinG–ssDNA binary complex (PDB ID: 6FWR) and the *Ec*DinG–ssDNA–ADPBeF_3_ ternary complex (PDB ID: 6FWS) reveal an “inchworm” mechanism for 5′–3′ ssDNA translocation [17]. ATP binding and hydrolysis mediate the relative position changes in MD1 and MD2, while the Arch domain’s movement is linked to MD2 via a spring-helix (α5a of MD2), further facilitating translocation and unwinding. The conservation of key residues and structural elements involved in DNA binding and translocation across the DinG subgroup suggests an evolutionarily conserved mechanism for these processes (Fig. S1).

SSB, a highly conserved and essential protein, binds cellular ssDNA to protect it from degradation and prevent secondary structure formation. Additionally, SSB coordinates the activities of various genome maintenance proteins, playing essential roles in replication, transcription, and DNA repair [33]. The C-terminal tail of SSB (SSB-Ct), which mediates numerous protein–protein interactions [33], appears to be critical for DinG binding. Truncation of *N. meningitidis* SSB-Ct (*Nm*SSB-Ct) reduces its affinity for DinG [27]. The F177C mutation in *E. coli* SSB-Ct completely abolishes protein–protein interaction, thereby inhibiting *Ec*DinG helicase activity [31]. Residue K72 in *Nm*DinG has been identified as crucial for its interaction with SSB [27]. However, structural data from the *Ec*DinG–ssDNA–ADPBeF_3_ ternary complex (PDB ID: 6FWS) indicate that K60 in *Ec*DinG, corresponding to the conserved residue K72 in *Nm*DinG, participates in ATP binding (Fig. 2D and Fig. S1) [17]. AlphaFold3 structural predictions suggest that the region most likely to interact with SSB-Ct is located within an alkaline pocket formed by α1 and α2 of MD1, which is spatially separated from K60 (Fig. 2F and Fig. S1). The key residues forming this alkaline pocket in *Ec*DinG (L3, K8, I11, Y15, K16, R32, I35 and K40) are not conserved among other DinG homologs (Fig. 2F and Fig. S1). Consequently, whether other DinG homologs interact with SSB in a similar manner, and whether this interaction interface is evolutionarily conserved, remains unclear.

### 3.2 YoaA

**Fig. 3:**
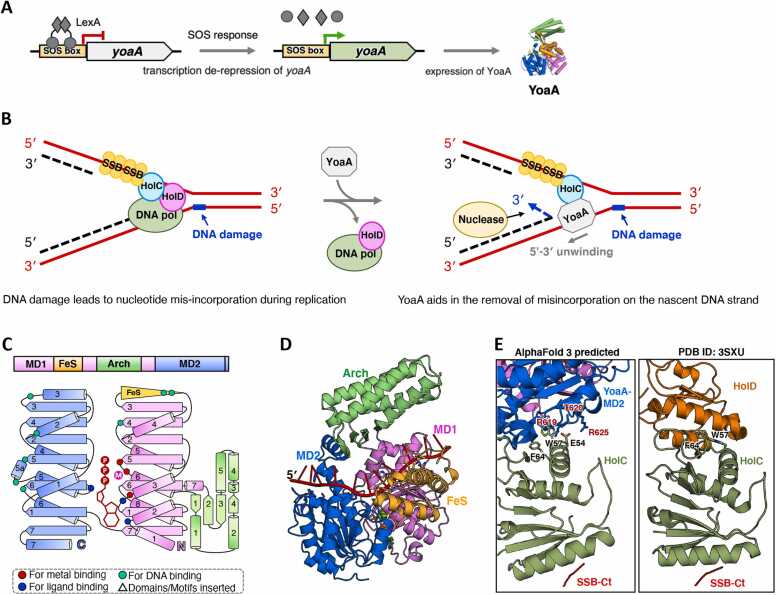
Summary of the biological function and predicted structure of YoaA. (A) The cartoon model illustrates the induction of YoaA. During the SOS response, there is a de-repression of *yoaA* transcription and an increase in DinG protein expression, which is regulated by the LexA repressor. (B) The cartoon model depicts how YoaA assists in genomic stability. During DNA replication, DNA damage leads to nucleotide mis-incorporation. HolC–SSB recruits YoaA helicase, which unwinds the 3′ nascent strand, allowing nuclease access for the removal of the mis-incorporated nucleotides. (C) The domain arrangement and the topology diagram of *Ec*YoaA. *Ec*YoaA was selected for structural analysis within the YoaA subgroup proteins. The MD1, MD2, Arch, and FeS domains were colored violet, marine, lime green, and orange, respectively. Key residues involved in metal ion coordination, ATP binding, and substrate interaction were highlighted as red dots, blue dots, and cyan dots, respectively. (D) The overall structure of *Ec*YoaA–ssDNA–ATP complex. The AlphaFold3 predicted structure was shown in cartoon form, with each domain colored as described in (C). ssDNA and ATP·Mg^2+^ were colored red and green, respectively. The input information used for model prediction is detailed in the supplementary material. (E) Structural comparison between *Ec*YoaA–HolC–SSB-Ct complex and *Ec*HolD–HolC–SSB-Ct complex. A zoomed-in view of the protein-protein interfaces in the AlphaFold3 predicted *Ec*YoaA–HolC–SSB-Ct complex structure and the experimentally determined *Ec*HolD–HolC–SSB-Ct complex structure (PDB ID: 3XSU) is shown. The (predicted) key residues involved in protein-protein interactions were shown as sticks and labelled. The input information used for model prediction is detailed in the supplementary material.

Another DinG-like protein, YoaA, similar as DinG, is induced by DNA damage as part of the LexA-regulated SOS response in *E. coli* (Fig. 3A) [10], [11]. Currently, no structural information is available for YoaA. Sequence alignment indicated that *E. coli* YoaA (*Ec*YoaA) shares a similar four-domain structure with *Ec*DinG, with the exception that *Ec*YoaA contains an additional conserved α-helix (α7) at the C-terminus of its MD2 domain (Fig. S2). *Ec*YoaA directly interacts with HolC, the chi subunit of DNA polymerase III, with α7 predicted to serve as a crucial interaction interface (Fig. 3B, C) [12], [25]. Based on genome co-occurrence analysis and sequence alignment, this study establishes classification criteria to distinguish the YoaA-type subgroup from the DinG subgroup: (1) the presence of the α7 helix in the MD2 domain and (2) the co-occurrence of a HolC homolog within the same genome.

According to the established classification criteria, YoaA homologs were identified in 26 bacterial orders (Fig. 1 and Table S1). Notably, bacteria from Enterobacterales, Xanthomonadales, Vibrionales, and Aeromonadales harbor both DinG and YoaA (Fig. 1 and Table S1), indicating distinct functional roles of these two proteins *in vivo*. Experimental evidence indicates that *yoaA* contributes to cellular tolerance against replication inhibitor AZT, while deletion of *dinG* only resulted in slight AZT sensitivity [12], [25], [34].

*Ec*YoaA has been confirmed to facilitate access to the 3′ nascent strand during replication and reduce T-to-A transversion mutations in *E. coli,* likely through recruitment by HolC at the replication fork [11]. Under normal physiological conditions, HolC forms a complex with HolD (the psi subunit of DNA polymerase III) and SSB, assisting in DNA replication [35]. Further investigation revealed that YoaA, HolC, and SSB can assemble into a multi-protein complex, while the binding of HolC to YoaA and HolD appears to be mutually exclusive [12]. Overexpression of full-length YoaA inhibits cell growth, potentially by displacing HolD from HolC, disrupting DNA polymerase III function, and impairing replication [11]. This led to a model proposing that YoaA contributes to genomic stability by responding to DNA damage-induced base misincorporation. In this model, HolC–SSB recruits YoaA to the replication fork, where YoaA unwinds the 3′ nascent strand, facilitating the removal of misincorporated nucleotides by nucleases (Fig. 3B) [11], [12].

Attempts to express YoaA alone in *E. coli* BL21(DE3) cells resulted in poor solubility, complicating purification and biochemical characterization [13]. However, co-expression with HolC improved its solubility [13]. The co-purified YoaA–HolC complex demonstrated DNA-dependent ATPase and helicase activities, preferentially unwinding forked duplex DNA with both 3′ and 5′ single-stranded overhangs rather than duplex DNA with only a 5′ overhang [13]. Additionally, the YoaA–HolC complex exhibited the capability to unwind damaged DNA containing abasic sites or 3′-end lesions that obstruct replication extension [13].

Sequence alignment analysis and the AlphaFold3-predicted structure of the *Ec*YoaA–ssDNA–ATP complex revealed that key residues in the ATPase site, the P motif, and DNA-binding regions in MD1 and MD2, which are critical for DinG function, are also conserved among YoaA homologs (Fig. 3C, D and Fig. S2). This suggests that YoaA shares similar DNA unwinding and translocation mechanisms with DinG. Mutations in critical residues within MD1, MD2, or the FeS domains abolished YoaA's ability to confer AZT tolerance, underscoring their importance in its biological function [12].

The AlphaFold3-predicted structure of the *Ec*YoaA–HolC–SSB-Ct complex exhibits interaction interfaces that are remarkably similar to those of the *Ec*HolD–HolC–SSB-Ct complex (PDB ID: 3XSU) (Fig. 3E) [35]. Mutagenesis studies identified R619 and T620 in YoaA’s α7 helix of MD2 as critical for HolC interaction (Fig. 3E) [11]. Additionally, two HolC residues, F64 and W57, essential for its interaction with HolD, were also found to be crucial for YoaA binding (Fig. 3E) [12]. These findings suggest that YoaA and HolC form a distinct repair-associated complex separate from the DNA polymerase III holoenzyme. HolC thus appears to establish two functional complexes: HolC–HolD for replication and HolC–YoaA for repair, both recruited to ssDNA via HolC–SSB interactions (Fig. 3B, E) [12].

### 3.3 CasDinG

Type IV-A CRISPR-Cas systems, which facilitate antiviral defense, consistently incorporate a DinG-like protein subgroup designated as Csf4 [36]. These proteins were later reclassified as CRISPR-associated DinG (CasDinG) due to their sequence similarity with DinG helicases and functional integration within CRISPR-Cas pathways [37]. Phylogenetic analyses reveal that CasDinG sequences form a distinct cluster separate from other DinG-like proteins, even within the same host organism (Fig. 1), suggesting functional specialization.

**Fig. 4:**
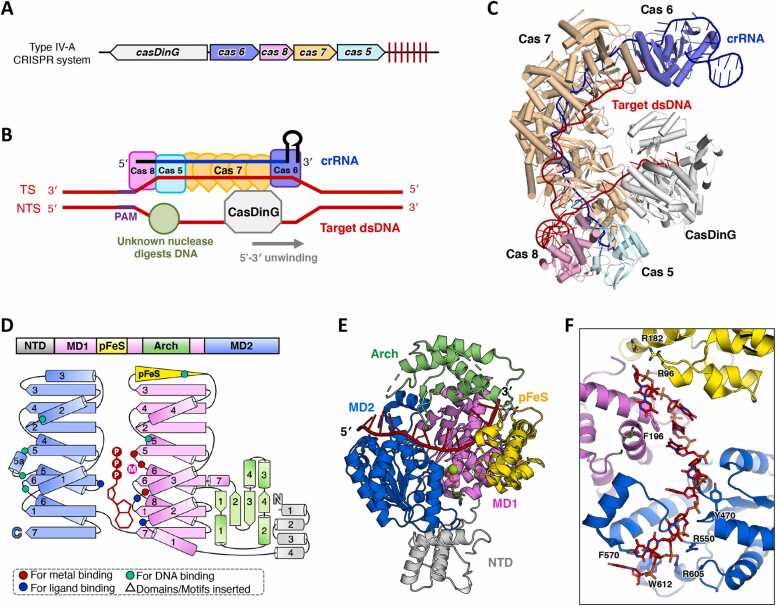
Summary of the biological function and structure of CasDinG. (A) The *casDinG* related gene operon with a CRISPR array in Type IV-A CRISPR system. (B) A simplified cartoon model illustrating the involvement of CasDinG in interference within a Type IV-A CRISPR system. (C) The overall CryoEM structure of *Pa*CasDinG bound to the Csf–crRNA–dsDNA complex (PDB ID:7XG3). The Cas5, Cas6, Cas7, Cas8 and CasDinG subunit were colored cyan, slate, wheat, pink, and white, respectively. Target dsDNA and crRNA were colored red and blue, respectively. (D) The domain arrangement and the topology diagram of *Pa*CasDinG. *Pa*CasDinG was selected for structural analysis within the CasDinG subgroup proteins. The MD1, MD2, Arch, pFeS, and NTD domains were colored violet, marine, lime green, yellow, and white, respectively. Key residues involved in metal ion coordination, ATP binding, and substrate interaction were highlighted as red dots, blue dots, and cyan dots, respectively. (E) The overall structure of *Pa*CasDinG–ssDNA–ATP complex. The NTD truncated complex structure (PDB ID: 7XF1) and the AlphaFold3 predicted NTD were combined and shown in cartoon form, with each domain colored as described in (D). ssDNA and ATP·Mg^2+^ were colored red and green, respectively. (F) A zoomed-in view of the DNA binding mode of *Pa*CasDinG. The DNA and the key residues involved in DNA interaction were shown as sticks and labelled.

Biochemical analyses of *P. aeruginosa* CasDinG (*Pa*CasDinG) have demonstrated its role as an ATP-dependent 5′–3′ DNA translocase with the ability to unwind double-stranded DNA and RNA/DNA hybrids [14], [15]. The gene encoding *Pa*CasDinG is plasmid-encoded and positioned in the opposite orientation upstream of an operon containing *cas6*, *cas8*, *cas7*, *cas5*, and the CRISPR array (Fig. 4A). The Cas6, Cas8, Cas7, and Cas5 proteins assemble into a Csf complex that binds CRISPR-derived RNA (crRNA), forming an eight-subunit ribonucleoprotein complex consisting of one Cas5, one Cas6, five Cas7, and one Cas8 (Fig. 4B) [14]. The CryoEM structure of the *Pa*CasDinG–Csf–crRNA–dsDNA complex, which includes a nicked non-target strand (NTS) with a 3′ overhang (PDB ID:7XG3; 3 Å), demonstrates that CasDinG interacts directly with the Cas7 forefinger motifs via multiple binding interfaces located on MD1, MD2, and the Arch domain (Fig. 4C)[14]. A proposed model suggests that CasDinG is recruited to the dsDNA-bound Csf complex through interactions with both the NTS and the Cas7 forefinger motifs. CasDinG is hypothesized to slide along the NTS in a 5′–3′ direction, unwinding the target dsDNA, which may subsequently be degraded by an unknown cellular nuclease (Fig. 4B) [14].

*Pa*CasDinG is the only CasDinG protein with an experimentally resolved structure, revealing five domains: MD1, MD2, Arch, pFeS, and an N-terminal domain (NTD) (Fig. 4C) [14], [15]. The *Pa*CasDinG–ssDNA complex structure (PDB ID: 7XF1), determined by X-ray crystallography at 3.2 Å resolution, demonstrates that MD1 and MD2 are both involved in ssDNA binding (Fig. 4D, E) [14]. Key DNA-binding and ATPase residues are highly conserved on MD2 and MD1 (Fig. 4C and Fig. S3). A mutation in the ATP-binding site (K136A) abolished CRISPR interference activity [38], [39].

Although CasDinG lacks the conserved C residues required for iron–sulfur cluster coordination, it retains a pFeS domain of equivalent length to the FeS domain in DinG (Fig. 4D, E). Structural analysis of the *Pa*CasDinG–ssDNA complex suggests that the pFeS domain plays a structural role and contributes to DNA binding (Fig. 4E, F) [14]. The Arch domain closely resembles that of DinG and YoaA, though with a more compact arrangement of secondary structure elements. Genetic studies confirm that both the pFeS and Arch domains are essential for type IV-A immunity [15]. The NTD of PaCasDinG, which remains unresolved in experimental structural data, was predicted in this study using AlphaFold3 and is composed of four α-helices (Fig. 4D and Fig. S3). Jackson et al. revealed that the NTD of CasDinG is susceptible to proteolysis, and its deletion impairs type IV-A immunity without affecting ATPase, ssDNA binding, or helicase activities [15], [40]. They further hypothesized that the NTD interacts with dsDNA based on its structural similarity to known dsDNA-binding proteins [15].

### 3.4 CasDinG-HNH

Altae-Tran et al. recently identified a unique variant of the CasDinG protein from *Sulfitobacter sp.* JL08 (Rhodobacterales), which is associated with a variant type IV CRISPR-Cas system [16]. This DinG-like subgroup was named CasDinG-HNH in this paper, given its association with the CRISPR-Cas system and the presence of an HNH nuclease domain at its C-terminus. A homolog of CasDinG-HNH was also identified within the chromosomal repertoire of *Nordella sp.* HKS 07 (Hyphomicrobiales), expanding the known distribution of this subgroup (Fig. 1 and Table S1).

**Fig. 5:**
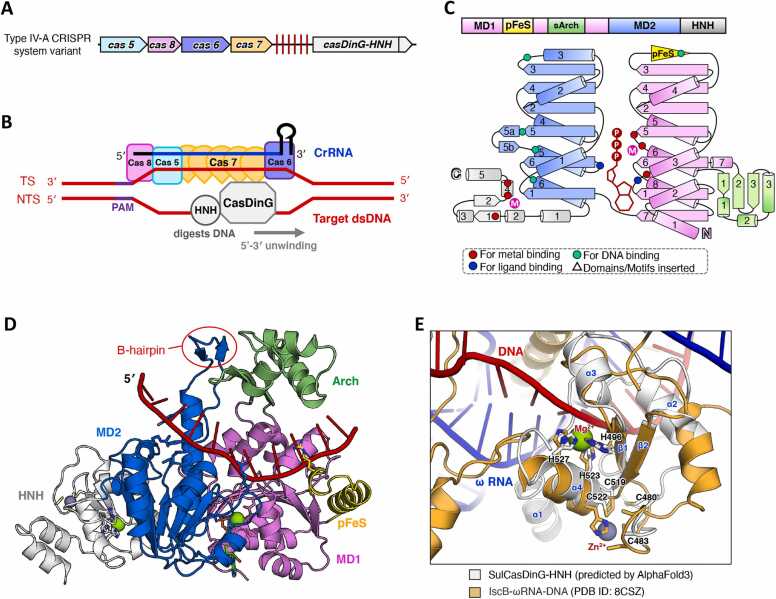
Summary of the biological function and predicted structure of CasDinG-HNH. (A) The *casDinG-HNH* related gene operon with a CRISPR array in a Type IV-A CRISPR system variant. (B) A simplified cartoon model illustrating the involvement of CasDinG-HNH in interference within a Type IV-A CRISPR system variant. (C) The domain arrangement and the topology diagram of CasDinG-HNH. CasDinG-HNH from *Sulfitobacter sp.* JL08 was selected for structural analysis within the CasDinG-HNH subgroup proteins. The MD1, MD2, Arch, pFeS, and HNH domains were colored violet, marine, lime green, yellow, and white, respectively. Key residues involved in metal ion coordination, ATP binding, and substrate interaction were highlighted as red dots, blue dots, and cyan dots, respectively. (D) The overall structure of AlphaFold3 predicted *Su*CasDinG-HNH–ssDNA–ATP complex. The structure was shown in cartoon form, with each domain colored as described in (C). ssDNA and ATP·Mg^2+^, Zn^2+^ were colored red, green, and purple blue respectively. The input information used for model prediction is detailed in the supplementary material, while the DNA/RNA hybrid duplex that could not be modeled with high-quality has been omitted from this figure for clarity. (E) Structural comparison of the HNH domain between *Su*CasDinG-HNH and IscB–ωRNA–DNA. A zoomed-in view of the catalytic sites within the HNH, as predicted by AlphaFold3 for *Su*CasDinG-HNH, alongside the crystallographic structure of IscB in complex with ωRNA and DNA (PDB ID: 8CSZ). Key residues implicated in metal catalysis and Zn-finger coordination in *Su*CasDinG-HNH are depicted as sticks and labeled. ωRNA and DNA were shown as cartoon, colored blue and red, respectively.

The gene encoding CasDinG-HNH is typically located downstream of *cas5*, *cas8*, *cas6*, *cas7*, and the CRISPR array, forming a variant type IV system (Fig. 5A). This system exhibits interference activity by RNA-guided protospacer-adjacent motif (PAM)–dependent directional dsDNA degradation [16]. Mutations in the conserved residues of *Sulfitobacter sp.* JL08 CasDinG-HNH (*Su*CasDinG-HNH), crucial for divalent cation coordination at the ATPase sites (D139, E140) and nuclease sites (H497, H523), eliminated this interference activity [16]. A proposed mechanism suggests that CasDinG-HNH binds to the NTS within the R-loop and translocates in the 5′–3′ direction along the NTS while simultaneously cleaving both the target strand and non-target strands (Fig. 5B) [16].

Currently, no experimentally determined structural information is available for CasDinG-HNH. However, AlphaFold3 predictions of the *Su*CasDinG-HNH–ssDNA–ATP complex revealed notable structural deviations from the previously characterized *Ec*DinG and *Pa*CasDinG (Fig. 5C, D). The Arch domain in *Su*CasDinG-HNH is more compact, consisting of three α-helices and three β-sheets, while the pFeS domain is highly condensed, comprising only two α-helices (Fig. 5D and Fig. S4). In *Ec*DinG (PDB ID: 6FWS), the α5a helix in MD2 functions as a spring-helix, facilitating Arch domain movement and playing a crucial role in DNA translocation and unwinding efficiency (Fig. 2D) [17]. This spring-helix is conserved across all DinG-like subgroups examined in this study, with the exception of *Su*CasDinG-HNH, which lacks α5a but features a distinctive β-hairpin structure (comprising β5a and β5b) instead (Fig. 5D). Whether this β-hairpin serves a functional role equivalent to the spring-helix remains an open question. Additionally, while most DinG-like proteins contain a β-sheet (β7) following α6 in MD2, *Su*CasDinG-HNH uniquely replaces β7 with an extended HNH nuclease domain.

The HNH nuclease features a ββα-metal fold with a central divalent metal ion. It typically functions as monomers, creating single nicks in nucleic acids to degrade foreign or host genomes, or as homodimers, introducing double-stranded DNA breaks for DNA restriction, integration, recombination, and repair [41]. The canonical HNH motif consists of a strictly conserved H residue at the end of the first β-strand (corresponding to the first H in the HNH motif), a highly conserved H residue within the α-helix (corresponding to the last H), and a D residue in the loop between the two β-strands, which stabilizes the ββα-metal fold [41].

Diversity analysis interface (DALI) [42] analysis revealed that the HNH domain of *Su*CasDinG-HNH exhibits the highest structural similarity with the HNH domains of IscB (PDB ID: 8CSZ) [43], *E. coli* McrA (EcoKMcrA) (PDB ID: 6GHC)[44], and Cas5e from *Candidatus Cloacimonetes* bacterium ADurb.Bin088 (PDB ID: 8YB6)[45] (Table S2). These proteins are involved in host defense mechanisms, targeting foreign nucleic acids through both site-specific and non-site-specific cleavage, suggesting a similar functional role for *Su*CasDinG-HNH.

Although AlphaFold 3 failed to model a nucleic acid substrate within the HNH domain of *Su*CasDinG-HNH (Fig. S4), its predicted structure closely aligns with the experimentally determined HNH domain of IscB, which is complexed with ωRNA and dsDNA (PDB ID: 8CSZ) (Fig. 5E). Both the HNH domains contain the two conserved H residues (H496 and H523 in *Su*CasDinG-HNH) within the ββα-metal fold but lack the N residue. Additionally, both domains feature an extra H residue (H527 in *Su*CasDinG-HNH) immediately following the last H residue in the ββα-metal fold, which may also participate in metal ion coordination (Fig. 5E). HNH domains generally engage DNA via the minor groove, anchoring to the phosphate backbone through their α-helix and first β-strand, a binding mode clearly demonstrated in the IscB–ωRNA–DNA complex [41]. This binding mode is clearly illustrated in the structure of the IscB–ωRNA–DNA complex, offering a model for understanding how *Su*CasDinG-HNH interacts with its substrate (Fig. 5E). Furthermore, similar as IscB, *Su*CasDinG-HNH contains a Zn knuckle motif formed by four C residues located in loops preceding the first β-strand or following the last β-strand of the ββα-metal fold (Fig. 5E). Zn knuckles are commonly found in HNH nucleases and are thought to contribute to structural integrity [46].

In contrast, the putative HNH domain in *Nordella* sp. HKS 07 CasDinG-HNH (*No*CasDinG-HNH) displays distinct features compared to that of *Su*CasDinG-HNH (Fig. S4). First, the *No*CasDinG-HNH HNH domain utilizes two H residues and a D resdiue for metal ion coordination, rather than the two H residues found in *Su*CasDinG-HNH. Second, it lacks the conserved Zn knuckle motif. Additionally, *No*CasDinG-HNH possesses a longer pFeS domain (approximately 60 additional amino acids) than *Su*CasDinG-HNH (Fig. S4). Notably, the gene encoding *No*CasDinG-HNH is flanked upstream only by the *cas6* gene and a CRISPR array, while lacking *cas5*, *cas8*, and *cas7* genes. While *No*CasDinG-HNH is phylogenetically closely related to *Su*CasDinG-HNH (Fig. 1), these structural and genomic differences raise intriguing questions regarding its functional role and whether it operates through a similar mechanism *in vivo*.

### 3.5 ExoDinG

DinG-like proteins in several bacterial orders have evolved to acquire a fused putative 3′–5′ exonuclease domain at their N-terminus, and are therefore termed ExoDinG (Fig. 1 and Table S1). The biological role of ExoDinG is yet to be fully elucidated. In contrast to DinG, ExoDinG homologs are not regulated by LexA and do not participate in the SOS response in *Staphylococcus aureus* [6], [47] and *Bacillus subtilis* [48].

It has been confirmed that ExoDinG from *S. aureus* (Bacillales) exhibits active 3′–5′ exonuclease activity on ssDNA and ssRNA substrates but lacks helicase activity [49]. Notably, multiple sequence alignments indicate that ExoDinG proteins from Bacillales and Lactobacillales contain a pFeS domain, as they lack sufficient C residues (either none or only one) at the putative FeS cluster binding site (Fig. 1 and Fig. S5). The relationship between the absence of helicase activity in *S. aureus* ExoDinG (*Sa*ExoDinG) and its incomplete FeS domain remains unclear. This is particularly intriguing given that both CasDinG and CasDinG-HNH, despite lacking FeS domains altogether, exhibit helicase activity [14], [16]. Further biochemical studies on ExoDinG group members with intact FeS domains could provide valuable insights into the significance of this domain for helicase activity in this subgroup.

**Fig. 6:**
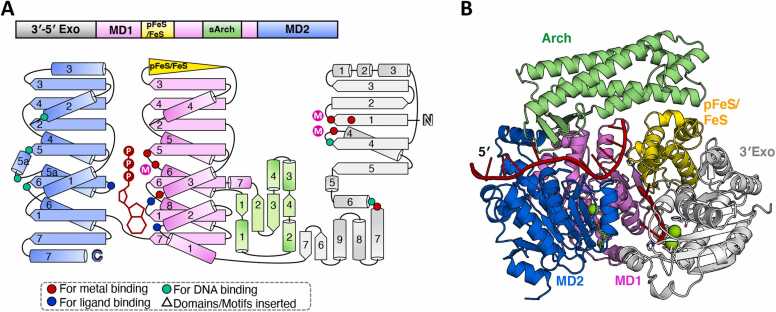
Summary of the predicted structure information of ExoDinG. (A) The domain arrangement and the topology diagram of ExoDinG. ExoDinG from *B. subtilis* was selected for structural analysis within the ExoDinG subgroup proteins. The MD1, MD2, Arch, pFeS/FeS, and 3′–5′ Exonuclease domains were colored violet, marine, lime green, yellow, and white, respectively. Key residues involved in metal ion coordination, ATP binding, and substrate interaction were highlighted as red dots, blue dots, and cyan dots, respectively. (B) The overall structure of the *Bs*ExoDinG–ssDNA–ATP complex, as predicted by AlphaFold3, is depicted in cartoon representation, with each domain colored as described in (A). ssDNA and ATP·Mg^2+^ were colored red and green, respectively. The input information used for model prediction is detailed in the supplementary material.

To date, no structural information on ExoDinG has been reported. Given that both the helicase groove and the N-terminal 3′–5′ exonuclease domain are likely capable of DNA binding, two ssDNA chains were included as input for structural prediction using AlphaFold3. As anticipated, the predicted structure of the *B. subtilis* ExoDinG–ssDNA–ATP complex features two DNA chains (Fig. 6A, B, and Fig. S5). One chain extends across and binds to both MD1 and MD2 (Fig. 6B), where the key residues involved in DNA binding, such as the P motif, aromatic residues, and other structural elements are conserved (Fig. S5). The second chain binds to the NTD, which consists of five β-strands arranged in the order 32145 (↓↑↓↓↓), with three α-helices inserted between β3 and β4, and an additional α-helix inserted between β4 and β5 (Fig. 6A). Following β5, a helix bundle comprising five α-helices is present. A β-hairpin structural element connects the NTD and MD1. The catalytic center contains five conserved amino acids, forming a DEDDh motif, which is commonly found within the 3′–5′ exonuclease family [50]. Specifically, one conserved D residue and one conserved E residue at the C-terminus of β1, one conserved D residue at the N-terminus of α4, and one conserved D residue at the N-terminus of α7 coordinate the divalent cations, followed by one conserved H residue responsible for nucleophilic water activation (Fig. 6A and Fig. S5).

DALI analysis of the NTD of *Bs*ExoDinG revealed a series of DEDDh type 3′–5′ exonuclease with topological similarity (Table S2), including the NTD of *E. coli* Exonuclease I (PDB ID: 4JS4), *E. coli* RNase T (PDB ID: 3V9X), *E. coli* Cap18 (PDB ID: 7T2S), and the ε subunit of *E. coli* DNA polymerase III (PDB ID: 5M1S). Among these, *E. coli* Exonuclease I is an Mg^2+^-dependent 3′–5′ exonuclease involved in a various processes related to DNA repair and recombination, notably methyl-directed mismatch repair, where it is one of three enzymes that can digest the 3′-ended strand containing the mismatched nucleotide [51]. RNase T is responsible for the final trimming of several small stable RNAs during 3′-end maturation, digesting both DNA and RNA, with its activity inhibited by duplex structures and 3′-terminal cytosine [52]. Cap18 is a predicted 3′–5′ exonuclease associated with hundreds of cyclic oligonucleotide-based anti-phages signaling systems (CBASS) [53]. The ε subunit of DNA polymerase III, also known as DnaQ, exhibits 3′–5′ exonuclease activity, which is essential for removing mis-incorporated nucleotides during DNA replication [54].

To sum up, ExoDinG likely plays a role in nucleic acid processing, particularly in DNA repair and RNA maturation, through its 3′–5′ exonuclease activity. Its structural and functional similarities to other exonucleases suggest potential involvement in maintaining genomic stability, degrading damaged or mismatched nucleotides, and participating in anti-phage defense mechanisms. The presence of a helicase groove, despite the lack of observed helicase activity in some members, hints at a possible dual role in nucleic acid unwinding under specific conditions or in certain bacterial lineages. Moreover, further experimental studies are essential to fully elucidate the biological roles and mechanisms of ExoDinG, particularly in relation to its FeS domain variability and potential helicase activity.

### 3.6 pExoDinG

DinG-like proteins from specific bacterial orders possess an additional NTD with a topology strikingly similar to the N-terminal nuclease domain of ExoDinG. However, its putative nuclease center is incomplete that contains only two conserved acidic residues. Hence, this study designated these proteins as pseudo ExoDinG (pExoDinG) (Fig. 1 and Table S1).

**Fig. 7:**
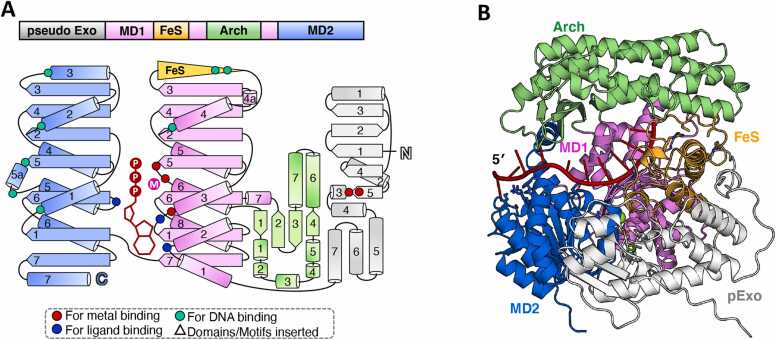
Summary of the predicted structure information of pExoDinG. (A) The domain arrangement and the topology diagram of pExoDinG. pExoDinG from *C. vibrioides* was selected for structural analysis within the pExoDinG subgroup proteins. The MD1, MD2, Arch, FeS, and pseudo exonuclease domains were colored violet, marine, lime green, orange, and white, respectively. Key residues involved in metal ion coordination, ATP binding, and substrate interaction were highlighted as red dots, blue dots, and cyan dots, respectively. (B) The overall structure of the *Cv*pExoDinG–ssDNA–ATP complex, as predicted by AlphaFold3, was shown in cartoon form, with each domain colored as described in (A). ssDNA and ATP·Mg^2+^ were colored red and green, respectively. The input information used for model prediction is detailed in the supplementary material, while the elements (including one ssDNA strand and Mg^2+^ near the supposed nuclease site) that could not be modeled with high-quality have been omitted from this figure for clarity.

As expected, AlphaFold3 modeling of *Caulobacter vibrioides* pExoDinG (*Cv*pExoDinG) failed to identify an active site coordinated with metals, suggesting pExoDinG lacks nuclease activity (Fig. 7A, B, and Fig. S6). Beyond the incomplete nuclease center, the NTD of pExoDinG exhibits distinct features compared to ExoDinG. Specifically, pExoDinG contains only one α-helix inserted between β3 and β4, in contrast to the three α-helices present in ExoDinG (Fig. 7A). Additionally, the β-hairpin structural element linking the NTD and MD1 in ExoDinG is absent in pExoDinG (Fig. 7A, B). However, the ATPase sites and DNA-binding mode in the helicase channel of pExoDinG closely resemble those of *Ec*DinG–ssDNA (PDB ID: 6FWS) and *Pa*CasDinG–ssDNA (PDB ID: 7XF1) (Fig. 7B and Fig. S6). The four C residues in the FeS domain are conserved and might participate in [4Fe–4S] cluster coordination (Fig. S6). These findings suggest that pExoDinG may still retain helicase activity.

To date, no reports have detailed the biochemical activity and biological function of the pExoDinG. A search of the PDB using the predicted NTD structure of *Cv*pExoDinG with DALI revealed that proteins with the highest structural similarity belong to the DEDDy-type exonuclease (Table S2). Among these, the 3′–5′ exonuclease domain of Polymerase I (PDB ID: 1BGX) is involved in removing mis-incorporated nucleotides during DNA replication [55], NanoRNase C (NrnC) (PDB ID: 7MPO) is a 3′–5′ exonuclease that preferentially digests dinucleotides and supports cell growth [56], and Nibbler (Nbr) (7JW6) is a 3′–5′ exoribonuclease whose catalytic 3′-end trimming activity plays a role in microRNA (miRNA) and PIWI-interacting RNA (piRNA) biogenesis [57]. DEDDy-type exonucleases typically contain four conserved acidic residues coordinating two catalytic metal ions and a Y residue responsible for nucleophilic water activation, enabling 3′–5′ exonuclease activity on single-stranded nucleic acids [50]. In pExoDinG, the absence of the C-terminal two acidic residues and the catalytic Y residue in the DEDDy motif likely renders the nuclease core inactive (Fig. S6). However, while catalytically inactive, its NTD could play a regulatory or structural role by binding single-stranded nucleic acids and influencing the function of the helicase domain. Given its structural similarities to DEDDy-type exonucleases, pExoDinG might also participate in pathways related to DNA repair, RNA maturation, or genome stability. Further experimental studies are needed to confirm its precise biological role and mechanisms, particularly its potential helicase activity and the functional significance of its NTD.

### 3.7 EndoDinG

**Fig. 8:**
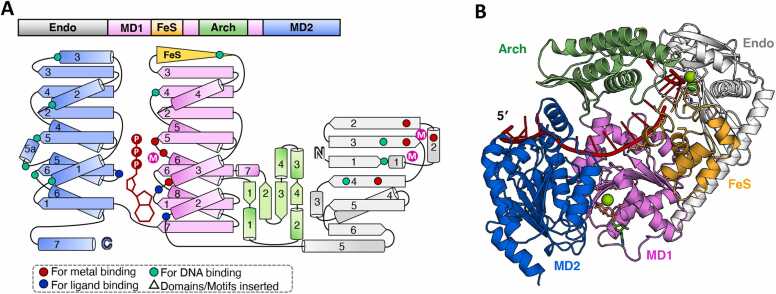
Summary of the predicted structure information of EndoDinG. (A) The domain arrangement and the topology diagram of EndoDinG. EndoDinG from *B. bacteriovorus* HD100 was selected for structural analysis within the EndoDinG subgroup proteins. The MD1, MD2, Arch, FeS, and 3′–5′ Exonuclease/Endonuclease domains were colored violet, marine, lime green, orange, and white, respectively. Key residues involved in metal ion coordination, ATP binding, and substrate interaction were highlighted as red dots, blue dots, and cyan dots, respectively. (B) The overall structure of the *Bb*EndoDinG–ssDNA–ATP complex, as predicted by AlphaFold3, was shown in cartoon form, with each domain colored as described in (A). ssDNA and ATP·Mg^2+^ were colored red and green, respectively. The input information used for model prediction is detailed in the supplementary material.

Some DinG-like proteins possess an additional N-terminal 5′-3′ exonuclease/endonuclease domain (Fig. 8A). Our research group recently verified the biochemical activity of *Bdellovibrio bacteriovorus* HD100 DinG, a representative member of this subgroup, and found that it exhibits endonuclease activity rather than exonuclease activity (unpublished data). As a result, this family of proteins has been designated as EndoDinG.

To date, no structural information on EndoDinG has been reported. Given that both the helicase groove and the N-terminal domain are likely capable of DNA binding, two ssDNA chains were included as input for structural prediction using AlphaFold3. As anticipated, the predicted structure of the *B. bacteriovorus* EndoDinG–ssDNA–ATP complex comprising two DNA chains (Fig. 8A, B, and Fig. S7). One DNA chain extends across and binds to both MD1 and MD2 (Fig. 8B) where the key residues involved in DNA binding, such as the P motif, aromatic residues, and other structural elements are conserved (Fig. S7). Compared to DinG, EndoDinG lacks the β7 strand in MD2 and features a more truncated Arch domain (Fig. 8A, B). The second DNA chain binds to the NTD, which consists of six β-strands and five α-helices, forming a PD-(D/E)XK motif (Fig. 8A, B). Similar to PD-(D/E)XK-type 5′–3′ exonucleases [58], EndoDinG exhibits four conserved amino acids in its N-terminal putative nuclease central region: an H residue in the middle of α2, an E residue at the N-terminus of β2, a D residue at the N-terminus of β3, and an E residue in the middle of β4 (Fig. 8A and Fig. S7). These residues are believed to form a catalytic center that binds catalytic metals and substrates, facilitating substrate hydrolysis. In line with other PD-(D/E)XK members, EndoDinG also contains a conserved lysine at the C-terminus of β4, which is believed to bind the scissile phosphate of the substrate (Fig. 8A and Fig. S7) [58].

DALI analysis identified proteins with the highest structural similarity to this NTD as members of the 5′–3′ exo/endonuclease family containing the PD-(D/E)XK motif (Table S2), including *E. coli* RecE (PDB ID: 3H4R), human EXO5 (PDB ID: 7LW8), CRISPR-associated Cas4 (PDB ID: 7MI4), *B. subtilis* AddA (PDB ID: 4CEJ), and mouse Dna2 (PDB ID: 5EAN). *E. coli* RecE binds dsDNA ends, digesting the 5′ strand to form 5′ mononucleotides and a 3′ overhang for RecT-mediated single-strand annealing [59]. Human EXO5, an ssDNA-specific exonuclease with an FeS cluster, acts as an adenosine triphosphate-ribonucleic acid (ATR)-regulated nuclease and bloom syndrome protein (BLM) partner for replication fork restart [60]. Cas4, part of CRISPR-Cas adaptation modules, exhibits 5′–3′ exo/endonuclease activities, cleaving DNA to integrate new genetic material into CRISPR arrays [61]. The bacterial AddAB complex unwinds and digests DNA from broken ends until encountering a χ sequence, producing a 3′ ssDNA tail for RecA loading [62]. Dna2 plays important roles in DNA replication, repair and processing of stalled replication forks [63]. These functional parallels suggest that EndoDinG is likely involved in DNA repair and replication processes, utilizing its endonuclease activity to cleave DNA and its helicase activity to unwind DNA structures.

EndoDinG contains a conserved FeS domain characterized by a CCCC motif that coordinates a [4Fe-4S] cluster (Fig. 8 and Fig. S7). Notably, many PD-(D/E)XK-type 5′–3′ exonucleases, such as EXO5, Cas4, AddA, and Dna2, also contain FeS clusters. Disruption of the FeS cluster in human EXO5 abrogates its nuclease activity [60], while prolonged exposure to air induces indiscriminate DNA cleavage by Cas4, likely due to FeS cluster oxidation [61]. In *B. subtilis* AddA, the FeS cluster is essential for DNA double-strand break binding and digestion [64], [65]. Similarly, loss of the FeS cluster in human Dna2 impairs its DNA binding, digestion, helicase, and ATPase activities [63]. Although the putative FeS cluster’s location in EndoDinG differs from these proteins, it is likely that the FeS cluster in EndoDinG plays a critical role in stabilizing the protein structure, facilitating DNA binding, and modulating its nuclease activity.

### 3.8 RadC-like DinG

**Fig. 9:**
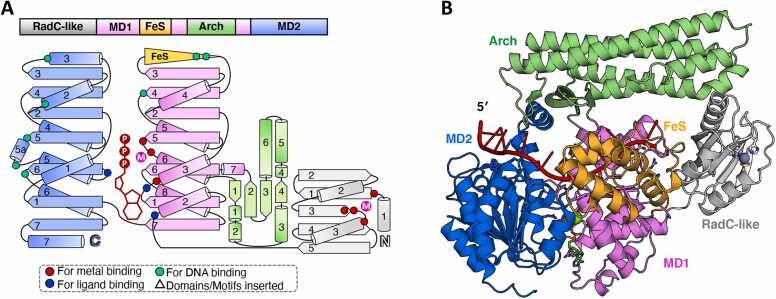
Summary of the predicted structure information of RadC-like DinG. (A) The domain arrangement and the topology diagram of RadC-like DinG. RadC-like DinG from *D. soudanensis* was selected for structural analysis within the RadC-like DinG subgroup proteins. The MD1, MD2, arch, FeS, and RadC-like domains were colored violet, marine, lime green, orange, and white, respectively. Key residues involved in metal ion coordination, ATP binding, and substrate interaction were highlighted as red dots, blue dots, and cyan dots, respectively. (B) The overall structure of the *Ds*RadC-like DinG–ssDNA–ATP complex, as predicted by AlphaFold3, was shown in cartoon form, with each domain colored as described in (A). ssDNA, ATP·Mg^2+^, Zn^2+^ were colored red, green, and purple blue respectively. The input information used for model prediction is detailed in the supplementary material.

Certain DinG-like proteins contain an additional N-terminal RadC-like domain, referred to as RadC-like DinG (Fig. 1 and Fig. 9A). To date, no research has been conducted on RadC-like DinG. However, the function of RadC appears to be unrelated to DNA repair, as *E. coli radC* mutants demonstrate normal sensitivity to DNA-damaging agents [66].

Sequence alignment analysis revealed high conservation among these RadC-like DinG proteins (Fig. S8). Secondary structure analysis and structural modeling were performed using *Desulfuromonas soudanensis* RadC-like DinG (*Ds*RadC-like DinG), the first protein in the alignment file (Fig. S8). Except for the presence of the RadC-like domain, the predicted overall architecture of *Ds*RadC-like DinG demonstrates substantial structural similarity with YoaA. While RadC-like DinG retains the characteristic C-terminal α7 helix within the MD2 domain, similar to YoaA, this helix likely servers a distinct function rather than mediating interactions with HolC (Fig. 9A). This is supported by the absence of HolC homologs in organisms encoding RadC-like DinG (Table S1). The residues critical for metal coordination, ATP binding, and DNA binding in both MD1 and MD2 are fully conserved, enabling accurate modeling of ssDNA and ATP·Mg²⁺ in the predicted structure (Fig. 9B and Fig. S8).

The RadC-like domain comprises five β-strands arranged in the order of 21345 (↑↓↑↑↓), flanked by α-helical structural elements. Four conserved amino acids were identified in the central region, potentially forming a catalytic center: one E residue at the N-terminus of β1, two H residues at the C-terminus of β1, and one D residue at the N-terminus of α2 (Fig. 9A and Fig. S8). DALI analysis of the RadC-like domain revealed no similarity to nucleases but identified metalloproteases as topologically related proteins (Table S2). For example, *Pyrococcus furiosus* JAMM/MPN^+^ metalloprotease (*Pf*JAMM1) (PDB ID: 5LDA) cleaves (iso)peptide bonds C-terminal to ubiquitin (Ub) and ubiquitin-like protein (Ubl) domains [67], while *Caldiarchaeum subterraneum* Rpn11 (*Cs*Rpn11) (PDB ID: 6FJU) processes the CsUb precursor and ubiquitinated proteins, regulating ubiquitination levels [68]. Based on the Zn²⁺ ion preference in *Pf*JAMM1 and *Cs*Rpn11, a Zn²⁺ ion was modeled within the putative catalytic center of the RadC-like domain (Fig. 9B and Fig. S8). Further studies are needed to elucidate the functional role of the RadC-like domain and its potential catalytic activity.

### 3.9 sDinG (short-type DinG)

**Fig. 10:**
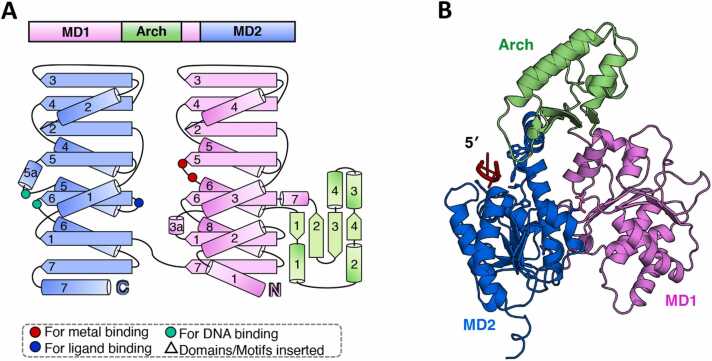
Summary of the predicted structure information of sDinG. (A) The domain arrangement and the topology diagram of sDinG. sDinG from *Nostoc sp*. PCC 7120 was selected for structural analysis within the sDinG subgroup proteins. The MD1, MD2, and Arch domains were colored violet, marine and lime green, respectively. Key residues involved in metal ion coordination, and substrate interaction were highlighted as red dots, blue dots, and cyan dots, respectively. (B) The overall structure of *Nos*sDinG–ssDNA complex, as predicted by AlphaFold3, was shown in cartoon form, with each domain colored as described in (A). ssDNA was colored red. The input information used for model prediction is detailed in the supplementary material, while the elements (include a portion of the ssDNA strand and ATP·Mg^2+^) that could not be modeled with high-quality have been omitted from this figure for clarity.

A distinct group of short-type DinG (sDinG) homologs has been identified, characterized by the presence of only three domains: the MD1 domain, MD2 domain, and the Arch domain (Fig. 10A). These sDinG homologs are found in the bacterial orders Nostocales, Chroococcidiopsidales, and Pleurocapsales (Fig. 1 and Table S1).

To date, no studies have investigated the function or biochemical activity of sDinG. Secondary structure analysis and structural modeling were performed using *Nostoc sp*. PCC 7120 sDinG (*Nos*sdDinG) (Fig. S9). Based on the *Ec*DinG–ssDNA–ATP complex structure, an 11-nt poly dT ssDNA and an ATP·Mg²⁺ molecule were included as input for the prediction of the *Nos*sDinG–ssDNA–ATP complex structure. However, the resulting model showed poor metrics for both the ATP and DNA molecules (Fig. S9). The ATP molecule could not be accurately modeled in the putative ATPase site, likely due to the absence of conserved ATP-binding residues and the presence of an additional α-helix (α3a) between β1 and α3, which appears to occupy the ATP-binding site and hinder ATP binding (Fig. 10A and Fig. S9). These structural features raise uncertainty about the functional capacity of sDinG as an active ATPase (Fig. S9). Furthermore, due to the absence of conserved DNA-binding residues on MD1, only a partial DNA complex bound to MD2 could be predicted using AlphaFold3 (Fig. 10B and Fig. S9). In addition to lacking an FeS domain, the Arch domain of *Nos*sDinG displays a significantly more compact α-helix bundle compared to that of *Ec*DinG (Fig. 10A).

In summary, sDinG likely represents a functionally distinct or specialized variant of DinG-like proteins. Its truncated Arch domain, lack of an FeS domain, and incomplete ATP and DNA binding sites suggest that sDinG may not function as a traditional helicase or ATP-dependent DNA-processing enzyme. Further experimental studies are essential to elucidate its precise biological role, particularly in light of its unique domain architecture and conserved structural features.

### 3.10 Others

In addition to the aforementioned categories of DinG-like homologs, some bacterial DinG variants exhibit unique structural alterations, such as insertions or truncations of particular motifs or domains. These modifications prevent their classification within any of the previously discussed categories, and therefore they have discussed separately in this section of the review (Fig. 1 and Table S1). Although the biochemical properties and *in vivo* functions of these proteins remain unexplored, in this review we aim at generating structural predictions of these proteins in complex with ssDNA and ATP to generate novel insights on their possible role in bacteria (Fig. S10).

**Fig. 11:**
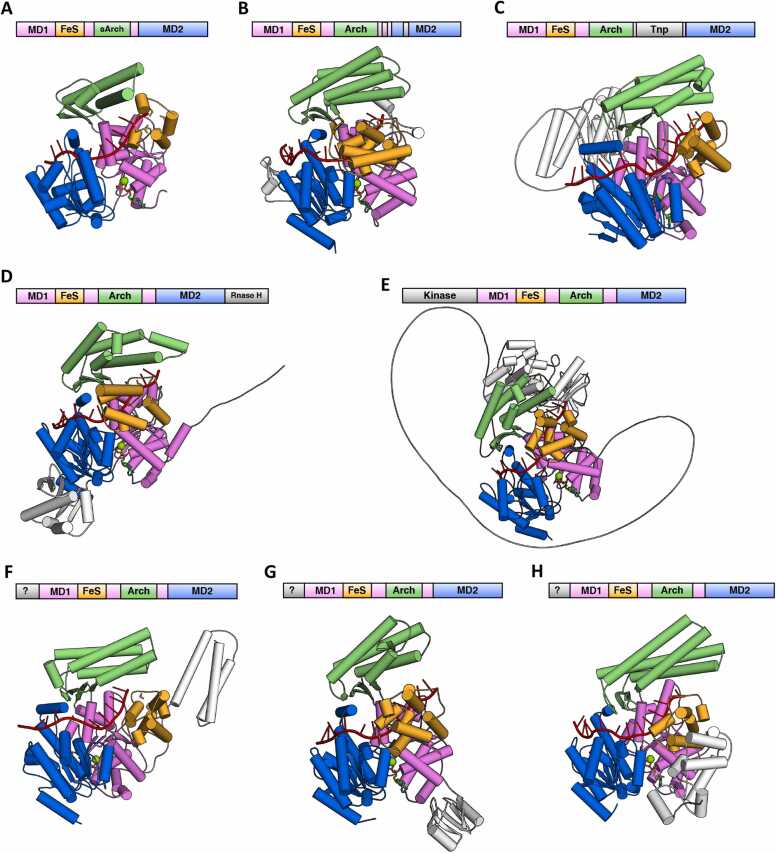
Summary of the predicted structure information of the other undefined subgroups of DinG-like proteins. The domain arrangements and the predicted DNA and ATP·Mg^2+^ bound complex structures of DinG-like proteins from bacteria orders *Truepera radiovictrix* (order Trueperales) (A), *Nakamurella multipartite* (order Nakamurellales) (B), *Humisphaera borealis* (order Tepidisphaerales) (C), *Caldilinea aerophila* (order Caldilineales) (D), Ktedonobacterales bacterium SCAWS-G2 (order Ktedonobacterales) (E), *Desulfotalea psychrophila* (order Desulfobacterales) (F), *Hippea maritima* (order Desulfurellales) (G), and *Kosmotoga olearia* (order Kosmotogales) (H) were depicted. The MD1, MD2, FeS, Arch, and the additional domains were colored violet, marine, lime green, and white, respectively. AlphaFold3 predicted structures were shown as cartoon. The ssDNA and ATP·Mg^2+^ were colored red and green, respectively.

The DinG-like protein from *Truepera radiovictrix* (order Trueperales) features a notably compact Arch domain compared to DinG, consisting of three α-helices and three β-sheets, which may confer distinct biochemical properties relative to DinG (Fig. 11A). The DinG-like protein from *Nakamurella multipartita* (order Nakamurellales) contains two distinct insertions within its helicase motor domains. The first insertion comprises two α-helices positioned between α7 and β6 of MD1, while the second insertion consists of a β-hairpin and an α-helix located between β1 and α1 of MD2 (Fig. 11B). Phylogenetic analysis suggests these two types of DinG-like proteins are more closely related to pExoDinG (Fig. 1), despite their apparent lack of the pseudo-exonuclease domain.

The DinG-like protein from *Humisphaera borealis* (order Tepidisphaerales) incorporates a transposase-like domain between β6 and α8 of MD1, while maintaining high structural similarity with DinG in other regions (Fig. 11C). DALI analysis identified that this transposase-like domain exhibits the highest topological similarity with *E. coli* MG1655 TnpA (PDB ID: 4ER8), a protein closely associated with the IS200/IS605 family (Table S2) [69].

Some bacteria from Caldilineales, such as *Caldilinea aerophile*, harbor two DinG-like proteins (Fig. 1 and Table S1). One belongs to the ExoDinG subgroup, while the other includes an additional RNase H-like domain at its C-terminus, making it difficult to classify (Fig. 11D). DALI analysis revealed that this RNase H-like domain shares high similarity with the *Thermus thermophilus* HB27 argonaute protein (*Tt*Ago) (PDB ID: 5XOU), which play a crucial role in RNA-silencing pathways (Table S2) [70].

Similarly, some bacteria from Ktedonobacterales, such as *Ktedonobacterales bacterium* SCAWS-G2, also harbors two DinG-like proteins (Fig. 1 and Table S1). One belongs to the ExoDinG subgroup, while the other possesses an extra kinase-like domain at its C-terminus, making it difficult to classify (Fig. 11E). DALI analysis revealed that this kinase-like domain shares high similarity with the *M. tuberculosis* Ser/Thr kinase PknA (PDB ID: 4X3F) (Table S2), which is essential for Ser/Thr phosphorylation and plays critical roles in signal transduction [71].

Phylogenetic analysis suggests that the three aforementioned DinG-like proteins (from Tepidisphaerales, Caldilineales, and Ktedonobacterales) are more closely related to the DinG subgroup (Fig. 1). The transposase-like, RNase H-like and kinase-like domains may represent additional structural domains that the DinG subgroup protein acquired incidentally during long-term evolution.

DinG-like proteins from Kosmotogales, Desulfurellales, and Desulfobacterales feature an additional NTD (Fig. 11F–H). These NTDs exhibit limited structural similarity to known proteins, and their functions remain uncharacterized. Among them, DinG-like proteins from Kosmotogales and Desulfurellales are phylogenetically closer to the DinG subgroup, while those from Desulfobacterales are more closely related to the YoaA subgroup (Fig. 1).

## 4 Conclusions and perspectives

This review provides a systematic analysis of bacterial DinG-like proteins, classifying them into distinct subgroups and elucidating their evolutionary trajectories and biological functions. These proteins, widely distributed across bacterial taxa, exhibit remarkable diversity in sequence, structure, biochemical properties, and function. However, limitations remain in the current classification system and structural predictions, leaving several unresolved questions.

Bacteria often encode multiple DinG-like homologs (Fig. 1 and Table S1), potentially conferring specialized functions such as phage defense or compensating for the loss of other proteins in essential pathways. Similarly, eukaryotic homologs exhibit distinct structural features, subcellular localizations, and interactions, enabling specialized roles. For example, xeroderma pigmentosum complementation group D (XPD), a component of transcription factor IIH (TFIIH), participates in transcription initiation and nucleotide excision repair (NER) [72], [73]; Fanconi′s anaemia complementation group J (FancJ) interacts with breast cancer-susceptibility protein BRCA1 in DNA cross-link damage repair [74]; Regulator of telomere length protein 1 (RTEL1) safeguards telomere integrity and genomic stability by suppressing homologous recombination [75]; and Chl1 is crucial for DNA repair and sister chromatid segregation [76]. The functional diversification of bacterial DinG-like proteins suggests evolutionary adaptation for enhanced cellular processes and network connectivity. Future studies integrating functional assays and comparative genomics are needed to refine our understanding of their roles.

Current classification relies primarily on domain architecture and sequence similarity, yet this approach has inherent limitations. Many DinG-like proteins remain experimentally uncharacterized, leading to classifications based largely on sequence alignment and predicted structural element, which may not accurately reflect their biological roles. In particular, the distinction between DinG and YoaA subgroups remains ambiguous. Moreover, with the rapid expansion of bacterial genomic data, novel DinG-like proteins with unique domains are continuously identified, challenging the existing framework. Presently, unclassifiable proteins are categorized as "others," but future efforts incorporating experimental validation and machine learning-based refinements may yield a more robust classification system.

Structural and functional predictions, primarily derived from computational tools like AlphaFold3, also present challenges. Their accuracy depends on the presence of cofactors and interaction partners, necessitating biochemical validation. While computational models provide valuable insights, they often fail to capture dynamic conformational changes and interactions *in vivo*. Integrating experimental data with structural predictions will be crucial for a deeper understanding of DinG-like proteins in bacterial physiology.

The FeS domain in DinG-like proteins typically features a conserved CCCC motif coordinating a [4Fe-4S] cluster, though some variants (e.g., CCHC, CCCS) exist, whose ability to bind FeS clusters requires experimental verification. Interestingly, CCCC and CCHC motifs also appear in Zn-finger domains, suggesting that certain FeS domains might bind Zn rather than FeS clusters. The functional role of the FeS cluster appears to vary among DinG-like proteins—ranging from involvement in redox signaling to structural stabilization. Notably, in certain DinG-like proteins, such as CasDinG and CasDinG-HNH, the FeS cluster-binding ability has been lost, yet these domains retain structural roles and contribute to DNA binding and translocation [14], [16]. Future biochemical and structural studies will be essential for elucidating the precise functional divergence of FeS clusters within different DinG-like subgroups.

The Arch domain of DinG-like proteins also exhibits variable length and geometry, yet its function remains poorly understood. Although it does not directly bind DNA in most cases, it may modulate overall protein structure and function through interdomain interactions. For instance, in *Ec*DinG, the Arch domain connects to MD2 via a spring-helix (α5a), facilitating DNA translocation and unwinding [17]. Conversely, DinG-like proteins such as CasDinG-HNH feature a truncated Arch domain, potentially affecting their functionality. Future research leveraging structural biology and molecular dynamics simulations will be critical to deciphering the role of the Arch domain in DinG-like proteins.

## Funding

This work was funded by the grants from the 10.13039/501100004731Natural Science Foundation of Zhejiang Province to KC (LQ22C050002), the 10.13039/501100001809National Natural Science Foundation of China to KC (32270043 and 32100017), the Hangzhou Youth Innovation Team Project to KC (TD2023020), and the Scientific Research Foundation for Scholars of HZNU to KC (4125C50221204040).

## CRediT authorship contribution statement

**Kaiying Cheng:** Writing – review & editing, Writing – original draft, Supervision, Software, Project administration, Investigation, Conceptualization.

## Declaration of Competing Interest

The authors declare that they have no known competing financial interests or personal relationships that could have appeared to influence the work reported in this paper.
