# Supplementary material for: Structure, function and evolution of the bacterial DinG-like proteins
Source: Comput Struct Biotechnol J. 2025 Mar 17;27:1124–39. doi: 10.1016/j.csbj.2025.03.023 (PMC11981726; doi:10.1016/j.csbj.2025.03.023)
Supplement: Figure S1 — Supplementary material [file mmc1.pdf]

Figure S1 Supplemental information for DinG subgroup proteins.

A. The structural model of *E. coli* DinG–ssDNA–SSB-Ct complex in the presence of ATP·Mg^{2+} was predicted using AlphaFold3. The input parameters, including protein sequences, substrate information, and ligand specifications, are detailed in the corresponding figure. The model′s quality assessment metrics - predicted local Distance Difference Test (pIDDT), interface predicted TM-score (ipTM), predicted TM-score (pTM), and expected positional error (EPA) - are presented alongside the structural prediction.

B. Multiple sequence alignment of DinGs was performed using Clustal Omega and visualized by ESPript. The names of corresponding bacteria species, protein IDs, and protein sequences were provided in Table S1. Secondary structural elements were depicted based on the experimental determined EcDinG-ssDNA complex structure (PDB ID: 6FRW), displayed at the top of the sequences, numbered, and colored according to domain arrangement. Critical residues for metal coordination, ATP binding, DNA binding, and the P motif were highlighted in red, blue, cyan and brown boxes, respectively.

A

Input	Co pies	Sequence
*Ec*DinG	1	MALTAALKAQIAAWYKALQEQIPDFIPRAPQRQMIADVAKTLAGEEGRHLAIEAPTGVGKTLSYLIPGIAIAREEQKTLVVSTANV ALQDQIYSKDLPLLKKIIPDLKFTAAFGRGRYVCPRNLTALASTEPTQQDLLAFLDDELTPNNQEEQKRCAKLKGDLDTYKWDG LRDHTDIAIDDDLWRRLSTDKASCLNRNCYYYRECPFFVARREIQEAEVVVANHALVMAAMESEAVLPDPKNLLLVLDEGHHLP DVARDALEMSAEITAPWYRLQLDLFTKLVATCMEQFRPKTIPPLAIPERLNAHCEELYELIASLNNILNLYMPAGQEAEHRFAMGE LPDEVLEICQRLAKLTEMLRGLAELFLNDLSEKTGSHDIVRLHRLILQMNRALGMFEAQSKLWRLASLAQSSGAPVTKWATREE REGQLHLWFHCVGIRVSDQLERLLWRSIPHIIVTSATLRSLNSFSRLQEMSGLKEKAGDRFVALDSPFNHCEQGKIVIPRMRVEPSI DNEEQHIAEMAAFFRKQVESKKHLGMLVLFASGRAMQRFLDYVTDLRLMLLVQGDQPRYRLVELHRKRVANGERSVLVGLQS FAEGLDLKGDLLSQVHIHKIAFPPIDSPVVITEGEWLKSLNRYPFEVQSLPSASFNLIQQVGRLIRSHGCWGEVVIYDKRLLTKNY GKRLLDALPVFPIEQPEVPEGIVKKKEKTKSPRRRRR
DNA	1	TTTTTTTTTTT
Ligand	1	ATP
Ion	1	Mg
EcSSBct	1	DDDIPF

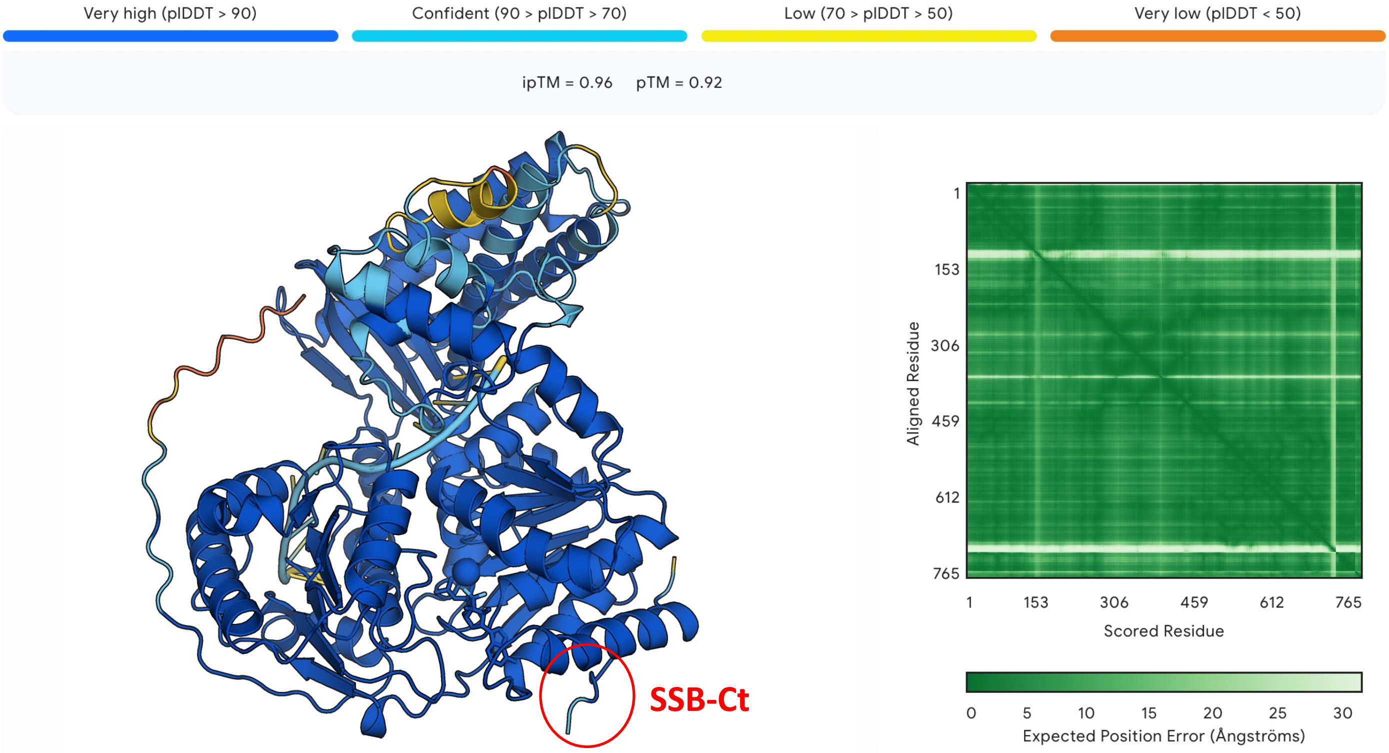

B

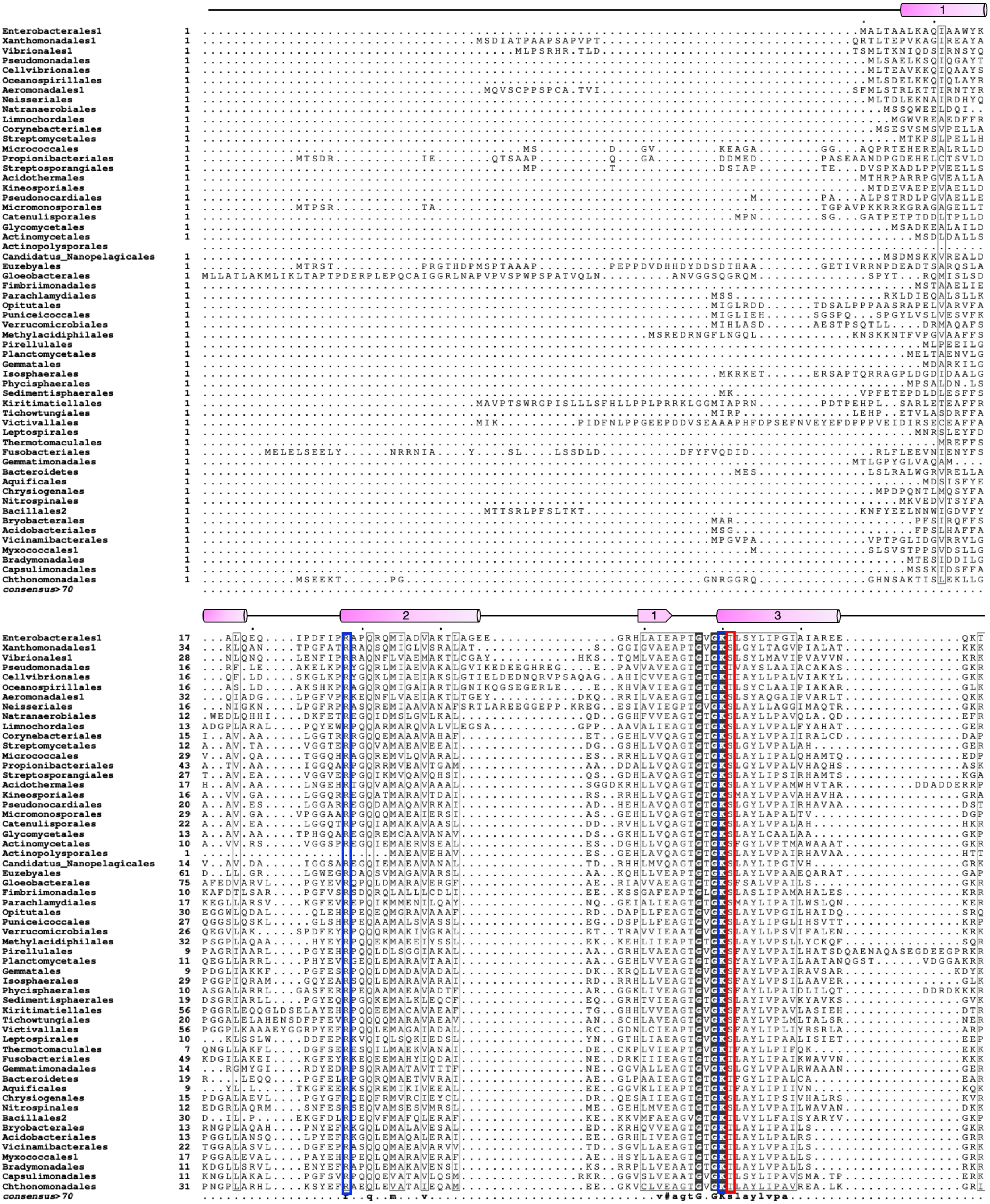

Enterobacterales1
Xanthomonadales1
Vibrionales1
Pseudomonadales
Cellvibrionales
Oceanospirillales
Aeromonadales1
Neisseriales
Natranaerobiales
Limnochordales
Corynebacteriales
Streptomycetales
Micrococcales
Propionibacteriales
Streptosporangiales
Acidothermales
Kineosporiales
Pseudonocardiales
Micromonosporales
Catenulisporales
Glycomycetales
Actinomycetales
Actinopolysporales
Candidatus_Nanopelagicales
Euzebyales
Gloeobacterales
Fimbriimonadales
Parachlamydiales
Opitutales
Puniceicoccales
Verrucomicrobiales
Methylacidiphilales
Pirellulales
Planctomycetales
Gemmatales
Isosphaerales
Phycisphaerales
Sedimentisphaerales
Kiritimatiellales
Tichowtungiales
Victivallales
Leptospirales
Thermotomaculales
Fusobacteriales
Gemmatimonadales
Bacteroidetes
Aquificales
Chrysiogenales
Nitrospinales
Bacillales2
Bryobacterales
Acidobacteriales
Vicinamibacterales
Myxococcales1
Bradymonadales
Capsulimonadales
Chthonomonadales
consensus>70
1
2
1
3
v#agtG.GKslaylvpa

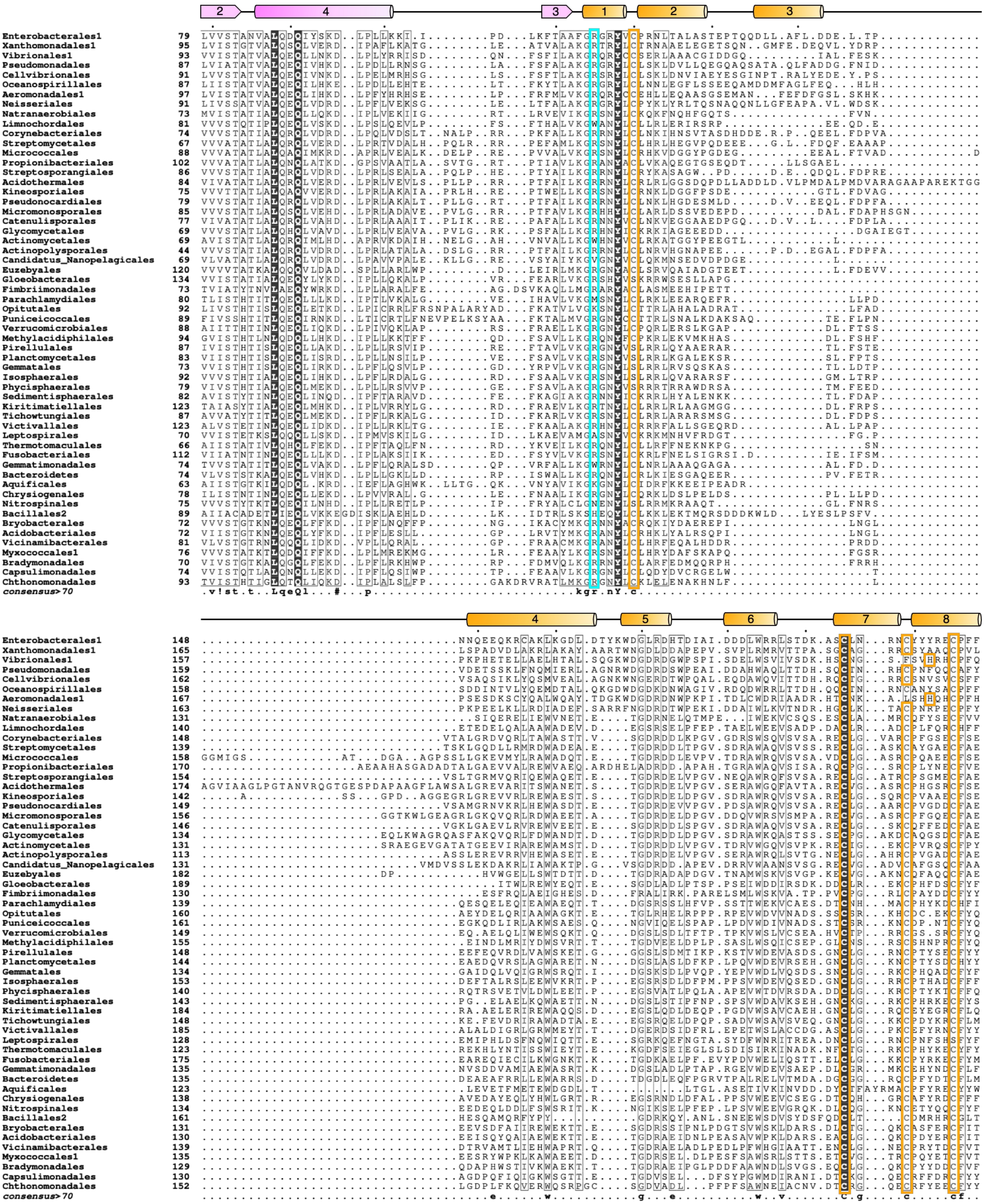

Enterobacterales1
Xanthomonadales1
Vibrionales1
Pseudomonadales
Cellvibrionales
Oceanospirillales
Aeromonadales1
Neisseriales
Natranaerobiales
Limnochordales
Corynebacteriales
Streptomycetales
Micrococcales
Propionibacteriales
Streptosporangiales
Acidothermales
Kineosporiales
Pseudonocardiales
Micromonosporales
Catenulisporales
Glycomycetales
Actinomycetales
Actinopolysporales
Candidatus_Nanopelagicales
Euzebyales
Gloeobacterales
Fimbriimonadales
Parachlamydiales
Opitutales
Puniceicoccales
Verrucomicrobiales
Methylacidiphilales
Pirellulales
Planctomycetales
Gemmatales
Isosphaerales
Phycisphaerales
Sedimentisphaerales
Kiritimatiellales
Tichowtungiales
Victivallales
Leptospirales
Thermotomaculales
Fusobacteriales
Gemmatimonadales
Bacteroidetes
Aquificales
Chrysiogenales
Nitrospinales
Bacillales2
Bryobacterales
Acidobacteriales
Vicinamibacterales
Myxococcales1
Bradymonadales
Capsulimonadales
Chthonomonadales
consensus>70

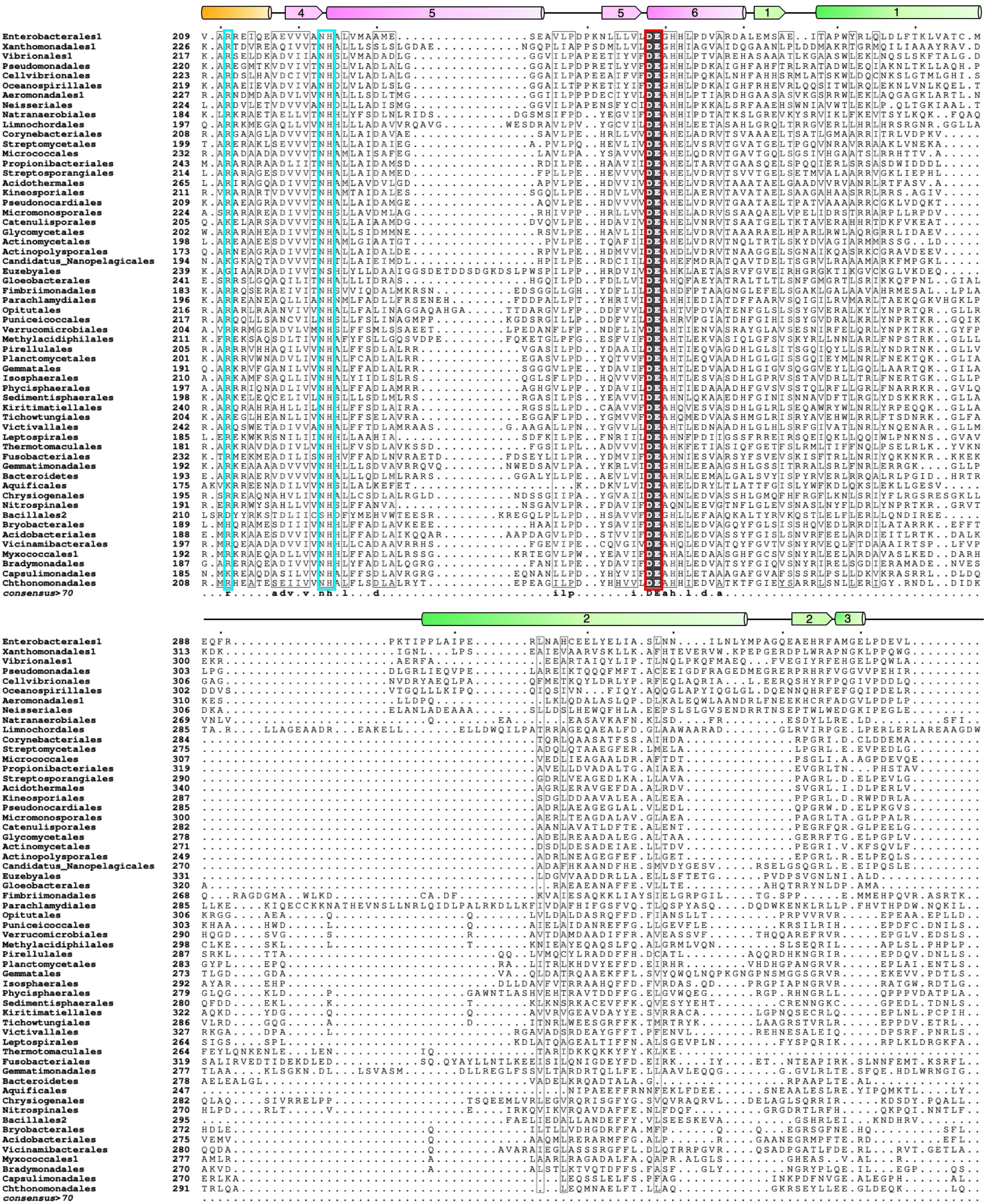
4
5
5
6
1
1
2
2
3
Enterobacterales1
Xanthomonadales1
Vibrionales1
Pseudomonadales
Cellvibrionales
Oceanospirillales
Aeromonadales1
Neisseriales
Natranaerobiales
Limnochordales
Corynebacteriales
Streptomycetales
Micrococcales
Propionibacteriales
Streptosporangiales
Acidothermales
Kineosporiales
Pseudonocardiales
Micromonosporales
Catenulisporales
Glycomycetales
Actinomycetales
Actinopolysporales
Candidatus_Nanopelagicales
Euzebyales
Gloeobacterales
Fimbriimonadales
Parachlamydiales
Opitutales
Puniceicoccales
Verrucomicrobiales
Methylacidiphilales
Pirellulales
Planctomycetales
Gemmatales
Isosphaerales
Phycisphaerales
Sedimentisphaerales
Kiritimatiellales
Tichowtungiales
Victivallales
Leptospirales
Thermotomaculales
Fusobacteriales
Gemmatimonadales
Bacteroidetes
Aquificales
Chrysiogenales
Nitrospinales
Bacillales2
Bryobacterales
Acidobacteriales
Vicinamibacterales
Myxococcales1
Bradymonadales
Capsulimonadales
Chthonomonadales
consensus>70

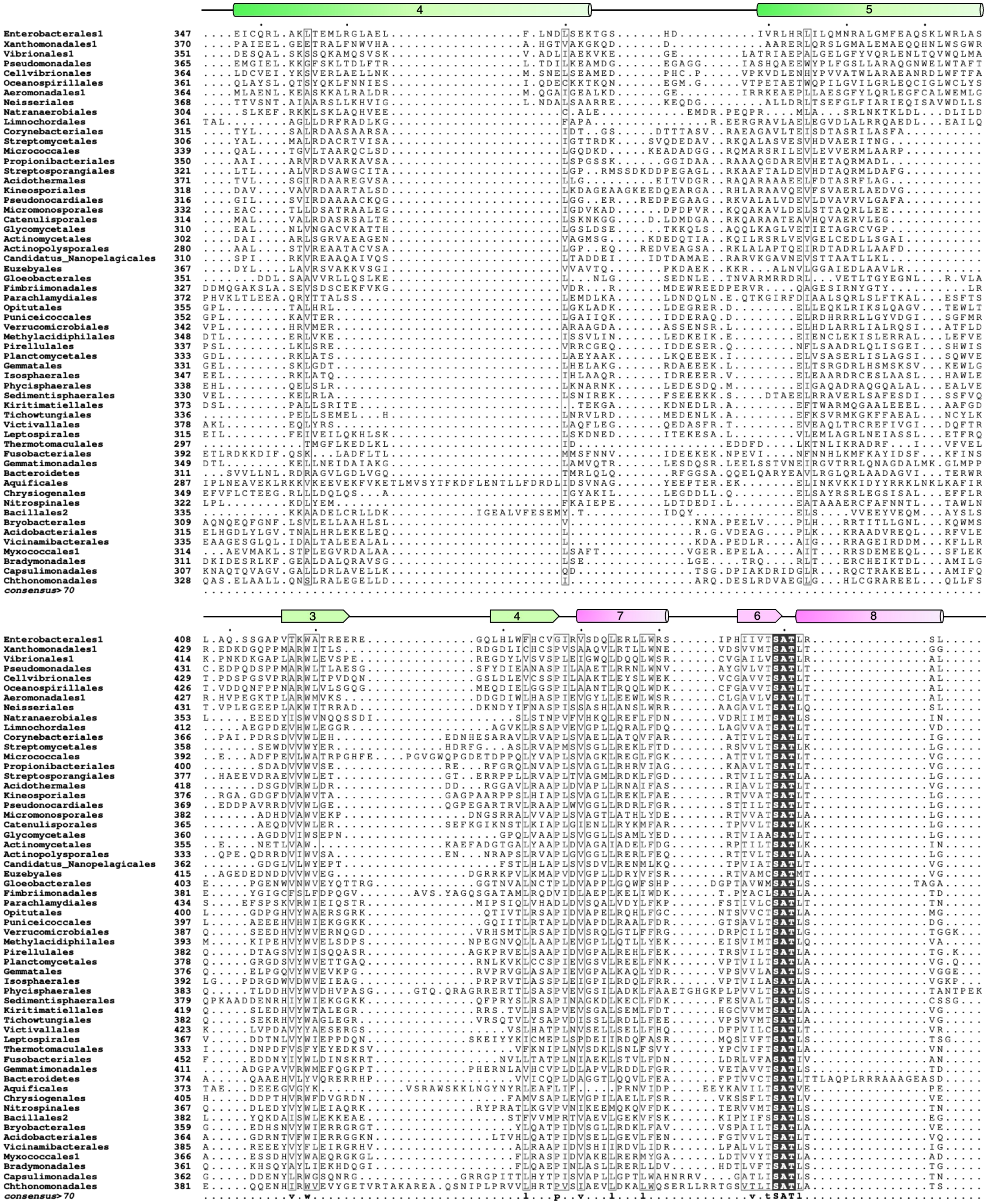
4
5
Enterobacterales1 347
Xanthomonadales1 370
Vibrionales1 351
Pseudomonadales 365
Cellvibrionales 364
Oceanospirillales 361
Aeromonadales1 364
Neisseriales 368
Natranaerobiales 304
Limnochordales 361
Corynebacteriales 315
Streptomycetales 306
Micrococcales 339
Propionibacteriales 350
Streptosporangiales 321
Acidothermales 371
Kineosporiales 318
Pseudonocardiales 316
Micromonosporales 332
Catenulisporales 314
Glycomycetales 310
Actinomycetales 302
Actinopolysporales 280
Candidatus_Nanopelagicales 310
Euzebyales 367
Gloeobacterales 351
Fimbriimonadales 327
Parachlamydiales 372
Opitutales 355
Puniceicoccales 352
Verrucomicrobiales 342
Methylacidiphilales 348
Pirellulales 337
Planctomycetales 333
Gemmatales 331
Isosphaerales 347
Phycisphaerales 338
Sedimentisphaerales 330
Kiritimatiellales 373
Tichowtungiales 336
Victivallales 378
Leptospirales 315
Thermotomaculales 297
Fusobacteriales 392
Gemmatimonadales 349
Bacteroidetes 311
Aquificales 287
Chrysiogenales 349
Nitrospinales 322
Bacillales2 335
Bryobacterales 309
Acidobacteriales 315
Vicinamibacterales 335
Myxococcales1 314
Bradymonadales 311
Capsulimonadales 307
Chthonomonadales 328
consensus>70
3
4
7
6
8
Enterobacterales1 408
Xanthomonadales1 429
Vibrionales1 414
Pseudomonadales 431
Cellvibrionales 429
Oceanospirillales 426
Aeromonadales1 427
Neisseriales 431
Natranaerobiales 353
Limnochordales 412
Corynebacteriales 366
Streptomycetales 358
Micrococcales 392
Propionibacteriales 400
Streptosporangiales 377
Acidothermales 418
Kineosporiales 376
Pseudonocardiales 369
Micromonosporales 382
Catenulisporales 365
Glycomycetales 360
Actinomycetales 355
Actinopolysporales 333
Candidatus_Nanopelagicales 362
Euzebyales 415
Gloeobacterales 403
Fimbriimonadales 381
Parachlamydiales 434
Opitutales 400
Puniceicoccales 397
Verrucomicrobiales 387
Methylacidiphilales 393
Pirellulales 382
Planctomycetales 378
Gemmatales 376
Isosphaerales 392
Phycisphaerales 383
Sedimentisphaerales 379
Kiritimatiellales 419
Tichowtungiales 382
Victivallales 423
Leptospirales 367
Thermotomaculales 333
Fusobacteriales 452
Gemmatimonadales 411
Bacteroidetes 374
Aquificales 373
Chrysiogenales 405
Nitrospinales 367
Bacillales2 382
Bryobacterales 359
Acidobacteriales 364
Vicinamibacterales 385
Myxococcales1 366
Bradymonadales 361
Capsulimonadales 362
Chthonomonadales 381
consensus>70

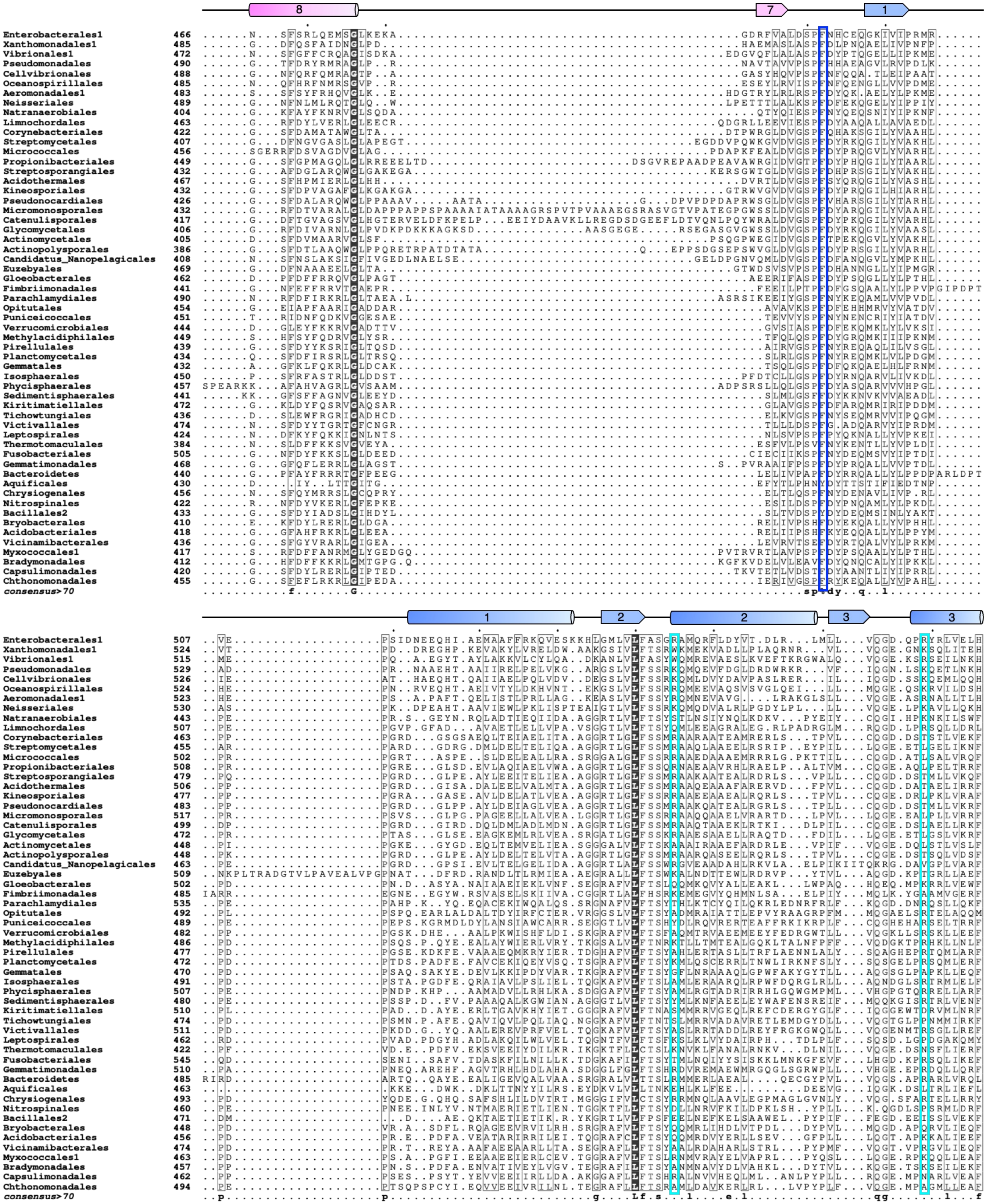
8
7
1
2
3
Enterobacterales1
Xanthomonadales1
Vibrionales1
Pseudomonadales
Cellvibrionales
Oceanospirillales
Aeromonadales1
Neisseriales
Natranaerobiales
Limnochordales
Corynebacteriales
Streptomycetales
Micrococcales
Propionibacteriales
Streptosporangiales
Acidothermales
Kineosporiales
Pseudonocardiales
Micromonosporales
Catenulisporales
Glycomycetales
Actinomycetales
Actinopolysporales
Candidatus_Nanopelagicales
Euzebyales
Gloeobacterales
Fimbriimonadales
Parachlamydiales
Opitutales
Puniceicoccales
Verrucomicrobiales
Methylacidiphilales
Pirellulales
Planctomycetales
Gemmatales
Isosphaerales
Phycisphaerales
Sedimentisphaerales
Kiritimatiellales
Tichowtungiales
Victivallales
Leptospirales
Thermotomaculales
Fusobacteriales
Gemmatimonadales
Bacteroidetes
Aquificales
Chrysiogenales
Nitrospinales
Bacillales2
Bryobacterales
Acidobacteriales
Vicinamibacterales
Myxococcales1
Bradymonadales
Capsulimonadales
Chthonomonadales
consensus>70

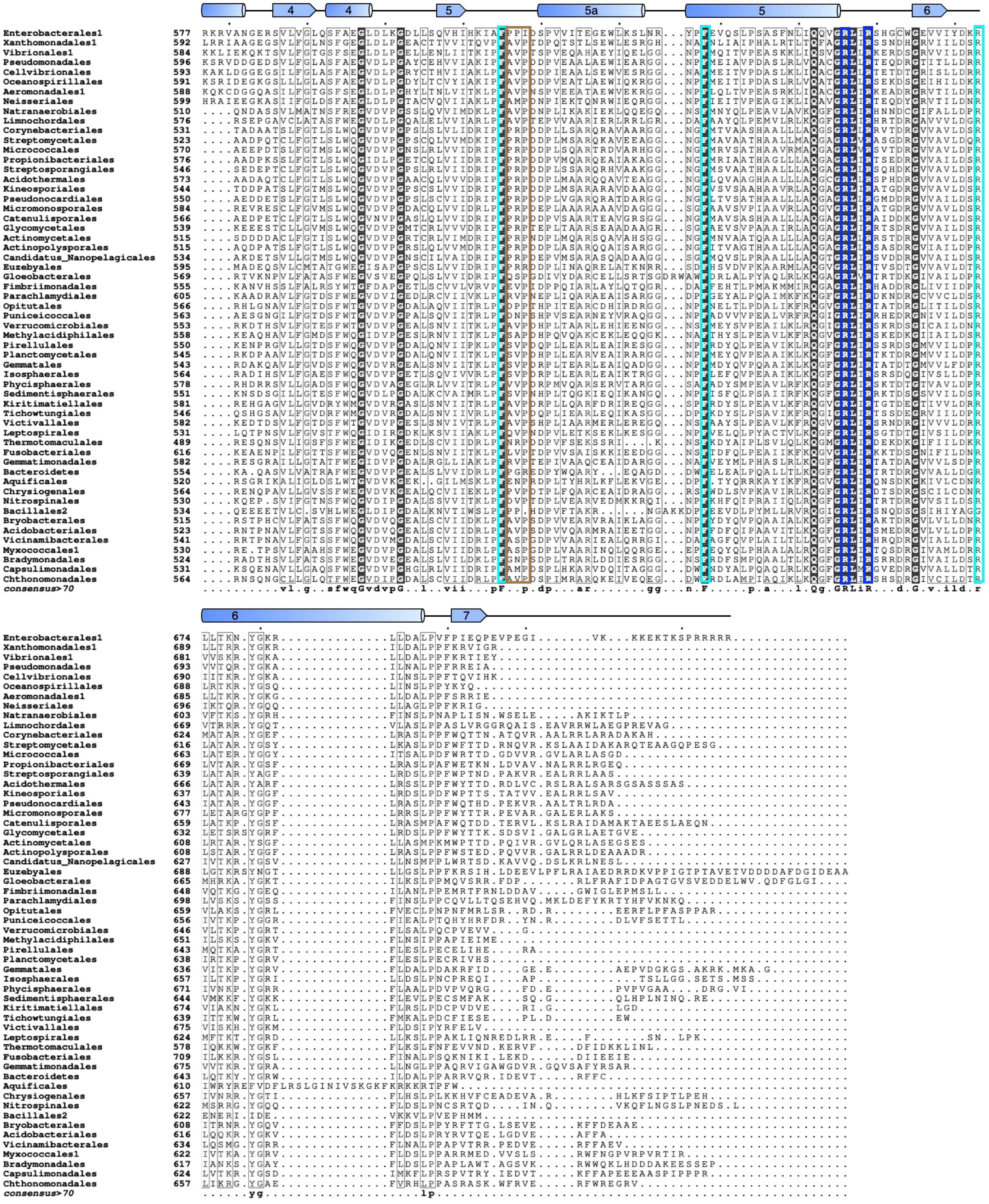
4
4
5
5a
5
6
6
7
