# Supplementary material for: Structure, function and evolution of the bacterial DinG-like proteins
Source: Comput Struct Biotechnol J. 2025 Mar 17;27:1124–39. doi: 10.1016/j.csbj.2025.03.023 (PMC11981726; doi:10.1016/j.csbj.2025.03.023)

Figure S10 Supplemental information for other subgroup of DinG-like proteins.

The structural models of the DinG-like–ssDNA complex from *Truepera radiovictrix* (The Trueperales order) (A), *Nakamurella multipartite* (The Nakamurellales order) (B), *Humisphaera borealis* (the Tepidisphaerales order) (C), *Caldilinea aerophila* (the Caldilineales order) (D), Ktedonobacterales bacterium SCAWS-G2 (the Ktedonobacterales order) (E), *Desulfotalea psychrophila* (the Desulfobacterales order) (F), *Hippea maritima* (the Desulfurellales order) (G), and *Kosmotoga olearia* (the Kosmotogales order) (H) in the presence of ATP·Mg^{2+} were predicted using AlphaFold 3.

The input parameters, including protein sequences, substrate information, and ligand specifications, are detailed in the corresponding figure. The model′s quality assessment are presented alongside the structural prediction.

A

Input	Copies	Sequence
DinG-like from *T. radiovictrix*	1	MLFPFKVPRKGQLEAIAAAREAFARGKRFVVIEAPTGSGKSGVAVTLAREASSAYLVTAQKLLQDQYARDFPELALMKGRANYR CLVAPTHAAAAPCIAGRKFPECDDCPYFCAKEAAMAASGTLLNYAYYLTELNYQGGFGPRELLVLDEAHNAEGALMSFVEVSF SDAQLRRVGIGEALPVVLDENEWFEVAEDLVPLFRSRRRELEAQLKGARLPTETALELLAHKGWLESQLARLELLAYSRDEENV EWVALRTSGSGGQSVTLKPVKVAAFAEELMFRFGERVLMLSATILDPPTYLRSLGIDPGDAEVITIASDFPPENRPIYPRPVARLTR HHLERDLPKLVHEIIELLEAHPEDKGVIHTHTYKIAAYIARYLPKRHAARLVTHDSAEGREAALDKHLRSRQPTVLLTPSMTEGID LPGDLSRWQVICKLPYPYLGDPQIARRRELDPAWYDWRTCLTVVQAYGRSVRSRDDFAVTYVLDADFGAFVRRQRARLPGWFL EAVQG
DNA	1	TTTTTTTTTTT
Ligand	1	ATP
Ion	1	Mg

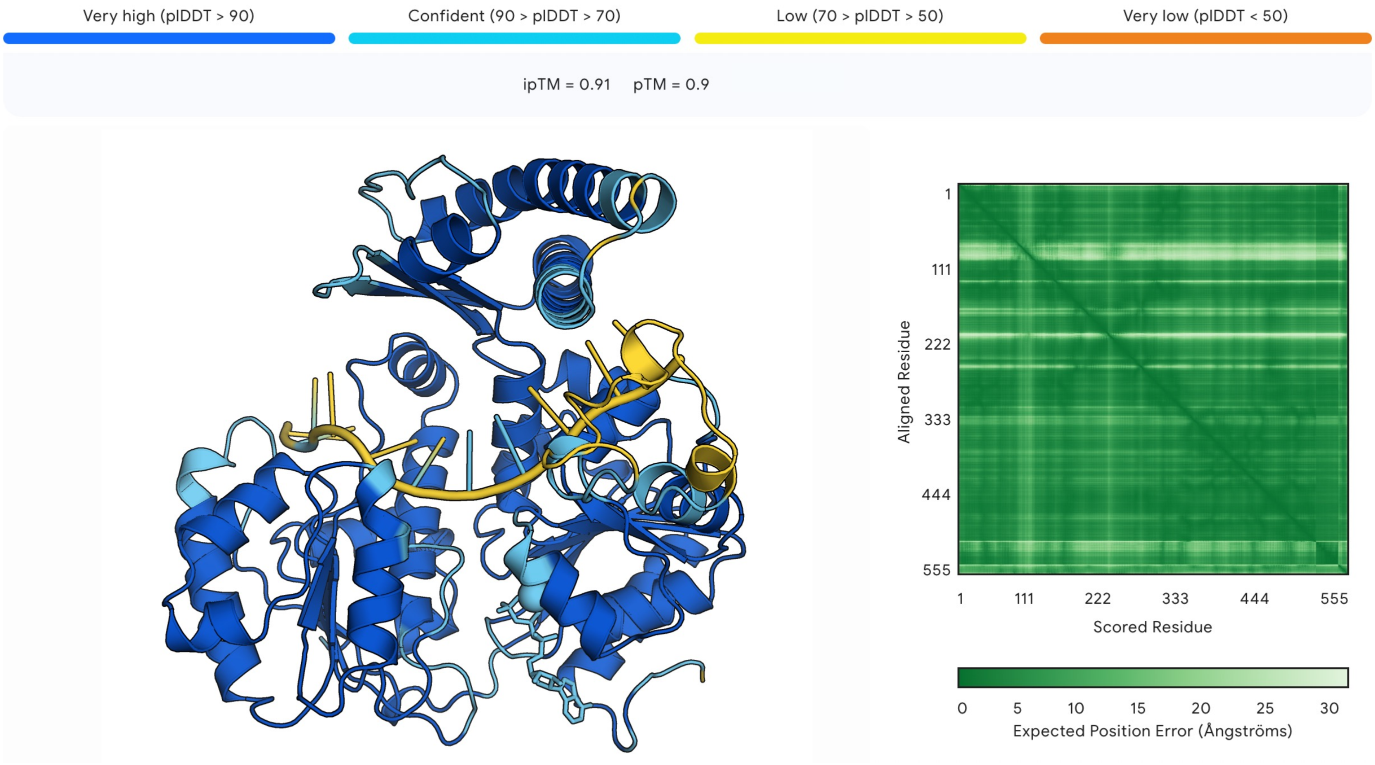

B

Input	Copies	Sequence
DinG-like from *N. multipartite*	1	MIDDEHRQVLDCAVELATGGAVFRPRAAQLDLSCRVLAAMEQTGQCAVEAPTGTGKTLAYLASAALRAARCDERTVISTESLSL<br>QQQIADKDAPVITEAVESVTGQSVSVAVYKGWQNTACAMSAVRVLQALLDDFHAPVPVTRDGLLDLADAAEEAVSGMPTRTRI<br>TVDGQLTDPRDALGPAAWALRASATVGPADRPSYPHPITERQWQSVSVSATDCLRNRCPLLEFCRPLAAKQRASTADLVVTNHT<br>LLGIQAAKKVPVVLSSRSLGRFHHIVVDEAHGLPDAVRKQGESTMSGRRLLNLVRDVEQGVDQWSDGSGKLDSDTAVALKARL<br>GGAGALASRLDVALTAAAGTAKEQVRLGDGVSPFGDAADDLVAFTTGVGGVIRALGLPEDGDTDRQRLHLRLVNRLSAFRSAV<br>DAVSTHRTGVARWLEQQEDRSWVVKASPAQVADALRHQVWVTEPVDRADQLAQQNRGTVDFDPDPADISASTGAADVAGSRV<br>VEPLSVCLVSATLPVGFAREVGVSARPAVLKSPLLAAFGASAVHVPTLTDTDLPALTERGPGGRVRLNTAEHPQWAARRITELVD<br>ANGGSALILVATARTGRLYADALRLAAKGRWRVMSQWDGRPAGATATLWREDHSAVLVGTRSFMTGLDAPGLTCTLVVLDRIPR<br>APQNPLGQARVEQLEADGLNRWQADRRVYAGDAAVLTHQAVGRLIRRETDIGMVAVLDPRLLKNKPWSYPEATRKLYAEALGD<br>FGFRTSRHDRAVEWLRELRAARAPKAG
DNA	1	TTTTTTTTTTT
Ligand	1	ATP
Ion	1	Mg

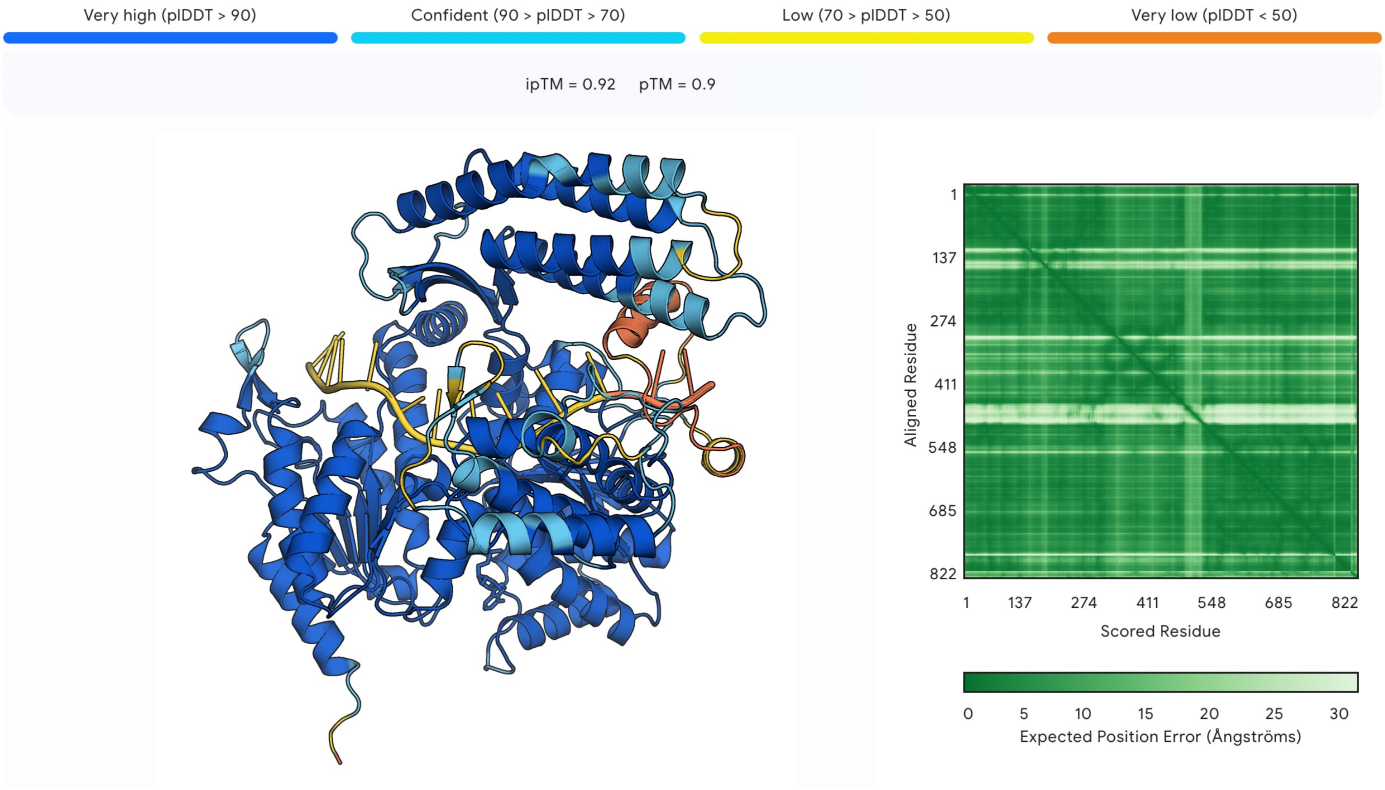

C

Input	Copies	Sequence
DinG-like from *H. borealis*	1	MPTTLHDILGPQGAIARRLGKSYEHRPQQLEMAAAVADALETGHHLVAEAGTGVGKSFAYLLPAIDFATRHKKKVVISTHTISLQ EQLIDKDIPLIRAVYPEEFTAVLVKGRSNYLCRRRLDQTRQRQAVMFDEERQVESLWQIEQWAATTTDGSLADLPALPYPGVWD KVCAEQGNCLGKKCRFYEGCHWQAAKRRMQGGTILVVNHALFFSDLALRAAGVQYLPKYDAVIFDEAHTLEDVAGSHFGLKV SESTINHQLRTLYDPRKGKGMLSTHGSSANPAIQDVVDLAHLVDDFFDRCVDWQKTQGRANGRINTPGFVDNDLSPKLNDLAM HLKAMLAEIKNEEELSELTSQSEKVATLASTVDALVNQSMEGAVYWMEQPATGAQVRGRAMTGQRRVTLNAAPVNVAEGLRM HLFEKLKSVVLTSATLSTGNGEIAITPRGVMPNGPVSDTGFQPVQGASRVEGLRIRQGAYLPHWNKDAATYAVNFRLADSLPKH VLEGWIAEREDILVNARQQHRPLTETERDRLRVLFSERVEAFLDQGHGECVLADESAAAVVAEALKHFDGQRYRLIAWCVMPN HVHVVVQPLGTHSLESILHSWKSYTANAINKSLLKSGTLWMAESYDHLIRDQADLDHAIKYTWENPEKAGWTDWEWRGIEKK AAVSEQSVASRTSHGLEARVTDGAIRPRSPSSGPDPFAYVRARLGLINERTLQVGSPFDYASQATLYLEENLPDPNDNHRFLPAAC DRILHYVRKTYGGAFVLFTSYRMLAEAAARLKPHFDSLGLPLLVHGQGAPRKVLLERFRSIDNAVLFGTSSFWQGIDVQGDKLR NVIITKLPFAVPDEPVIEARLDAIKRAGGNPFMEYSVPEAVIKLKQGFGRLIRSKTDTGIVVILDSRVKTKRYGKLFLDALPGCKTV TVK
DNA	1	TTTTTTTTTTT
Ligand	1	ATP
Ion	1	Mg

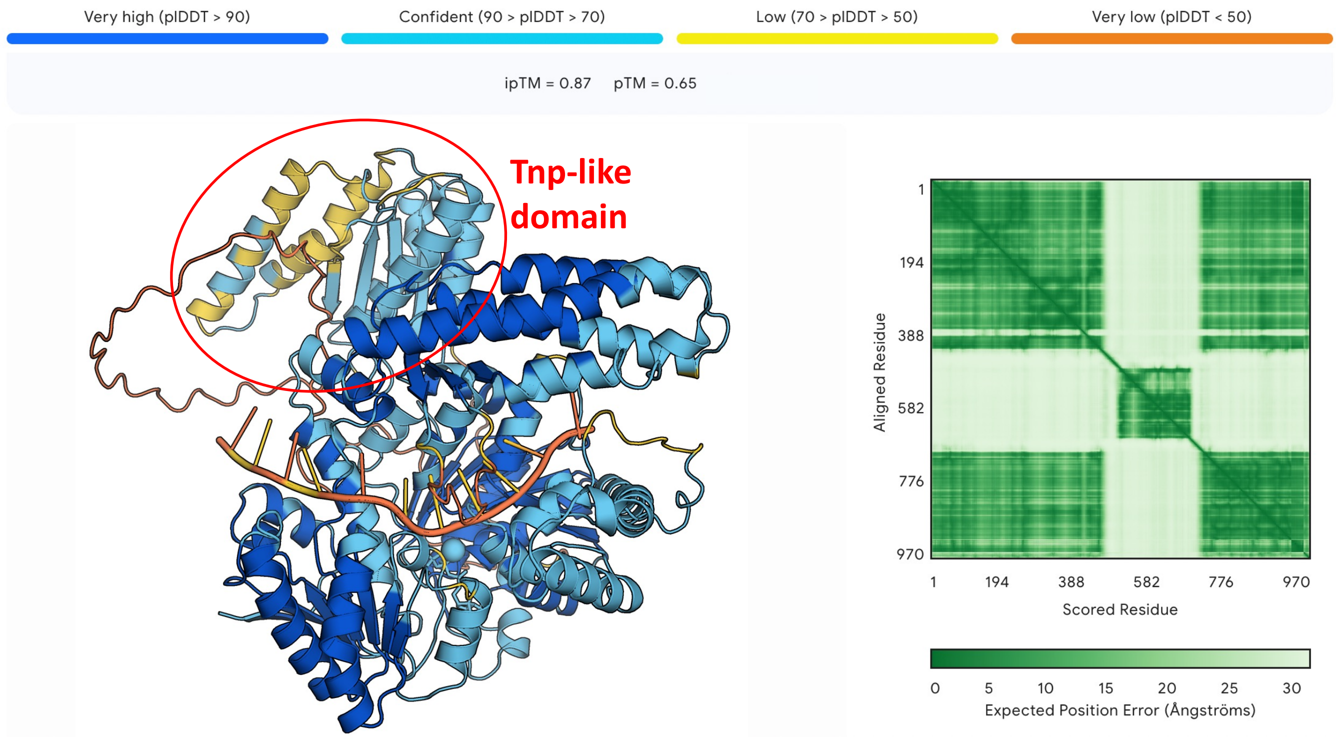

D

Input	Copies	Sequence
DinG-like from *C. aerophila*	1	MPRRSIFSATPAGTDPALHLPEERASVLDPEKEAPNKAEDILSALANTDLSEFFSSTGPLAQVLEGYELRHSQLQMAEAVKRAILS<br>RRHALIEAPTGTGKSIAYLIPAILSGKTVVVSTANKSLQSQLFQKEIPFLRKVLNRPISAVIVKGRSNFICTLKWEKESREQRYISLY<br>DRADEQFQFLQRWLDETESGDIDELPFVLNNDLRMRVVSFPDDCLHNDCRHYDDNCWVNRMRDEAAQAQVIITNHHLLLNAL<br>ELGYAGERILPPASIYVIDEAHHLEQIATAVFERMVTDYTVEQLLSRTIFKEHIGDDEIERLRYLNTLAFQEISQRSRENSFELTGDLE<br>GALSLSAALTELGQRLKRANPYAAAVEQAAKQGERPDPETAERHRSYELTVTALQSAAENLKAIGSSRKDGAVVRYAVRIFDRR<br>HVTLEVHAAPINPADLLSAYLFHPEDEDSELVDRTVICTSATLATAGGFHHYKARCGIRSVGEELVLPAVFDYPRQALLYQPALPA<br>YDYRNADAYYDAVAAEIERLLEVSRGRALCLFTSWSGLQQVSDRLQAGRSGATWPLRAQGDAPREALLSWFKATPYSVLLATR<br>SFWEGVDIPGEELSLVVLDKLPFPTPGDPLHAARMRAIDEQGRSSFEEYMIPLMTLALKQGFGRLIRRSDDCGVVAILDERLTSK<br>AYGRRSRQDLPPARFTREFRDVHRFYSQALGSPAEFALNVWPDSDAPQSWRWRLVRLSDGRAEMHSVRADVSTPEHAEIHAAL<br>NGLQDLQRRIRRAGQESSRYAVELRCSRKAAQWLTEAPDQDELFKRWVHAVSAWQAVLLRAVRE
DNA	1	TTTTTTTTTTT
Ligand	1	ATP
Ion	1	Mg

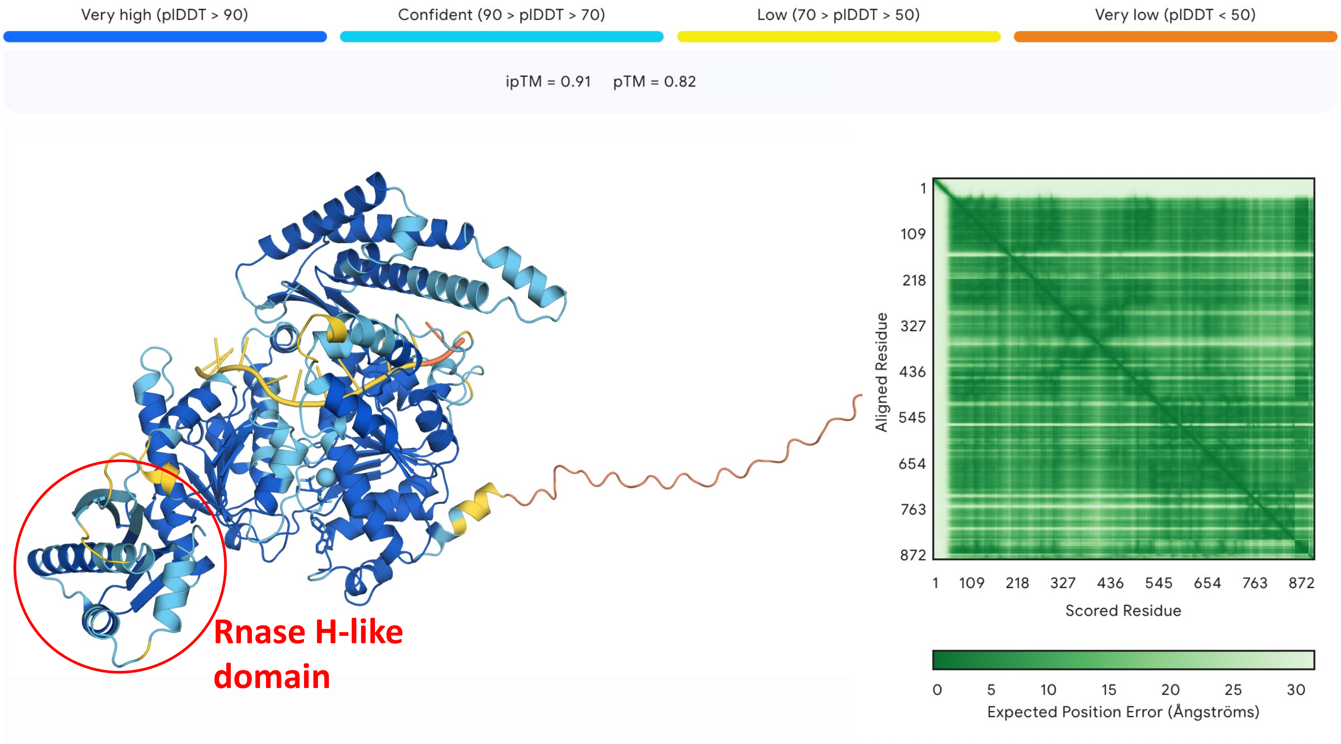

E

Input	Copies	Sequence
DinG-like from *Ktedonobacterales* bacterium SCAWS-G2	1	MAEVEPQQIGPYAITALLRKSSTSTFYRGKQRKKDLLIQWLDAPLTSSEARDAFLMRAKQLNKLKDRKIINTLDANFAGEHAYLV<br>MEYVQGQPLQELLTGAPHPAQEVRRYLSPIAEALQYAHVHHTLHGKLHPGSILVDERNNALLTDFTLTEPGTLPSLDEEARAVPY<br>MAPEHLDGQPTAASDQYSLAVIVYELLCGRRPYDATQRTQLRQQQEQLALPELHAFNAELSPELEQVIRQALSFKPEERFPHVQA<br>FADNYLHVLMGIPLKPVKVASTRSRSATRQSPEKPTNGLAALPSEKLSKPEESVKRPRGTLPDKEALRLSVLSPEKDQEEAERVA<br>AKPLPRKSTLTLVPERSAREDSQVLTAESGPGSQASRNGQETPPAKAQPSASPSSARLQAMVVADLGQGGILSRSLPGYEERSAQI<br>EMATLVARSLTQNVPAITEASTGTGKSLAYLVPVVRSGKVAIVSTANKALQEQLFYKDIPFVQRYIKPFEAALVKGVNNYICIDRL<br>EDERVGMQFYAKNVEFQRLLRTVADPDSGFNGDFETLGFQLPKDVRGRIATDSDQCAWSKCNFFSQCYVRLMREQAQMAQVI<br>VVNHTLLLLDAALDGFLLPERDVVILDEAHHLEEEATRSFTITISPTQIQTLLAQRMLKDHSTPSLQDEVQRIAQNTWLRLEQIAD<br>LSYKGRTNLEEPLEEGLRLSSAIADLADSLRKQRPKDLPEKEGQLYDKLLKRTQNLSENLRVVFSVSQTNKFVYFVDRIEGAGAR<br>GGFTLQASAAPLDVTNWLKERLFNKCNVICTSATLATIGPDPANPTEKGPNFAYFRSRIGLDPAERNDVIERILPLTFDYEKNALLY<br>LPRHLPAPTYGSGSEGYMKAIAREMYSLVKASRGRAFLLFSSRRMLEYAYALMAPHLSDFPLLKQGEMTRLELTRRFREEKGSIL<br>FGLKSFWEGVDIAGEALSLVVIDKLPFDPPDDPVHEARVALMKAAGENWFGTYVLPQAVLRLKQGLGRLLRTREDRGVMAILD<br>TRLHTKGYGKLVLNALPPAQRTTSLKDVEAFFAEDHP
DNA	1	TTTTTTTTTTT
Ligand	1	ATP
Ion	1	Mg

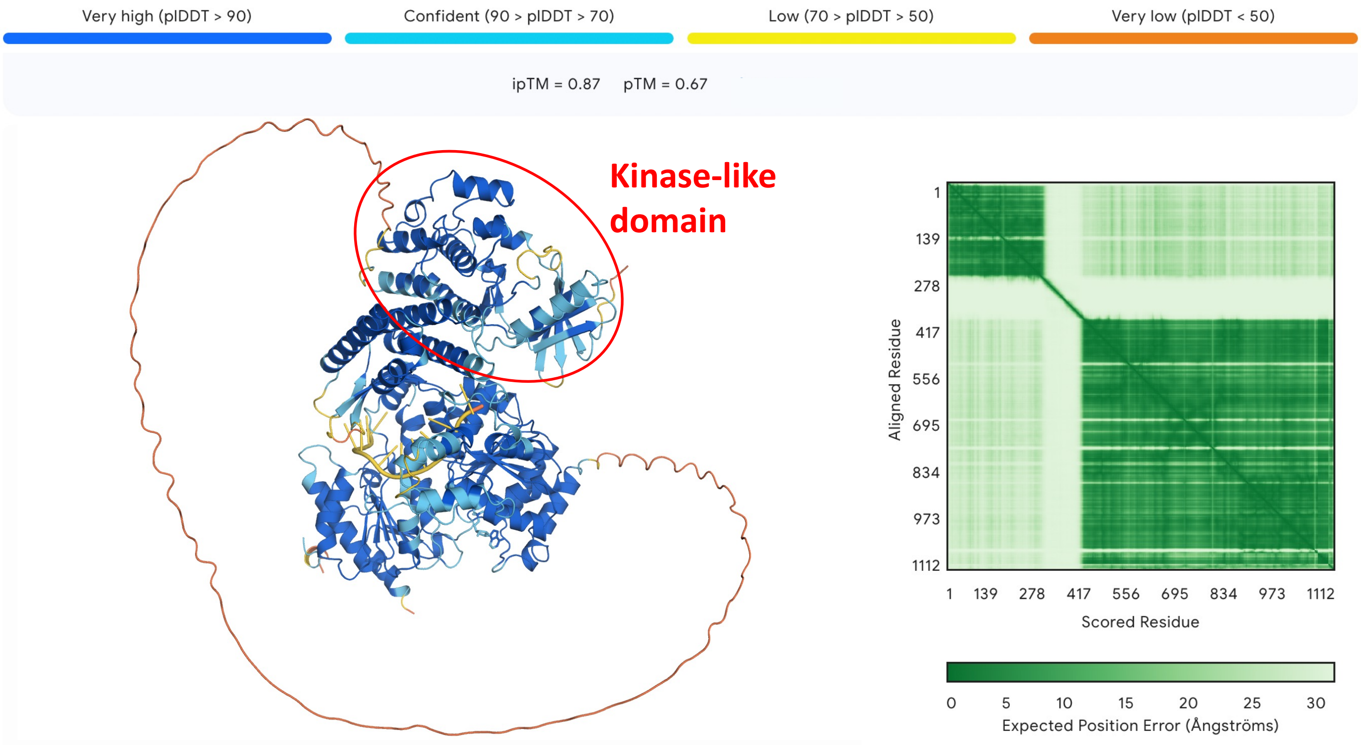

F

Input	Copies	Sequence
DinG-like from *D. psychrophila*	1	MLEKHMLIAKNYTIFLPNVTNILLANKFPCIIPLLFRLFPFLVSAASLLVSNISSPIPLLCTYFSSVHCFFSYLASSISYLVFLISYLLFR<br>PFPFSCILPLWPRRGRGRGKIWQDGERALEDFFGENGRLAGLIDHYQPRSGQQEMAQAVSRSLMDSNDPDPHAVSAPPRVLVVE<br>AETGIGKTLAYLLPAVISGKRVVVSTATRNLQDQIIHKEIPLLEKMFGGRVSAQCVKGRQNYLCLYKWYQHRSSAQLSLIAQDDE<br>DKIEQWLASTVTGDRAELHWLADDASLWHKISSQSDQCLGSECPEQENCFISRLRRRAAAARILIVNHHLFFSDLALKKEGYGEI<br>LPRYQAVIFDEAHHLEDIATTFFAKSFSSYQLRDILSDAERLGDKILVSDEHKNLLSRLSGMRVRLNAFMHVFPKKRGKTALKELV<br>TEYGQEAWQQEVELLATGIAKLIVALTDLHFKGEGWQTLVRRCQECHDNLRLTGLARDTTSSNYVHWFEHREKSIVLSVTPISVA<br>KELNEFLYAGVESTIMTSATLSIGEKFDYLRERLGLPADTKYLRFASPFAYKEQALLYIPEGGFPETNAPDYGQKSCERILQILEQS<br>RGRALILFTSFSAMERAATWLTDKIDYRMLVQGRHSRKHLLEEFKADRDSVLLAVASFWEGIDVAGEALSCVIIDKLPFEVPTDP<br>VIQARMEYIKAAGGNPFMDFQVPRAVLTLRQGVGRLMRSDRDRGLITILDIRLFSKFYGKRFLRSLPPAPVTRSLDEVKKFFSTE<br>KTL
DNA	1	TTTTTTTTTTT
Ligand	1	ATP
Ion	1	Mg

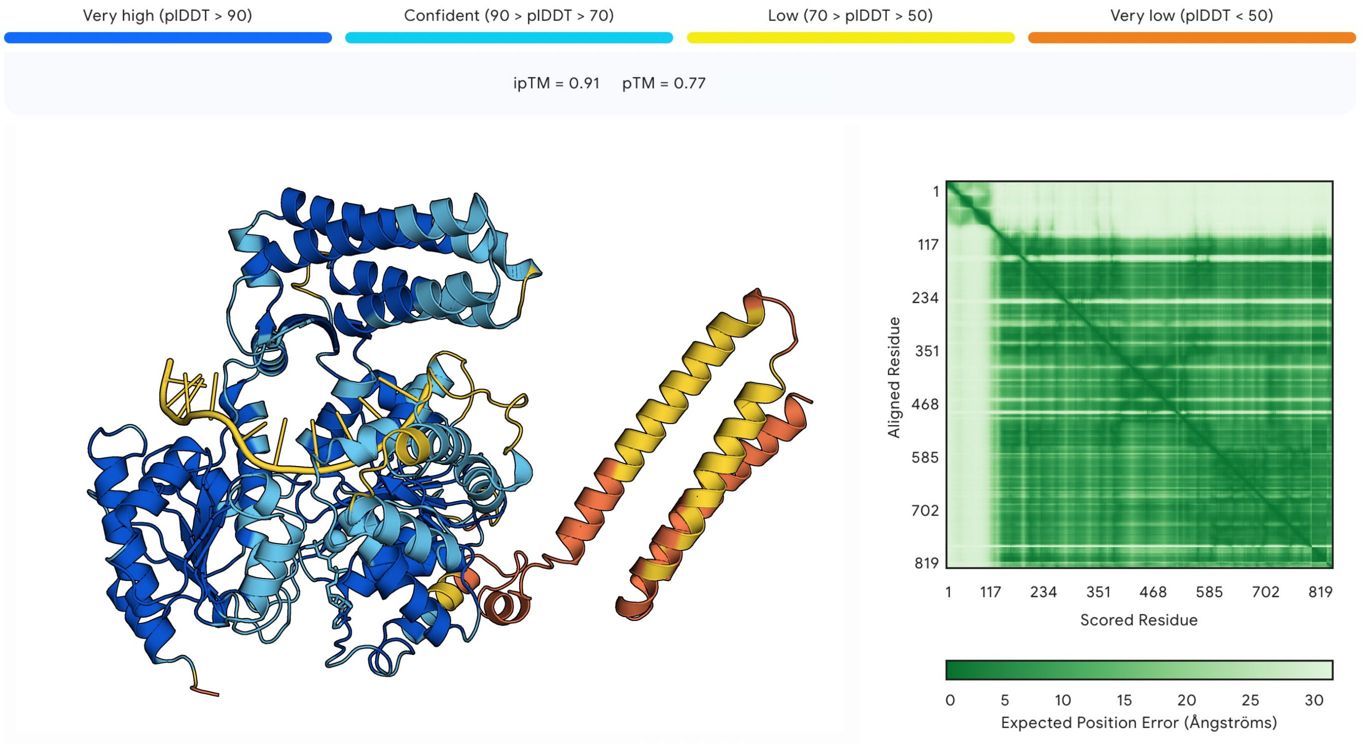

G

Input	Copies	Sequence
DinG-like from *H. maritima*	1	MRISPKSLDLLKKAGPSSLFKVRFNSEIIEIISNANSPSGDLLMVCEDRFEAVKFVATHSLSVGWFFWFNPLDSSLEAIAKPKIDIDG<br>IFESLFSRDGFEERKEQKQIAKIIFDSLKKSKNAIIEAPTGTGKSLAYLVACVIFSKQKGERVVISTNTINLQHQLVERDIPLLQEIVEF<br>RAVLALGRSNYICKRRVEDILTKGNVFLFENDLYKKIKEFLLNTKTGLKSEFFSIYENVPEDVWRNVESSTLLCAHSKCPYYKNS<br>CFFYKARAELEKADVIVANHHLVLSDSILESARILPDAYAVIFDEAHNIERNATNYYTISVSSDDILRSIDALYTKRKSKAYGLLSN<br>VEGYKNLKELLVNFRVELESTFDGLIQQFVAEQLNIDDSNIKLIYKPVSKILELLNSIILNLKGFLDENKDKDFVDIRGVTSFLSGC<br>ADNLGTFLKLNDGFVCWIKRFKKTMHFNITPLDVRSALKGHLYDKLASVIFISATLSVGGELEFFKRSVGVDNAVEFIAESNFDY<br>DKLARLLVVEDVKEPTQKGFDEDAADVILSIAESLKNTNKGVLVLFTSYAMLSSIYKRVYTELKQKGFNTFRQGELDNFELLIRF<br>KKGKGFLFATSSFWEGIDVKGEQLSVVVMVRLPFEVPTTPIEKTRYELLKKQGYNAFLEYALPKAVLKFKQGLGRLIRKADDYG<br>VMVVLDSRLINKSYGRIFLNSVSYIKSKRVRKNEIKDFISDFFANFAL
DNA	1	TTTTTTTTTTT
Ligand	1	ATP
Ion	1	Mg

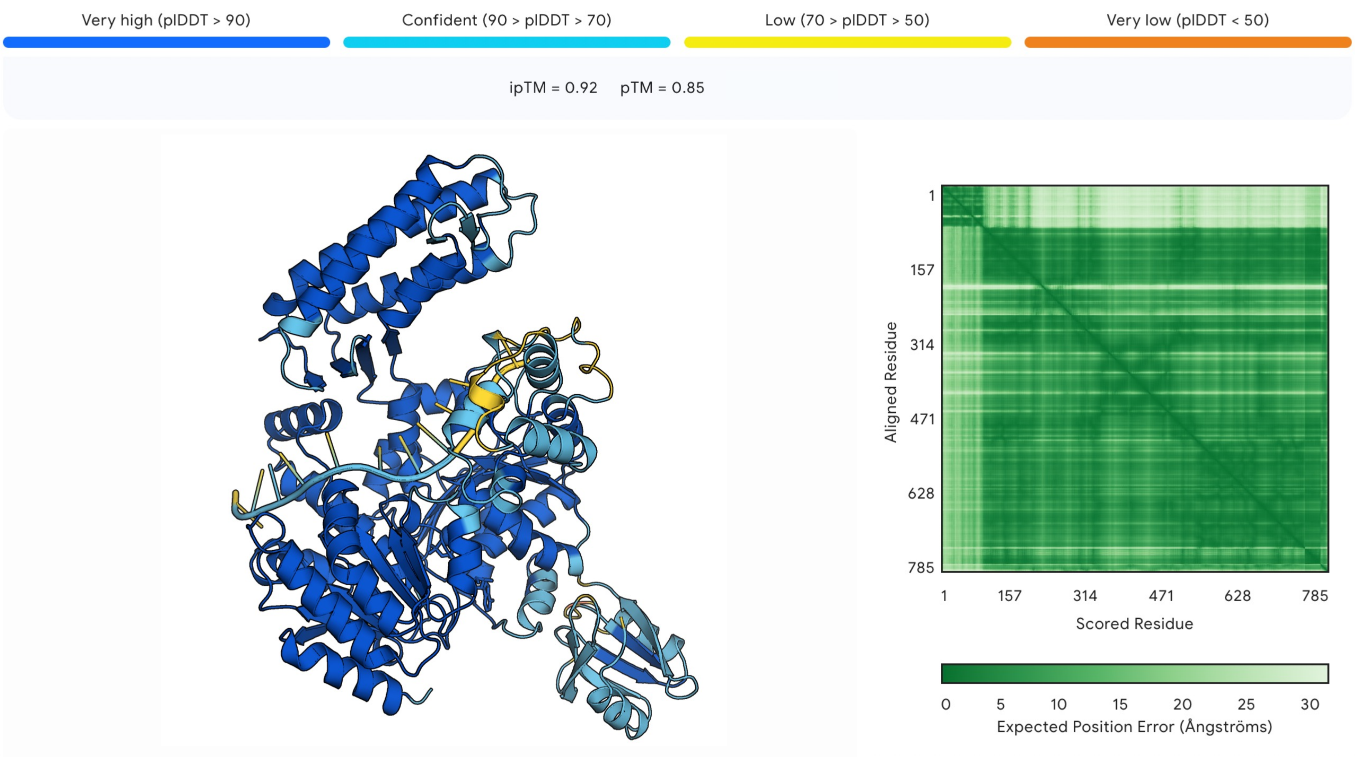

H

Input	Copies	Sequence
DinG-like from *K. olearia*	1	MTPLSGSEKSFSVNFKELKEFLGKHPVCLFPLNDETYKLLSNYPEVIDENFLKRFLVYPTGKESDKHINEYLQLLSSFPASLTDILP HFFEKNDGLGKFLQMGLSSGTKDSRWEDEVCSNLTKLKSVDHKGADIDLIKTVRMVFSEGGLLEDILEGYEYRQEQFMTALEIA ESIEGKQGIMIEAGTGTGKSLAYLIPSAYYSISKGEQIVVSTRTRMLQDQLARKDVAIVKKLPNLEELRVWTLKGRERYFCLKKYF EELEYAVGSGKSKNRTELFGVLLWSIKTGSGDLDELHLKEENRNRFTATRFECLRKLCPFFNRCPYYNSRDNAQNADIVITNHSL LFSEAHIRLEDSREGEDEPIGMLLPKFKVLIVDEAHELEMSLTEAMSFNLVPHEAVSTIRKAINCSKDALRSIRNHFERSFLEELWG RLRKFTAEIEKILKTIVVSYNNCSTDEKHSIDNEELEALKKDIGELYTIIQRFRAVLIQLLRMVEEVAEEEETPVLEKLNMELKSVTS ELDGLIRQLLGLSREEDGRVVYVRRTVTNNGNHLIITSAPIKNDQLMAAIFPDVPVKVFISATLWVYSGRSDGFNYARRILGLNES FHAIKLGSSFDFQEQLKFYIVKDMAKYQPNNREYLNQGANLISEMLKIVKGSAMVLFTSYKDMYYVISKISEELLDIRLQIQEPN NSPTAIVNEHVNSENSVIFGVRSFWEGIDLPGEHLKLLIIFKLPFERPDDPLINARIKHYGTKNYVEGLNKYYYPKMITAFRQGIGR LIRTRNDRGVLVVLDNRIVDGNKIYSRKLLRSLSPDVKINVIDGSRVISELRKLRRTKWF
DNA	1	TTTTTTTTTTT
Ligand	1	ATP
Ion	1	Mg

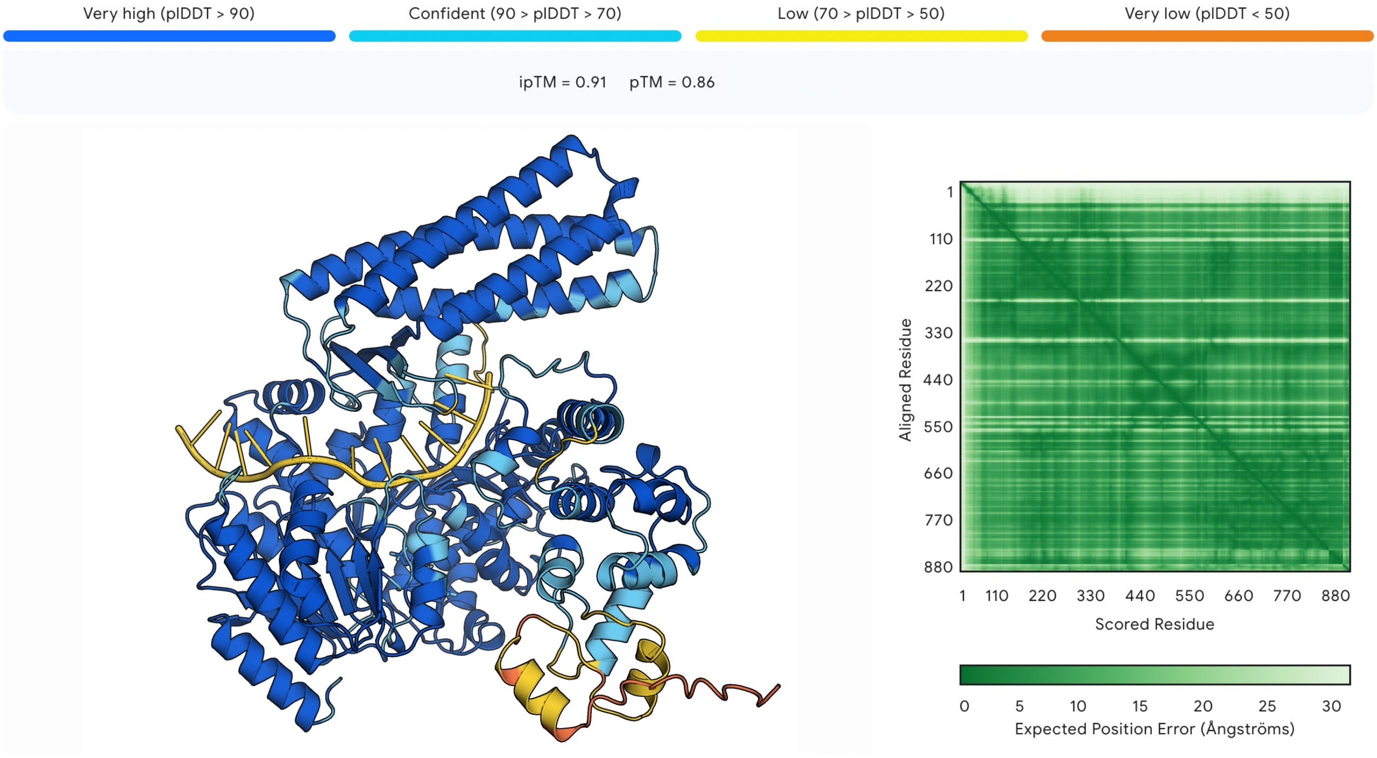

Supplement: Figure S10 — Supplementary material [file mmc10.pdf]
