# Supplementary material for: Structure, function and evolution of the bacterial DinG-like proteins
Source: Comput Struct Biotechnol J. 2025 Mar 17;27:1124–39. doi: 10.1016/j.csbj.2025.03.023 (PMC11981726; doi:10.1016/j.csbj.2025.03.023)
Supplement: Table S2 — Supplementary material [file mmc12.docx]

**Supplemental Table 2**

| *Input structure | Top output structures | PDB ID | Z-score | Rmsd  (Å) | Identity  (%) |
| --- | --- | --- | --- | --- | --- |
| The HNH domain of *Su*CasDinG-HNH | the HNH domain of IscB | 8CSZ | 4.7 | 3.1 | 23 |
|  | the HNH domain of *E. coli* McrA (EcoKMcrA) | 6GHC | 6.4 | 2.9 | 29 |
|  | the HNH domain of Cas5e from *Candidatus Cloacimonetes bacterium* ADurb.Bin088 | 8YB6 | 5.7 | 3.1 | 24 |
| The exonuclease domain of *Bs*ExoDinG | the NTD of *E. coli* Exonuclease I | 4JS4 | 20.7 | 3.0 | 18 |
|  | *E. coli* RNase T | 3V9X | 19.5 | 2.6 | 23 |
|  | *E. coli* Cap18 | 7T2S | 19.1 | 2.6 | 19 |
|  | the ε subunit of *E. coli* DNA polymerase III, DnaQ | 5M1S | 18.2 | 2.9 | 26 |
| The pseudo-exonuclease domain of *Cv*pExoDinG | the 3’-5’ exonuclease domain of polI | 1BGX | 9.1 | 3.2 | 18 |
|  | NanoRNase C | 7MPO | 9.3 | 3.6 | 14 |
|  | Nibbler | 7JW6 | 7.9 | 3.0 | 15 |
| The endonuclease domain of *Bb*ExoDinG | *E. coli* RecE | 3H4R | 9.7 | 2.7 | 15 |
|  | human exonuclease V (EXO5) | 7LW8 | 9.6 | 3.1 | 14 |
|  | CRISPR-associated exo/endonuclease Cas4 | 7MI4 | 9.4 | 3.3 | 17 |
|  | *B. subtilis* AddA | 4CEJ | 9.0 | 2.5 | 15 |
|  | Mouse Dna2 | 5EAN | 6.0 | 3.4 | 15 |
| The RadC-like domain of *Ds*RadC-like DinG | *Pyrococcus furiosus* JAMM/MPN^+^ metalloprotease | 5LDA | 10.2 | 2.5 | 20 |
|  | *Caldiarchaeum subterraneum* Rpn11 | 6FJU | 9.5 | 2.2 | 17 |
| The transposase-like domain in *Humisphaera borealis* DinG-like protein | *E. coli* MG1655 TnpA | 4ER8 | 10.5 | 2.5 | 21 |
| The RNase H-like domain in *Caldilinea aerophile* DinG-like protein | *Thermus thermophilus* HB27 argonaute protein | 5XOU | 7.1 | 2.8 | 19 |
| the kinase-like domain in *Ktedonobacterales bacterium* SCAWS-G2 DinG-like protein | *M. tuberculosis* Ser/Thr kinase PknA | 4X3F | 27.6 | 2.7 | 25 |

* For each subgroup, models were initially predicted using AlphaFold 3 software, and the relevant domains were subsequently extracted as input structures for DALI analysis.
