# Supplementary material for: Structure, function and evolution of the bacterial DinG-like proteins
Source: Comput Struct Biotechnol J. 2025 Mar 17;27:1124–39. doi: 10.1016/j.csbj.2025.03.023 (PMC11981726; doi:10.1016/j.csbj.2025.03.023)
Supplement: Figure S2 — Supplementary material [file mmc2.pdf]

Figure S2 Supplemental information for YoaA subgroup proteins.

A. The structural model of the *E. coli* YoaA–ssDNA complex in the presence of ATP·Mg^{2+} was predicted using AlphaFold 3. The input parameters, including protein sequences, substrate information, and ligand specifications, are detailed in the corresponding figure. The model′s quality assessment metrics are presented alongside the structural prediction.

B. The structural model of the *E. coli* YoaA–HolC–SSB-Ct complex in the presence of ATP·Mg^{2+} was predicted using AlphaFold 3.

C. Multiple sequence alignment of YoaAs was performed using Clustal Omega and visualized by ESPript. The names of corresponding bacteria species, protein IDs, and protein sequences were provided in Table S1. Secondary structural elements were depicted based on the AlphaFold 3 predicted EcYoaA–ssDNA complex structure, displayed at the top of the sequences, numbered, and colored according to domain arrangement. Critical residues for metal coordination, ATP binding, DNA binding, and the P motif were highlighted in red, blue, cyan and brown boxes, respectively.

A

Input	Co pies	Sequence
*Ec*YoaA	1	MTDDFAPDGQLAKAIPGFKPREPQRQMAVAVTQAIEKGQPLVVEAGTGTGKTYAYLAPALRAKKKVIISTGSKALQDQLYSRDL PTVSKALKYTGNVALLKGRSNYLCLERLEQQALAGGDLPVQILSDVILLRSWSNQTVDGDISTCVSVAEDSQAWPLVTSTNDNC LGSDCPMYKDCFVVKARKKAMDADVVVVNHHLFLADMVVKESGFGELIPEADVMIFDEAHQLPDIASQYFGQSLSSRQLLDL AKDITIAYRTELKDTQQLQKCADRLAQSAQDFRLQLGEPGYRGNLRELLANPQIQRAFLLLDDTLELCYDVAKLSLGRSALLDA AFERATLYRTRLKRLKEINQPGYSYWYECTSRHFTLALTPLSVADKFKELMAQKPGSWIFTSATLSVNDDLHHFTSRLGIEQAES LLLPSPFDYSRQALLCVLRNLPQTNQPGSARQLAAMLRPIIEANNGRCFMLCTSHAMMRDLAEQFRATMTLPVLLQGETSKGQ LLQQFVSAGNALLVATSSFWEGVDVRGDTLSLVIIDKLPFTSPDDPLLKARMEDCRLRGGDPFDEVQLPDAVITLKQGVGRLIRD ADDRGVLVICDNRLVMRPYGATFLASLPPAPRTRDIARAVRFLAIPSSR
DNA	1	TTTTTTTTTTT
Ligand	1	ATP
Ion	1	Mg

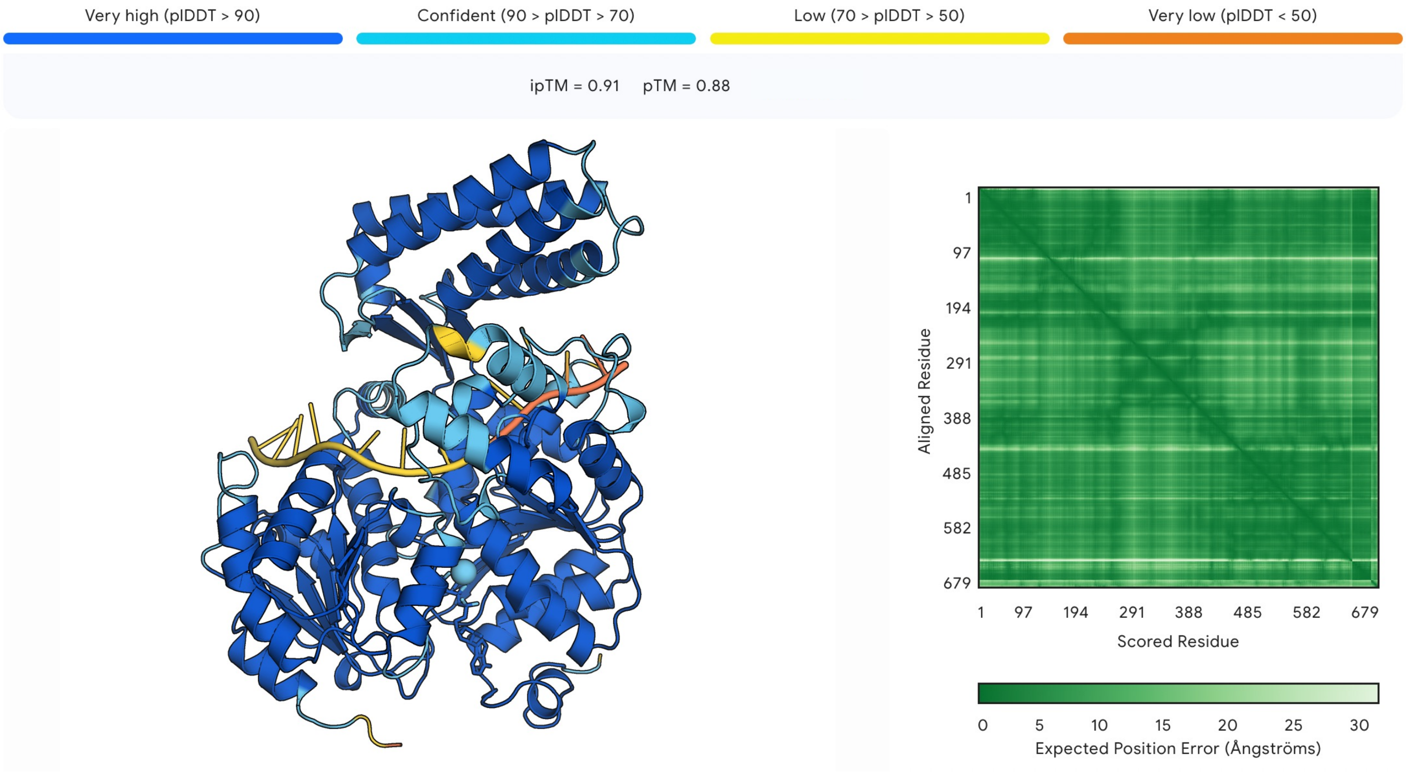

B

Input	Copies	Sequence
*Ec*YoaA	1	MTDDFAPDGQLAKAIPGFKPREPQRQMAVAVTQAIEKGQPLVVEAGTGTGKTYAYLAPALRAKKKVIISTGSKALQDQLYS RDLPTVSKALKYTGNVALLKGRSNYLCLERLEQQALAGGDLPVQILSDVILLRSWSNQTVDGDISTCVSVAEDSQAWPLVT STNDNCLGSDCPMYKDCFVVKARKKAMDADVVVVNHHLFLADMVVKESGFGELIPEADVMIFDEAHQLPDIASQYFGQ SLSSRQLLDLAKDITIAYRTELKDTQQLQKCADRLAQSAQDFRLQLGEPGYRGNLRELLANPQIQRAFLLLDDTLELCYDV AKLSLGRSALLDAAFERATLYRTRLKRLKEINQPGYSYWYECTSRHFTLALTPLSVADKFKELMAQKPGSWIFTSATLSVN DDLHHFTSRLGIEQAESLLLPSPFDYSRQALLCVLRNLPQTNQPGSARQLAAMLRPIIEANNGRCFMLCTSHAMMRDLAE QFRATMTLPVLLQGETSKGQLLQQFVSAGNALLVATSSFWEGVDVRGDTLSLVIIDKLPFTSPDDPLLKARMEDCRLRGGD PFDEVQLPDAVITLKQGVGRLIRDADDRGVLVICDNRLVMRPYGATFLASLPPAPRTRDIARAVRFLAIPSSR
DNA	1	TTTTTTTTTTT
Ligand	1	ATP
Ion	1	Mg
EcSSB-Ct	1	DDDIPF
EcHolC	1	MKNATFYLLDNDTTVDGLSAVEQLVCEIAAERWRSGKRVLIACEDEKQAYRLDEALWARPAESFVPHNLAGEGPRGGAPV EIAWPQKRSSSRRDILISLRTSFADFATAFTEVVDFVPYEDSLKQLARERYKAYRVAGFNLNTATWK

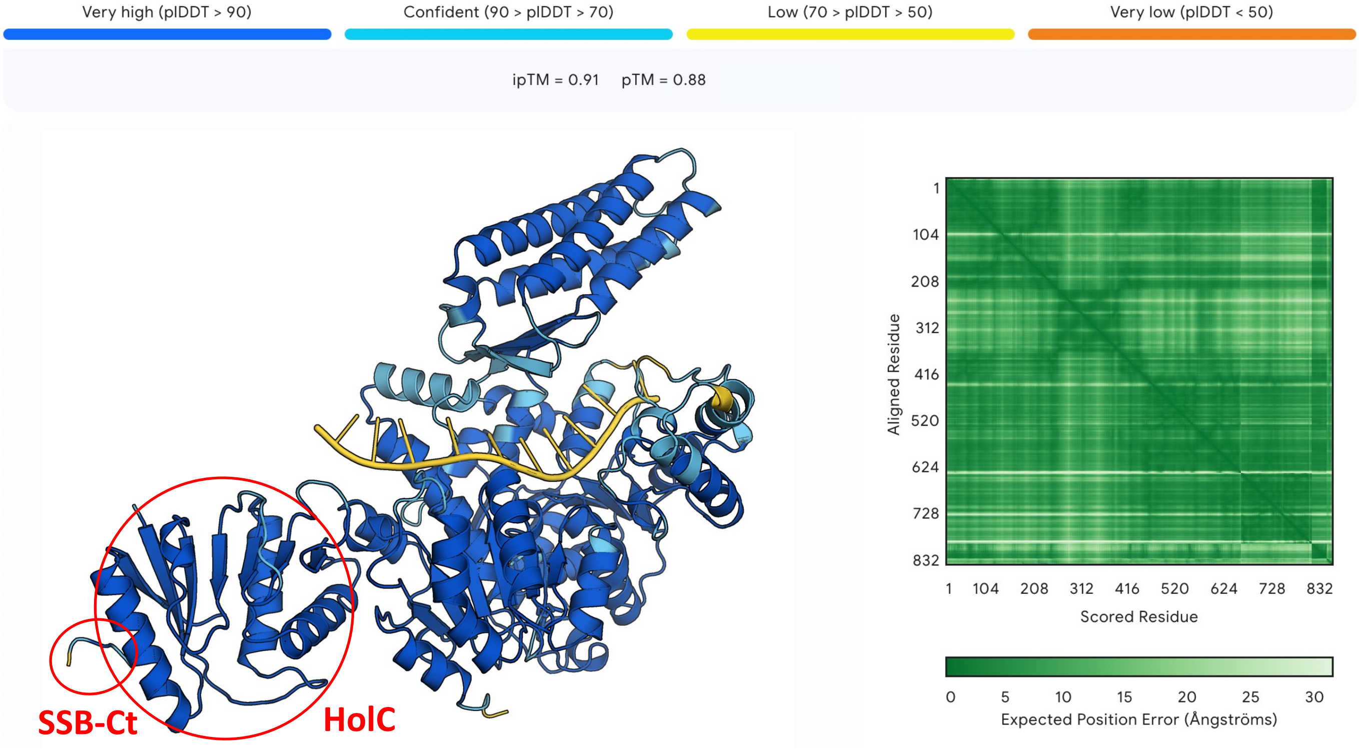

C

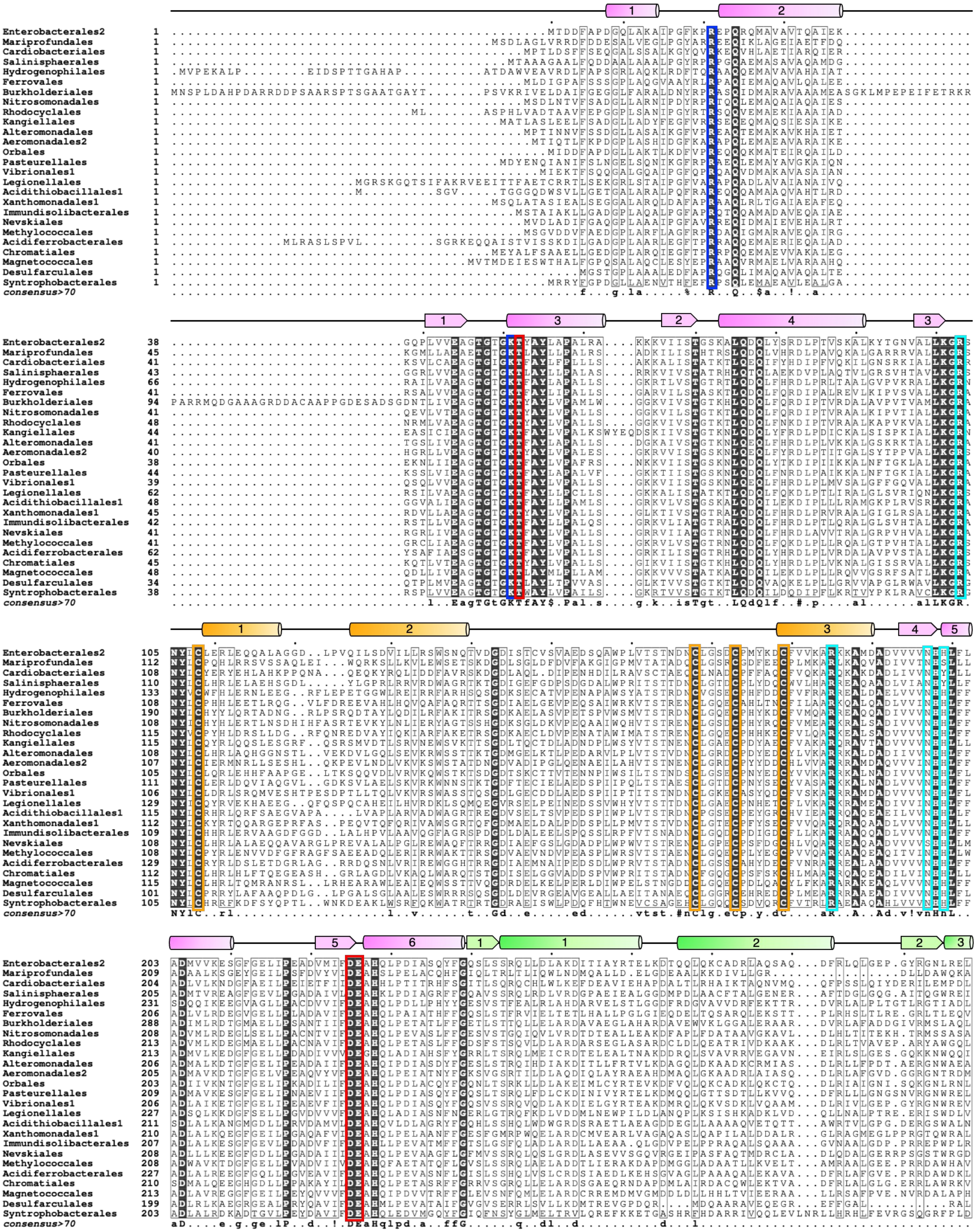
Enterobacterales2
Mariprofundales
Cardiobacteriales
Salinisphaerales
Hydrogenophilales
Ferrovales
Burkholderiales
Nitrosomonadales
Rhodocyclales
Kangiellales
Alteromonadales
Aeromonadales2
Orbales
Pasteurellales
Vibrionales1
Legionellales
Acidithiobacillales1
Xanthomonadales1
Immundisolibacterales
Nevskiales
Methylococcales
Acidiferrobacterales
Chromatiales
Magnetococcales
Desulfarculales
Syntrophobacterales
consensus>70

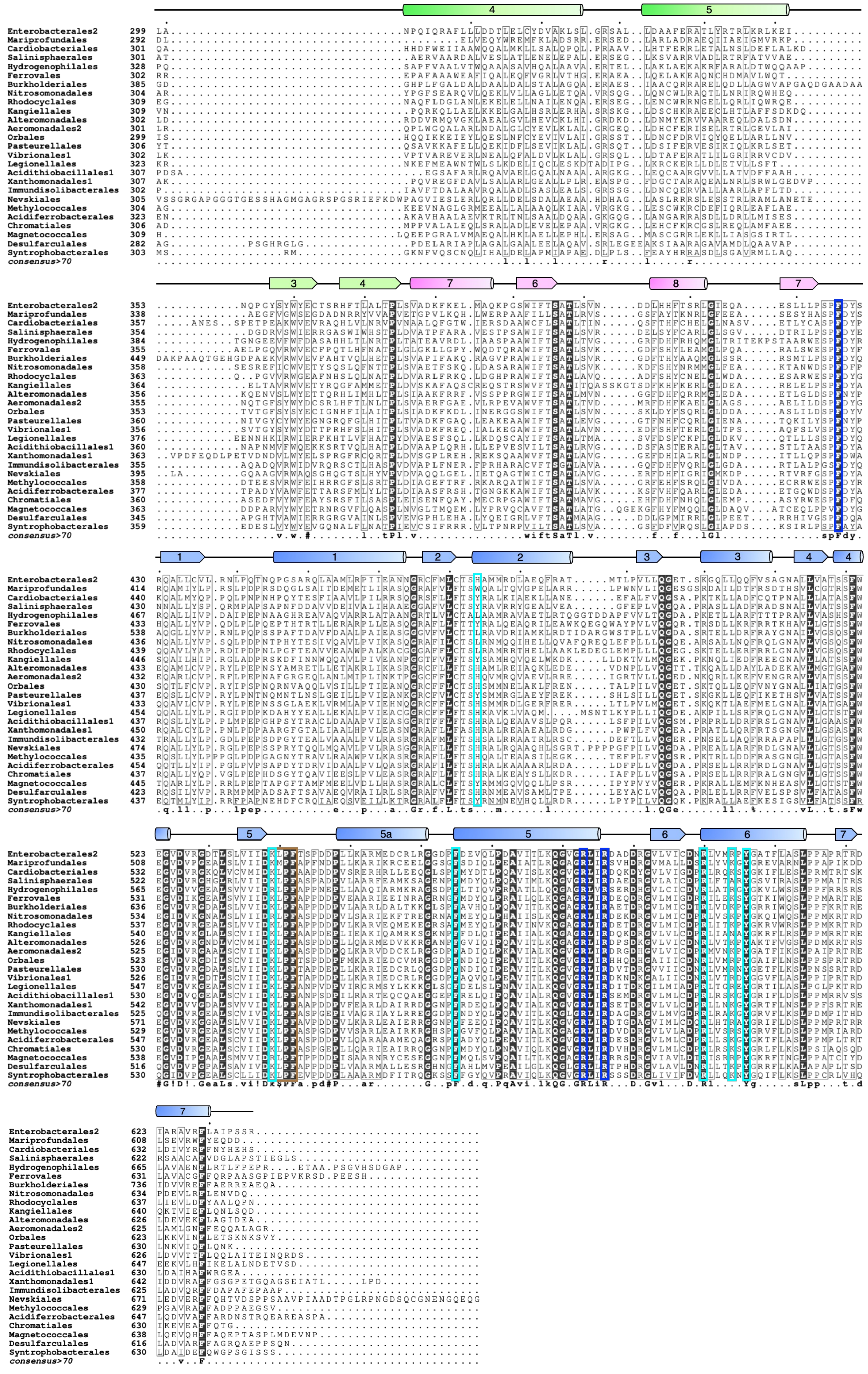
Enterobacterales2
Mariprofundales
Cardiobacteriales
Salinisphaerales
Hydrogenophilales
Ferrovales
Burkholderiales
Nitrosomonadales
Rhodocyclales
Kangiellales
Alteromonadales
Aeromonadales2
Orbales
Pasteurellales
Vibrionales1
Legionellales
Acidithiobacillales1
Xanthomonadales1
Immundisolibacterales
Nevskiales
Methylococcales
Acidiferrobacterales
Chromatiales
Magnetococcales
Desulfarculales
Syntrophobacterales
consensus>70
