# Supplementary material for: Structure, function and evolution of the bacterial DinG-like proteins
Source: Comput Struct Biotechnol J. 2025 Mar 17;27:1124–39. doi: 10.1016/j.csbj.2025.03.023 (PMC11981726; doi:10.1016/j.csbj.2025.03.023)

**Figure S3 Supplemental information for CasDinG subgroup proteins.**

A. The structural model of the NTD of PaCasDinG was predicted using AlphaFold 3. The input protein is detailed at the top. The model′s quality assessment metrics are presented alongside the structural prediction.

B. Multiple sequence alignment of CasDinGs was performed using Clustal Omega and visualized by ESPript. Pseudomonas *, CasDinG on the plasmid of *P. aeruginosa* PAO1 strain; Acidithiobacillales2, CasDinG of *Acidithiobacillus ferrooxidans* ATCC 23270. Protein IDs and protein sequences were provided in Table S1. Secondary structural elements were depicted based on the PaCasDinG–ssDNA complex (PDB ID: 7XF1) (the unsolved NTD was predicted by AlphaFold3), displayed at the top of the sequences, numbered, and colored according to domain arrangement. Critical residues for metal coordination, ATP binding, DNA binding, and the P motif were highlighted in red, blue, cyan and brown boxes, respectively.

**A**

| Input | Copies | Sequence |
|---|---|---|
| *Pa*CasDinG _NTD | 1 | MSPPTDQRSLWARTLLSEAVDQGLDSLPVPLQDVSTFTVTLQPALAVRLKALADQHNTPVSVYAAGLIEAMRRRSESGSV |

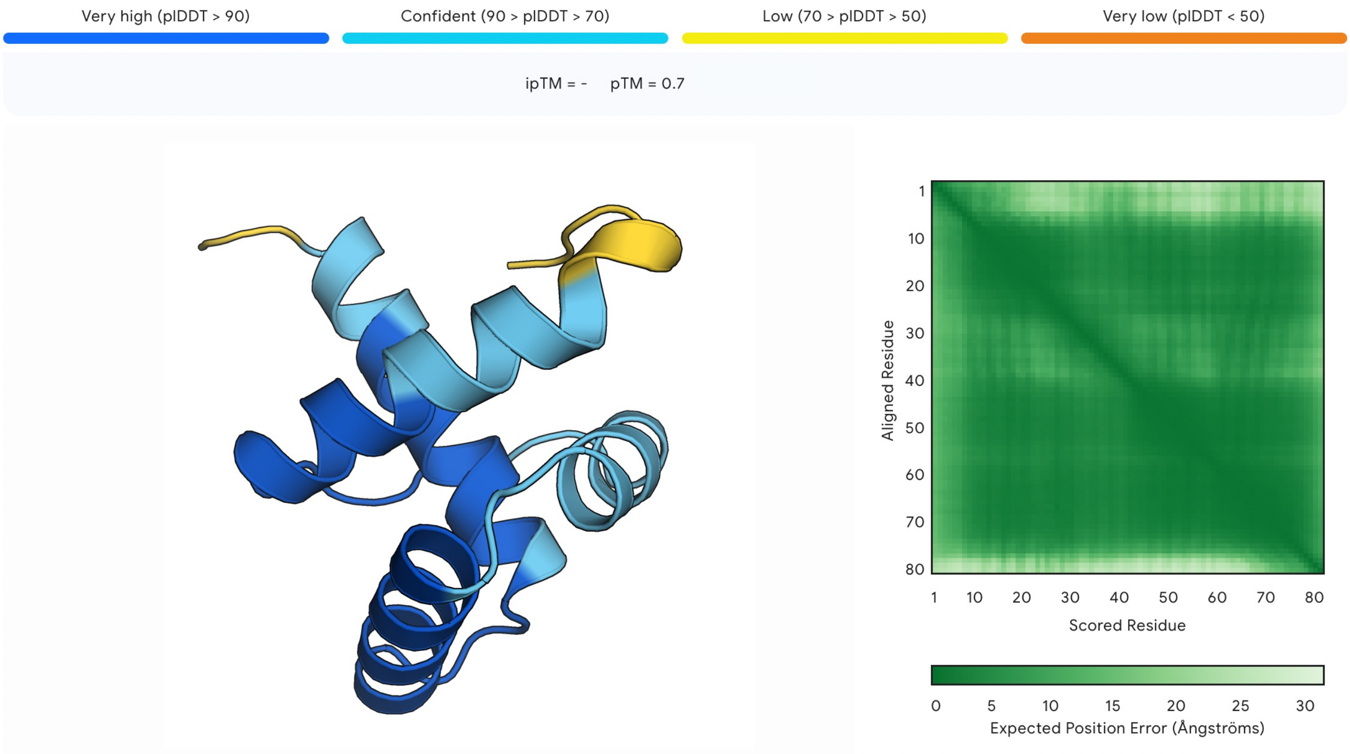

**B**

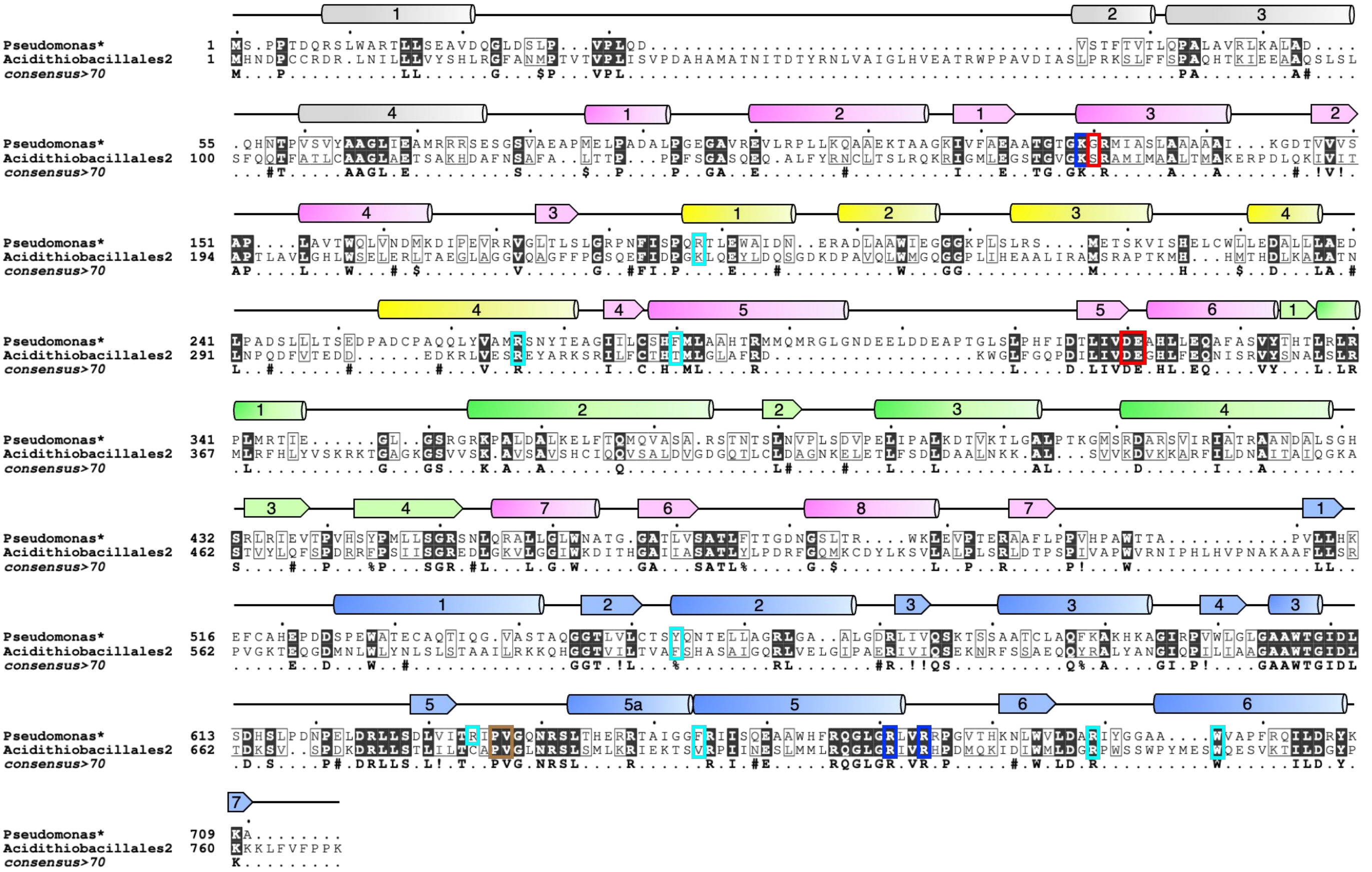

Supplement: Figure S3 — Supplementary material [file mmc3.pdf]
