# Supplementary material for: Structure, function and evolution of the bacterial DinG-like proteins
Source: Comput Struct Biotechnol J. 2025 Mar 17;27:1124–39. doi: 10.1016/j.csbj.2025.03.023 (PMC11981726; doi:10.1016/j.csbj.2025.03.023)
Supplement: Figure S4 — Supplementary material [file mmc4.pdf]

Figure S4 Supplemental information for CasDinG-HNH subgroup proteins.

A. The structural model of the SuCasDinG-HNH–ssDNA complex in the presence of ATP·Mg^{2+} and Zn^{2+} was predicted using AlphaFold 3. Based on the reported structure of the IscB-ωRNA-DNA complex (PDB ID: 8CSZ), the HNH domain is suggested to bind a DNA/RNA hybrid of approximately 12 base pairs, with its active center coordinating a Mg^{2+} ion. Additionally, its C-terminal region contains a Zn knuckle capable of binding a Zn^{2+} ion. Therefore, when predicting the structure of the CasDinG-HNH-DNA complex, the aforementioned cofactors, including the DNA/RNA hybrid, Mg^{2+} and Zn^{2+} ions, were also incorporated into the input. The input parameters, including protein sequences, substrate information, and ligand specifications, are detailed in the corresponding figure. The model′s quality assessment metrics are presented alongside the structural prediction. Despite specifying a 12 bp DNA/RNA hybrid as input, SuCasDinG-HNH failed to incorporate high-quality models for the DNA/RNA hybrid.

B. Multiple sequence alignment of CasDinG-HNHs was performed using Clustal Omega and visualized by ESPript. Rhodobacterales, CasDinG-HNH of *Sulfitobacter sp.* JL08; Hyphomicrobiales, CasDinG-HNH of *Nordella sp.* HKS 07. Protein IDs and protein sequences were provided in Table S1. Secondary structural elements were depicted based on the predicted SuCasDinG-HNH–ssDNA complex displayed at the top of the sequences, numbered, and colored according to domain arrangement. Critical residues for metal coordination, ATP binding, DNA binding, and the P motif were highlighted in red, blue, cyan and brown boxes, respectively.

C. The structural model of the NoCasDinG-HNH–ssDNA complex in the presence of ATP·Mg^{2+} was predicted using AlphaFold 3. The input parameters, including protein sequences, substrate information, and ligand specifications, are detailed in the corresponding figure. The model′s quality assessment metrics are presented alongside the structural prediction. Despite specifying Zn^{2+} as an input, NoCasDinG-HNH failed to incorporate high-quality models for that.

D. The overall structure of the AlphaFold3 predicted NoCasDinG-HNH–ssDNA–ATP complex. ssDNA, ATP and Mg^{2+} are colored red and green, respectively. The input information used for model prediction is detailed in the supplementary material, while the Zn^{2+}, which could not be modeled with high confidence, has been omitted from this figure for clarity.

E. A comparison of the HNH domains between SuCasDinG-HNH and NoCasDinG-HNH.

A

Input	Copies	Sequence
*Su*CasDinG-HNH	1	MFRSPEQAEFNQFVANHISNPDAPLLIEGATGLGKTRAYLAAVFQTDKRVAICLATNALIEQILNSSDLPWAQELAPDKTVAVFRS RRYFEDDREAYEAQREAAQIADILICTASSVIFDQRLSGSYNGVTNRDVIVFDEADQIPGLAALASDLSIDRKTLRDLNCAASTAL EVAGKLLSLPQLDSEIRAKAKIIAEIASEPEVWYKRVGMTEEGGVSVIHRLPGRLLKKISNRPSTIFISATLSINGQFNDFKRAMGIG DSSSLSRIIEPKHHGHLSFSFFTDDPVDSDEWLATVVEQIEASDTPVLVATPSHKLATELGDRISGSTVRQRDETMTDAVGRMGDR DIVIGAGAWAGMDTPVQWATVIIPRVPFTGPNELFDTWTDEDAFRIGDPMTSYFDSKNAATRRLKQVFGRGLRHPDARCAIVICD PRISQLGDVAPVRFREGWFEGRRVEVVQSKAERNPALRRDALRHHGTDCQTCGHQPLILREVEVHHLNPIAEAKGMIATSMDD VAVLCRICHARAHKDGNNVIPLERLREIAKAKSRTEAQQLISF
DNA	1	TTTTTTTTTTT
DNA	1	TCTCGTTCACTCT
RNA	1	GAGUGAACGAGA
Ligand	1	ATP
Ion	2	Mg
Ion	1	Zn

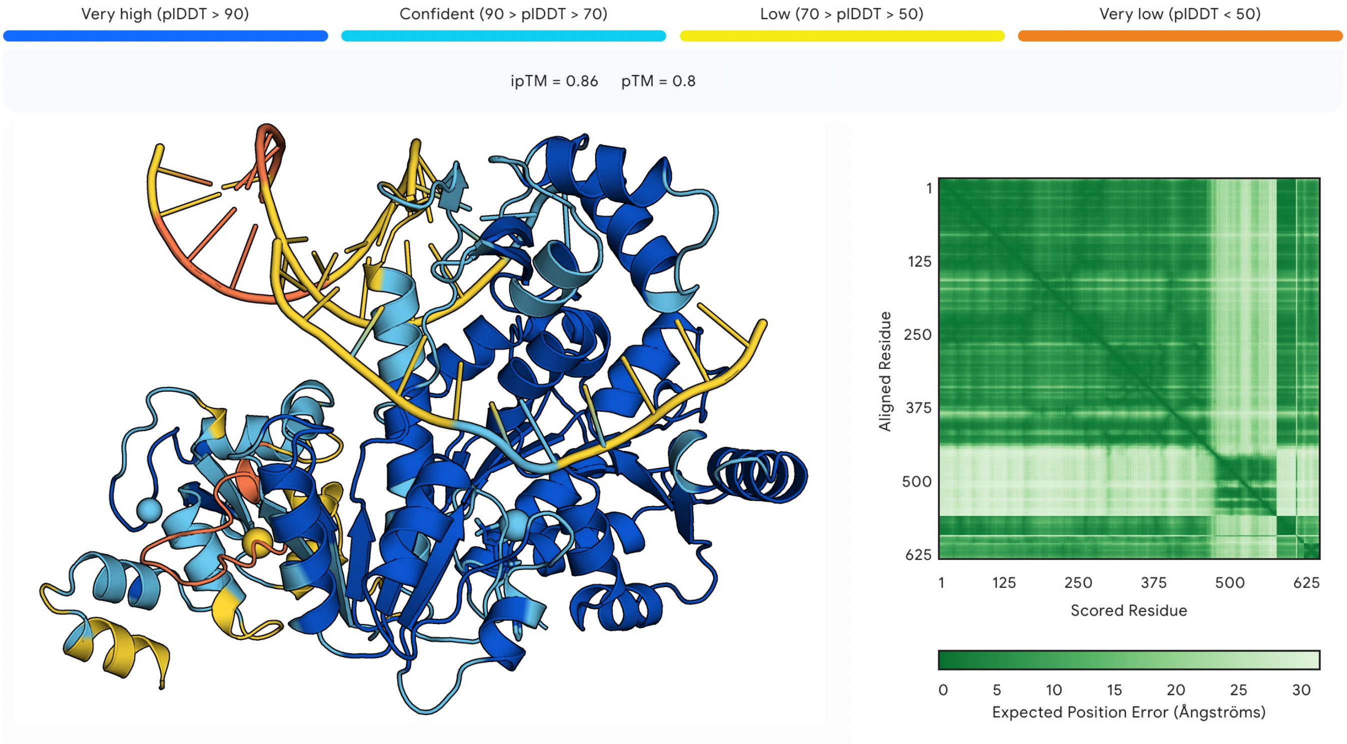

B

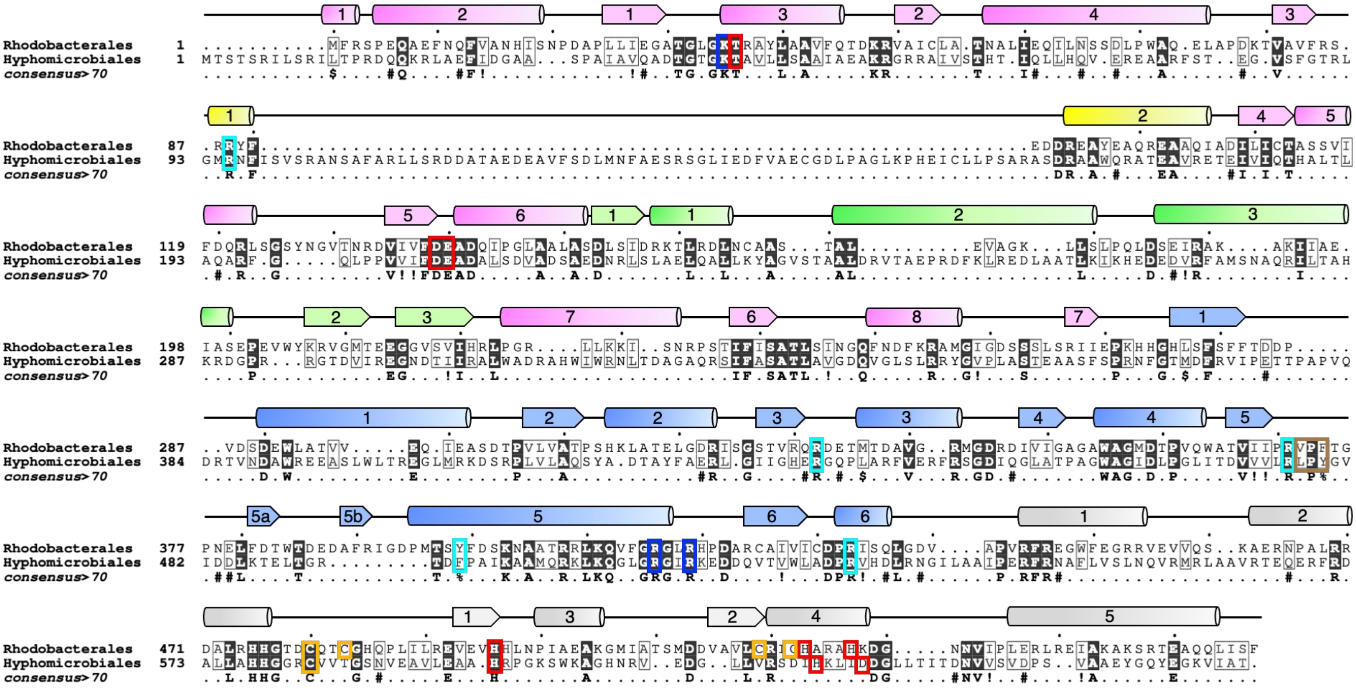
Rhodobacterales 1
Hyphomicrobiales 1
consensus>70
Rhodobacterales 87
Hyphomicrobiales 93
consensus>70
Rhodobacterales 119
Hyphomicrobiales 193
consensus>70
Rhodobacterales 198
Hyphomicrobiales 287
consensus>70
Rhodobacterales 287
Hyphomicrobiales 384
consensus>70
Rhodobacterales 377
Hyphomicrobiales 482
consensus>70
Rhodobacterales 471
Hyphomicrobiales 573
consensus>70

C

Input	Copies	Sequence
*No*CasDinG-HNH	1	MTSTSRILSRILTPRDQQKRLAEFIDGAASPAIAVQADTGTGKTAVLLSAAIAEAKRGRRAIVSTHTIQLLHQVEREAARFSTEGVS FGTRLGMRNFISVSRANSAFARLLSRDDATAEDEAVFSDLMNFAESRSGLIEDFVAECGDLPAGLKPHEICLLPSARASDRAAWQ RATEAVRETEIVIQTHALTLAQARFGQLPPVVIFDEADALSDVADSAEDNRLSLAELQALLKYAGVSTAALDRVTAEPRDFKLRE DLAATLKIKHEDEDVRFAMSNAQRILTAHKRDGPRRGTDVIREGNDTIIRALWADRAHWIWRNLTDAGAQRSIFASATLAVGDQ VGLSLRRYGVPLASTEAASFSPRNFGTMDFRVIPETTPAPVQDRTVNDAWREEASLWLTREGLMRKDSRPLVLAQSYADTAYFA ERLGIIGHERGQPLARFVERFRSGDIQGLATPAGWAGIDLPGLITDVVVLRLPYGVIDDLKTELTGRTDFPAIKAAMQRKLKQGLG RGIRKEDDQVTVWLADPRVHDLRNGILAAIPERFRNAFLVSLNQVRMRLAAVRTEQERFRDALLAHHGGRCVVTGSNVEAVLE AAHRPGKSWKAGHNRVEDGLLVRSDIHKLIDDGLLTITDNVVSVDPSVAAEYGQYEGKVIAT
DNA	1	TTTTTTTTTTT
Ligand	1	ATP
Ion	2	Mg
Ion	1	Zn

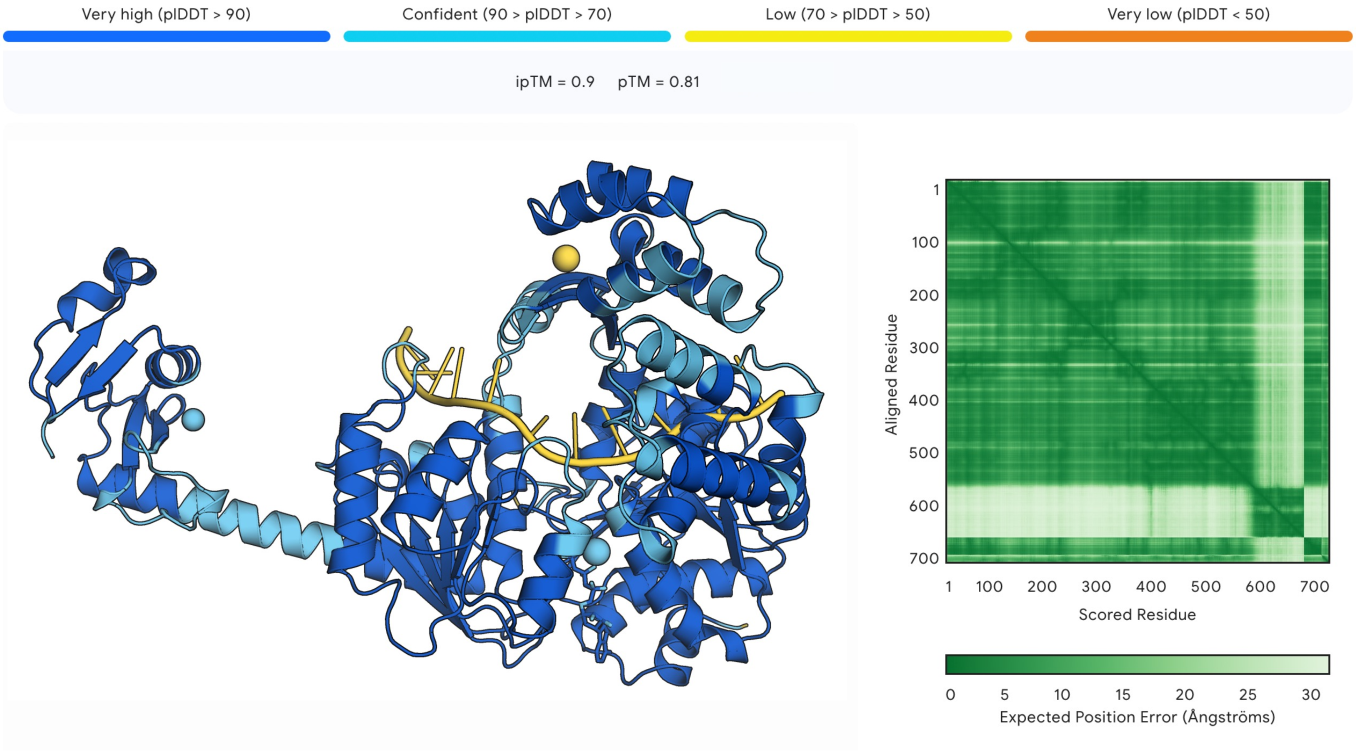

D

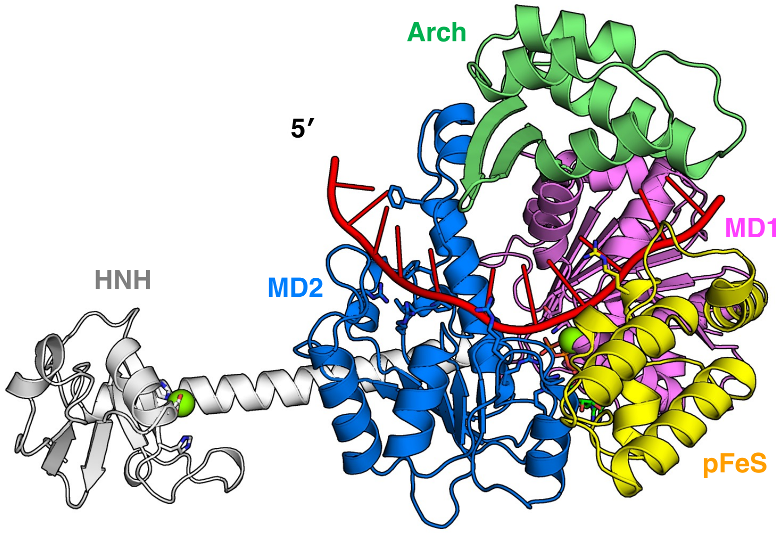

Arch
5′
MD1
HNH
MD2
pFeS

E

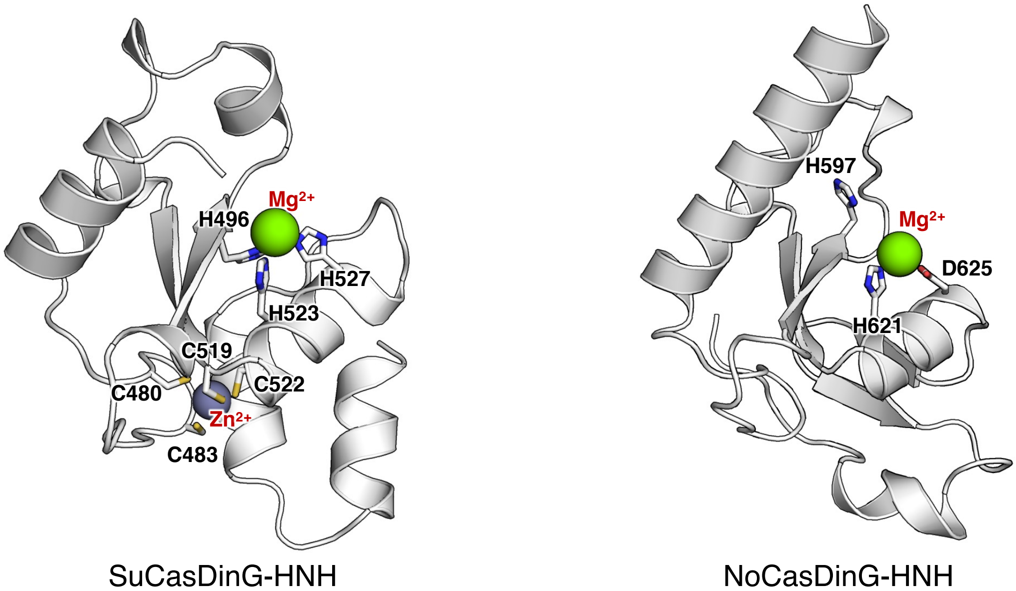

Mg2+
H496
H527
H523
C519
C480
C522
Zn2+
C483
SuCasDinG-HNH
H597
Mg2+
D625
H621
NoCasDinG-HNH
