# Supplementary material for: Structure, function and evolution of the bacterial DinG-like proteins
Source: Comput Struct Biotechnol J. 2025 Mar 17;27:1124–39. doi: 10.1016/j.csbj.2025.03.023 (PMC11981726; doi:10.1016/j.csbj.2025.03.023)
Supplement: Figure S5 — Supplementary material [file mmc5.pdf]

Figure S5 Supplemental information for ExoDinG subgroup proteins.

A. The structural model of the *Bacillus subtilis* ExoDinG–ssDNA complex in the presence of ATP·Mg^{2+} was predicted using AlphaFold 3. The exonuclease domain of BsExoDinG exhibits high sequence identity with that of E. coli RNase T. The reported structure of E. coli RNase T-DNA (PDB ID: 3V9X) demonstrates the ability to bind a 7-nucleotide ssDNA, with its active center likely utilizing a dual-metal ion catalytic mechanism. Therefore, an additional 7-nucleotide poly dT sequence and two Mg^{2+} ions were included in the input for the *Bs*ExoDinG-DNA complex structure prediction. The input parameters, including protein sequences, substrate information, and ligand specifications, are detailed in the corresponding figure. The model′s quality assessment are presented alongside the structural prediction.

B. Multiple sequence alignment of ExoDinGs was performed using Clustal Omega and visualized by ESPript. The names of corresponding bacteria species, protein IDs, and protein sequences were provided in Table S1. Secondary structural elements were depicted based on the AlphaFold 3 predicted *Bs*ExoDinG–ssDNA complex structure, displayed at the top of the sequences, numbered, and colored according to domain arrangement. Critical residues for metal coordination, ATP binding, DNA binding, and the P motif were highlighted in red, blue, cyan and brown boxes, respectively.

A

Input	Copies	Sequence
*Bs*ExoDinG	1	MNKQRFVVIDVETTGNSPKKGDKIIQIAAVVIENGQITERFSKYINPNKSIPAFIEQLTGISNQMVENEQPFEAVAEEVFQLLDGAYFVAHNIHFDLGFVKYELHKAGFQLPDCEVLDTVELSRIVFPGFEGYKLTELSEELQLRHDQPHRADSDAEVTGLIFLEILEKLRQLPYPTLKQLRRLSQHFISDLTHLLDMFINENRHTEIPGYTRFSSFSVREPEAIDVRINEDENFSFEIESWEAGNEKALSELMPGYEKRDGQMMMMREVADAFANREHALIEAPPGIGKTIGYLIPAALFAKKSKKPVIISTYSTLLQQQILTKDLPIVQDLFPFPVTAAILKGQSHYLCLYKFEQVLHEEDDNYDAVLTKAQLLVWLTETNTGDVAELNLPSGGKLLWDRLAYDDDSYKRSRSEHVIGFYERAKQIAMRSDLVITNHSLLLTDEGSHKKRLPESGTFIIDEAHHFERAASEHLGKRATYIELHTKLSRIGTLKEQGLLKKMRQLFQRNSLPVDSFFELEEWLQHVQAESDAFFSSVHSFVKRRKPKEDLNRLVFKVNKESQDKSWSILTDGAERLCSMLTHLQQLFEAQSSLMEKHLKGMKSKTVFLADEYQRSMKGLQHYCQTLQKLFFGSDDDEAVWIEIDAKGAKNAVAIYAQPLEPGELLADQFFARKNSVVLTSATLTVEGSFQFMIERLGLSDFFPRTMRIESPFSYDERMQVMIPKEMKSIQDTGQPEFIQDTARYIELMAKEKQPKILVLFTSHDMLKKVHQELKHNMSASGIQLLAQGITGGSPGKLMKTFKTSNQAILLGTNHFWEGVDFPGDELTTVMIVRLPFRSPDHPLHAAKCELARKKGKNPFQTVSLPEAVLTFRQGIGRLLRSAGDKGTIIILDRRIKTAGYGRLFLDALPTTSVSEMTDSELEAYVAGEKE
DNA	1	TTTTTTTTTTT
DNA	1	TTTTTTT
Ligand	1	ATP
Ion	3	Mg

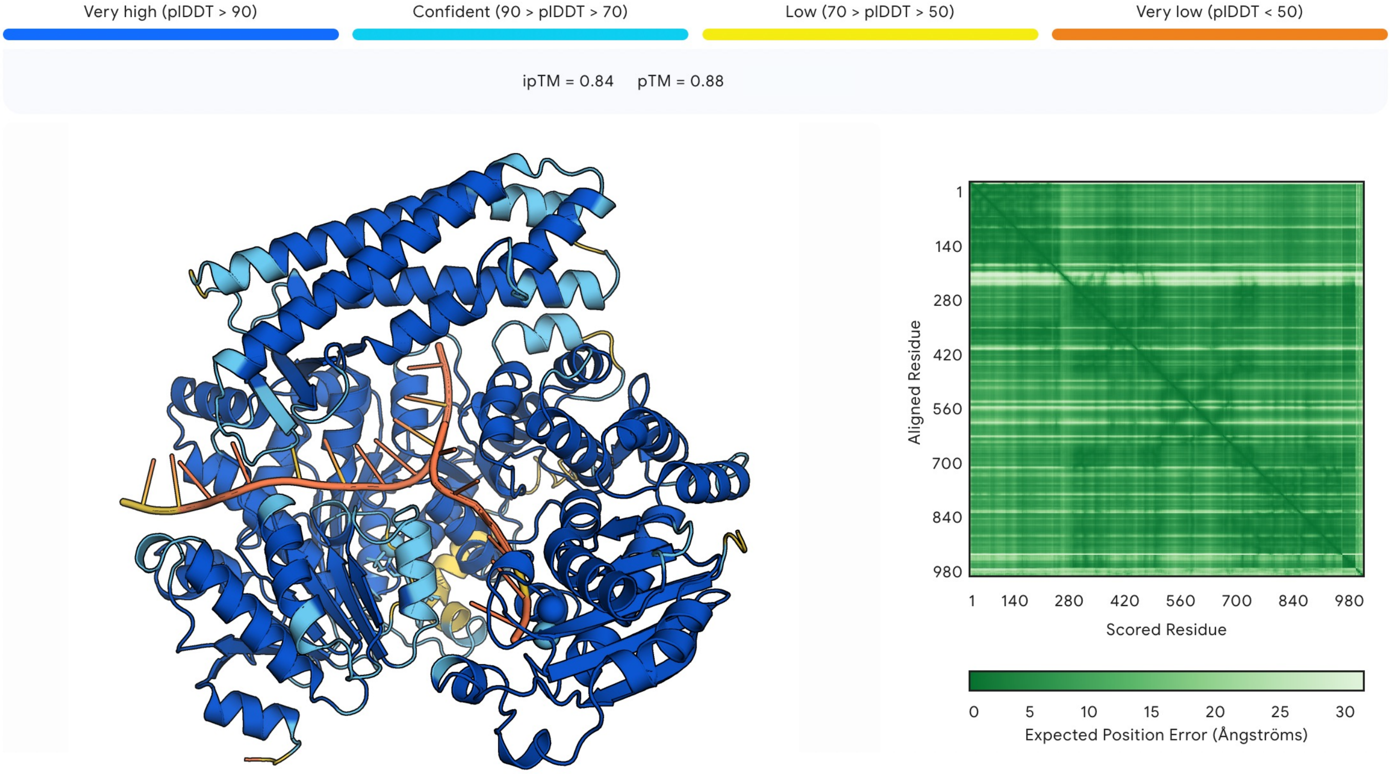

B

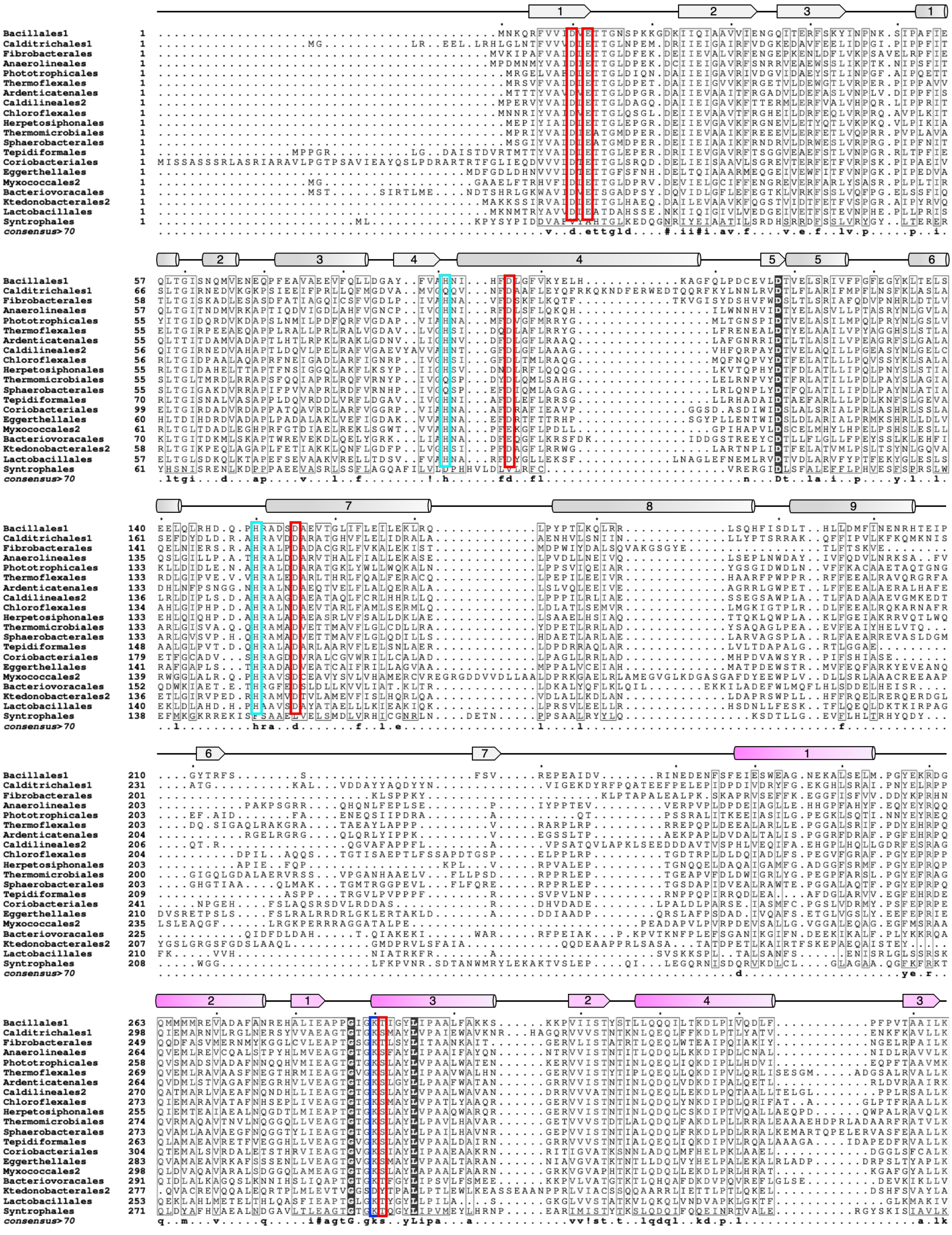
Bacillales1
Calditrichales1
Fibrobacterales
Anaerolineales
Phototrophicales
Thermoflexales
Ardenticatenales
Caldilineales2
Chloroflexales
Herpetosiphonales
Thermomicrobiales
Sphaerobacterales
Tepidiformales
Coriobacteriales
Eggerthellales
Myxococcales2
Bacteriovoracales
Ktedonobacterales2
Lactobacillales
Syntrophales
consensus>70

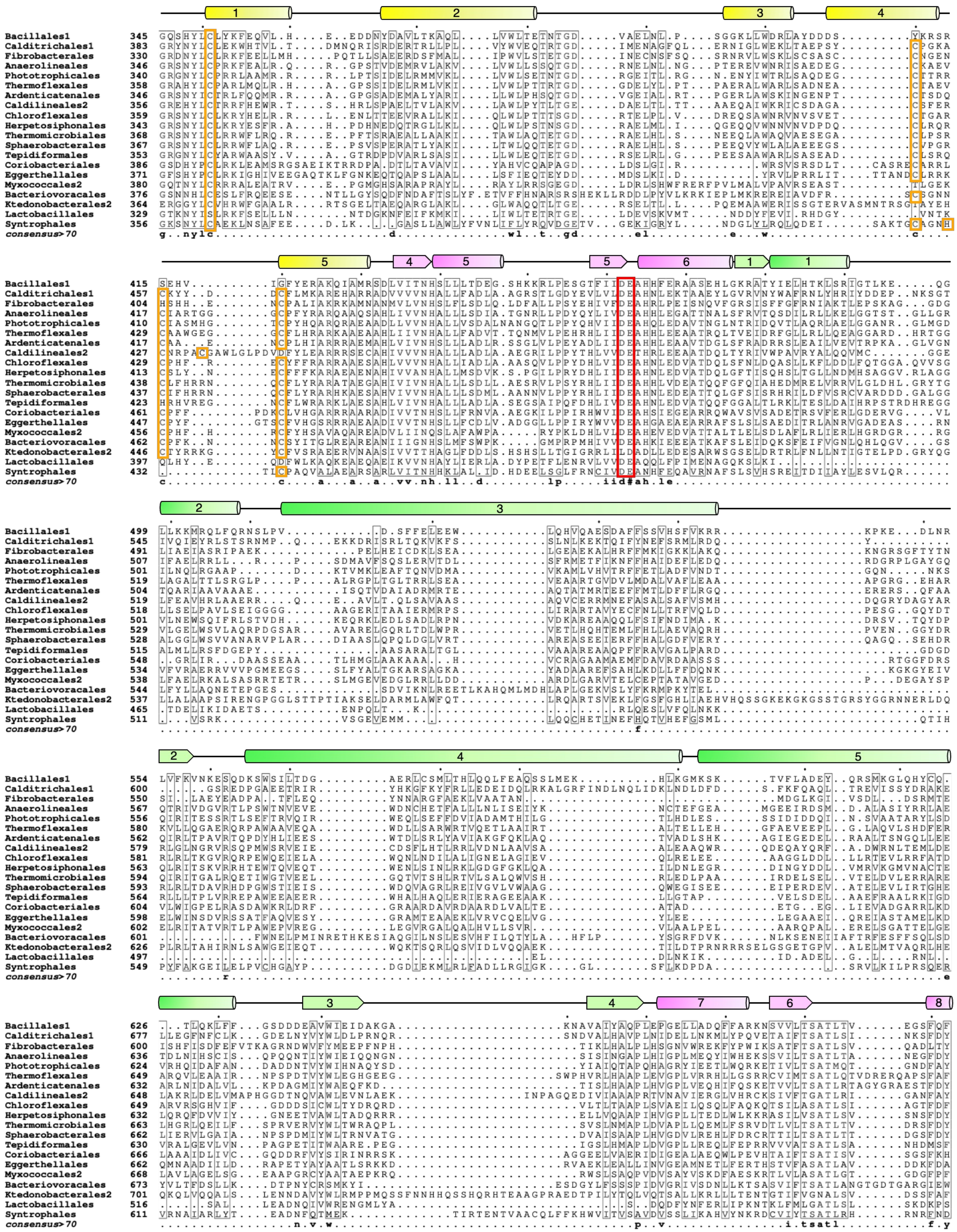

Bacillales1 345
Calditrichales1 383
Fibrobacterales 330
Anaerolineales 346
Phototrophicales 340
Thermoflexales 358
Ardenticatenales 346
Caldilineales2 356
Chloroflexales 359
Herpetosiphonales 343
Thermomicrobiales 368
Sphaerobacterales 367
Tepidiformales 353
Coriobacteriales 386
Eggerthellales 371
Myxococcales2 380
Bacteriovoracales 376
Ktedonobacterales2 364
Lactobacillales 329
Syntrophales 356
consensus>70

```
Bacillales1           693  MIERLGLSDFFPR.......................T.MRIESPFSYDERMQVMIPKEMKSIQDTGQ.PEFIQDTARYIELMAKEKQPKILVLFTSHDMLK
Calditrichales1       747  FHSRLGLKDIKDRP...................VIS.AAFDSPFDYETQLLLAVTDFLPDP...RS.ADFPAAVKELVIRLHKEHRRGLLILLTNYSMLN
Fibrobacterales       671  FLQKMGMDNLRLGK..................QPFVRVYTEQSDVNERRSVMVAKFLPKP...SA.PEFGDALNDTLLKVLPNVEENTMVLFTSVATMM
Anaerolineales        704  IRNRLYADEANE.......................LALGSPFDYESSALLYIVNDIPEP...SDANGYQRELERVLVRLAKATQGRMLVLFTSYAQLK
Phototrophicales      692  MKDRLHAERLRA.......................STVGSPFDYEKSAMIYIPEDMPEP...NR.PNYQKMVERGIINLATALNGRVMVLFTSYSQLK
Thermoflexales        726  LKERLGAWEMEE.......................LALDSPFDYRRNALVYLVTDIPEP...GQ.AGYQRAVEQAVLEAARAIGGRTMVLFTAYAPLR
Ardenticatenales      704  LRDRLHAHDADE.......................LAVGSPFDYKNSTLLYLVTDIPEP...NQ.PGYQRMLEAAIVDTATALGGRTLVLFTANNHLS
Caldilineales2        726  LRDRLGLWDVKI.......................ATVDSPFDYRRNVLLYLPTDMPPP...DD.ARYQTMVEQAVVTAASACRGRTLVLFTSHQQVR
Chloroflexales        716  VKSRIGLEDCQE.......................LMLDSPFDYAQQALVYIPNDIPEP...NQ.RGYQPMIEQAIIDLAIASGGRMLALFTASNALR
Herpetosiphonales     699  PKQRLGLDEATT.......................MQLDSPFDYSKSTLIYLPTDMPEP...NE.RNYQRAMEDALINLCKATGGRTLALFTANASLK
Thermomicrobiales     731  IRQQLGLADADE.......................LVVESPFDYASSTLLCLAEDMAEP...GE.PGYQRQVNEALLALLRVTRGRALVLFTSHSALQ
Sphaerobacterales     730  VRQRLGIDEAHE.......................LQVPSPFDYPATTLLYLADDVPEP...GQ.PGHQRHVQEALIDLCKATRGRAMVLFTSHSALQ
Tepidiformales        697  AAGRLGLDSPRM.......................LELGSPFDYERAALLAAVDDLPEP...NH.PDFPGRLARAIGTLALASRGRAIALFTSNALLR
Coriobacteriales      736  FDHQVGLDRLGADA..................H.RDLHLESSYDFDENMAVVVAADMPDP...RDRDAYIDALENILCDVHMAMGGSTLTLFTNRRDME
Eggerthellales        732  FESALGLNASEHST..................C.QMCKLDSSYDFDAHMTVYVASDMPEP...NE.AAYLAALQRLLVDVHRAQNGSMLTLFTNRREME
Myxococcales2         736  VLRRLGLDGRGGRP..................APRLVRAATPFKLHEQALVVLVTDAPRA...HE.EAFVEWASLRISGLAQTMGGRVLGLFASTRRME
Bacteriovoracales     745  ATGYSYLEPERR....................FKAGTYLPFTFDYKKNTKVFLCDDGPSL...YQ.ANFVEHHLKHIKPLIQKLGGRTLLLFSAKSRFE
Ktedonobacterales2    792  TQGRLGLEQDNQ.......................TALSLVTAHNEQTLLCLPNDVPEP...NA.PHYQRHLDETLIQMATMLEGQLVALFTSHAALR
Lactobacillales       569  FPDLLGFTDYRF.......................FKIEGNQA..TNQQLLTVTDAPNIKNLSL.IDYADYTANSIGEL.SKLNLPMVVLFTSKMALT
Syntrophales          688  FCRIVGMDDATGCDSISPAGKEDRTFFTDQDGREFRFVAIPSPYSLE.SMKVFLPDGAVNGGYENK.QAWLKAVVDLLPELIRKNRGRTLVLFSSYSDLE
consensus>70               ....lgl.d...........................spfdy.........d.pep.......y..........l.....g..l.Lft....l.

Bacillales1           769  KVHQELKHNMSASGIQLLAQGITGGSPGKLMKTFKT.SNQAILLGTNHFWEGVDFPGDELTTVMIVRLPFRSPDHPLHAAKCELARKKGKN..PFQTVSL
Calditrichales1       823  QMYDWLKPHFDGEHVLLLAQGK.SGSRTNILNQFKE.NRASILLGTDSFWEGIDVPGDALELLFIPKLPFDVPSEPVIAARMEAIKRRGGN..PFMEYSL
Fibrobacterales       748  KAQAALAPAFAERNKLLLCQHV.DGSLDGLVAMFRK.ERGACLLGCQSLWEGVDFPGDALKLLVITKLPFPNPSDPLVAGLTNEMKAANKN..FFKDYFI
Anaerolineales        776  KTSQVITPLLADQGILVYEQGE.GASTHTLLATFRE.ADKAVLLGTRAFWEGVDIPGEALSVLVITKLPFDVPSDPIIAARAETF....ED..PFNEYHL
Phototrophicales      763  ETATNIAARLALGDIVVYDQAT.GTSRESLLDNFKS.TEKAVLLGTRSFWDGVDIPGEDLSALVIVRLPFAVPTDPVFAARSEMY....DD..AFHNYAV
Thermoflexales        797  RAARALADPLARLGISVYEQGD.GSSRRQLVERFRS.AERAVLLGTRSLWEGVDIPGEALSCLIIAKLPFEVPDEPIFAARSARY....RD..PFLDYAV
Ardenticatenales      775  QTATAIESTLTARNITTLAQTQ.GMSRQQLLEQFRREGSRTVLLGTRSFWEGVDVPGPALTALIIAKLPFDVPSDPIFAARSETY....ES..PFFEYSI
Caldilineales2        797  ATAEAIRPTLERMGITVLQHGQ.GG.RSRLLREFRA.AERAVLLGTRSFWEGIDLPGDEVRCLLIARLPFAVPNDPLVAARSADF....ED..SFNEYMV
Chloroflexales        787  QTYTAIQEVLEDHGIGVMAQGI.DGSRKALVDRLKE.FPRSVLLGTNSFWEGVDVVGDALSVLVITKLPFAVPTDPIVAARSELF....TD..PFNEYSV
Herpetosiphonales     770  QTYHGISESLEQADISTLAQGM.DGSRRSLIQRFKS.DPRTVLLGTASFWEGVDVVGDALSVLVITKLPFSVPNDPVFSARSEGF....DD..AFAEYSV
Thermomicrobiales     802  AAYRAIKRPLEQEGILVLGQRL.DGSPRQLVERLKA.RPATVVLGTNAVWEGVDVPGEALSVLVIAKLPFAVPSDPVFAARCESY....SD..PFQEYAV
Sphaerobacterales     801  TTYRAIKRPLEAEGILVLAQRM.DGSPRQLIDRLKS.HPETVLLGTNSFWEGVDIVGDALSLLVITRLPFSVPSDPVFAARSELF....ED..PFNSYAI
Tepidiformales        768  HVSGLVRPMLEPSGIVVLAQGI.DGTPRWITEQLRE.NPETLVLGSASFWEGVDIRGEALSLVMITRLPFDVPTDPVGRARAEQY....DD..PFNQYQV
Coriobacteriales      813  TLFERVEPRLARAGLELGCQLR.GAPGKQLRDRFVQ.KTESSLFALKAFWEGFDASGETLRCVVIPKLPFTSPTDPLSCERSLRE....DR..AWAKYAL
Eggerthellales        808  RCFDEVQPQLKVDDLRVVCQKW.GVSVKGLRDDFLA.DEHLSLFALKSFWEGFDAPGATLKGVVIPKLPFAKPTDPLSCERAARD....DQ..AWRRYVL
Myxococcales2         813  RVGTSVRGRLDPLGIEVLRQSR.GHSR.SLAARQEK.DTGTVLLGTKSFWQGVDIPGRGVGCVFIDKLPLEPAMRPLVAAREEPLSRSGGEYMGFLHYRL
Bacteriovoracales     820  VAREILLKEFE.GQIPLFIQGM....GTSVVEDFKR.SGSGILLGMESFGEGIDIPGDALQFIFIDKVPD.LRMDLVINERRDFYEANLGN..EFTDYYL
Ktedonobacterales2    863  SSYATIKPLLETRGILVLGQGI.DGSPRQLWQAFQR.QERVVLLGTGSFWEGAEDINRTPACIVVTRLPMPVLNDPPIAARAEQY....SD..QLHNFTV
Lactobacillales       640  ....FVAEKLSAEGFEILAQDI.NGSPAQIKKKFDK.GEGKILLGLGSFWEGVDFDKQNQVILVIPRLPFATPDDILTKKYAQKF....EN..PFYDFNV
Syntrophales          786  AVALQTGDEIIADGYPLLIQQM.GRPTGNLIDEFRS.IRESVLFGVETFWYGVDFRGDTLTQVIITRIPFPYPFDPLQTARKGSL....SKE.EFWSRYY
consensus>70               .....i...l....i..l.q.....s...l.d.f.......vllg..sfweGv#..gd.l...v!..lPf..p.dp...ar.e.......d...f.ey..

Bacillales1           866  PEAVLTFRQGIGRLLRSAGDKGTIIILDRRIKTAG..YGRLFLDALPTTSVSE.MTDSELEAYVAGEK....E...................
Calditrichales1       919  PEAIIKFRQGFGRLIRHKKDFGAVIIGDNRVSRMR..YGKHFLNSISGRKEIV.FDEESFFELLHEWFKNRPQENGNDAQ............
Fibrobacterales       844  PEAYIELRQGMGRLLRSDSDSGKVLILDNRVVLER..YGKTFARIWNFKNRIA.GSVSDIERFVK...........................
Anaerolineales        868  PEAILRFRQGFGRLIRTQSDRGIVVVLDKRILTKR..YGRAFIESLPTCTLKV.GGSEDLPGIARRWLNL......................
Phototrophicales      855  PNAILRFRQGFGRLIRTQHDRGIVTIFDSRIINKK..YGSQFLEALPACTVQR.GPLDNLGAAAQHWLSTTKA...................
Thermoflexales        889  PEAVLRFRQGFGRLIRRRSDRGVVVILDRRVLTRT..YGAWFLTALPPCTLHR.GPLSDIGSVLKDWLMGAAAK..................
Ardenticatenales      868  PEAVLRFRQGFGRLIRRGSDEGVVLVLDKRLLTKR..YGGMFLEALPGCTVLR.QRADRIEELIGRWMTRDRSRKL................
Caldilineales2        888  PDAVLRFRQGFGRLIRRASDRGVVVLLDSRIWRKA..YGQTFLDSLPECTLRR.APLSNLAAAIETWFNLAYE...................
Chloroflexales        879  PQSILRFKQGFGRLIRSREDRGIVVVLDRRLLTKK..YGQQFLDSLPPTRVRT.GTLASLPGLVARFLQKVNGSSG................
Herpetosiphonales     862  PQAILRFKQGFGRLIRSKDDRGIVVVLDRRLLSKN..YGRQFLESLPDCTIQR.KPLAELATTAARWLV.......................
Thermomicrobiales     894  PQAVLRFKQGFGRLIRSTRDYGVCVVLDRRVTTRR..YGQLFVQSLPPCTVKR.VPVAQIPRLAQDWLVRQQDRLAARVPAEGGSGDGSAGS
Sphaerobacterales     893  PQAVLRFKQGFGRLIRSSTDRGVCVVLDRRTVSRR..YGSSFVRSLPDCTVVV.GPTSELPARASEWLQARPASASV...............
Tepidiformales        860  PAAILRFRQGFGRLIRDKRDRGVVAVFDRRLWEKR..YGRQFIDSLPPCTRFR.GSTDEVAAAIEDWLSRG.....................
Coriobacteriales      905  PDAVLEIKQAAGRLIRSSSDRGVLVLADSRLMTKG..YGKKFLHSLPNSSYQR.IETAQMGRFLKLWRRSHEHRRG................
Eggerthellales        900  PAAVLETKQAAGRLIRKADDTGVLILADRRLLTKS..YGKAFLNSLPSRTIKV.MTAAEIVADLE...RSNN...G................
Myxococcales2         910  PRALLQLRQGVGRLIRATTDRGVVIISDPGH..PS..YRGHLMNALEGYRVEA.LPWAQARLRIHAMLKETGLTVE......SGPAR.SWG.
Bacteriovoracales     911  AHRTRSLHQKLGRLLRTEQDFGGVIIVDNRVKTWKGKTMEKLVKLMEPYSLER.ATMAEATKEIEEFILGKGSETQL...............
Ktedonobacterales2    955  PVASLRLRRGLNRLAWKDARRKAVVLFDRRITSKE..YGSTILHSLPPCSQRQ.AAISHMPEMILDWLTGTGSWE.................
Lactobacillales       728  PMATLKLRQAIGRVNRRKNQYSSIVVLDKRLAGKS..YAKRMRKNLSETLPVKMLNLLDTISEIKNFLI.......................
Syntrophales          879  YEAIIKLKQGMGRLIRSETDKGRLVFLDSRCRRFEDLWS.............................................
consensus>70               peavl...qg.gRlir...d.g.vvv.D.r.......yg..f...lp..........e.................................
```
