# Supplementary material for: Structure, function and evolution of the bacterial DinG-like proteins
Source: Comput Struct Biotechnol J. 2025 Mar 17;27:1124–39. doi: 10.1016/j.csbj.2025.03.023 (PMC11981726; doi:10.1016/j.csbj.2025.03.023)
Supplement: Figure S6 — Supplementary material [file mmc6.pdf]

Figure S6 Supplemental information for pExoDinG subgroup proteins.

A. The structural model of the *Caulobacter vibrioides* CB15 pExoDinG–ssDNA complex in the presence of ATP·Mg^{2+} was predicted using AlphaFold 3. The putative exonuclease domain of CvpExoDinG exhibits high similarity of the topology with the exonuclease domain of BsExoDinG. Therefore, similar as *Bs*ExoDinG, an additional 7-nucleotide poly dT sequence and two Mg^{2+} ions were included in the input for the *Cv*pExoDinG–ssDNA complex structure prediction. The input parameters, including protein sequences, substrate information, and ligand specifications, are detailed in the corresponding figure. The model′s quality assessment metrics are presented alongside the structural prediction. Despite setting extra DNA sequence and metal ions as input, the pExo domain failed to incorporate high-quality models for DNA and catalytic metals.

B. Multiple sequence alignment of pExoDinGs was performed using Clustal Omega and visualized by ESPript. The names of corresponding bacteria species, protein IDs, and protein sequences were provided in Table S1. Secondary structural elements were depicted based on the AlphaFold 3 predicted *Cv*pExoDinG–ssDNA complex structure, displayed at the top of the sequences, numbered, and colored according to domain arrangement. Critical residues for metal coordination, ATP binding, DNA binding, and the P motif were highlighted in red, blue, cyan and brown boxes, respectively.

A

Input	Copies	Sequence
CvpExo DinG	1	MTASPPTLDLAPALVVLPGPRAGLADGGAARMLRAPDARDLFEHGPVLVAHAAMTARRLNLSPPPRSPRLFDVLELHAFVRPAA FCAPSAVGLATALGLREPHGAAEQAQTLREAADALLRELALTPVPSREEALAIAETLAKAGWSWGPAVIGALRSVPVGNQFRGS GLDVWARLVEWEDQAPPGEAGSRPIDPERAGERLTELLQRSGLEEVREAQATFAREATFAFQPREREGEPRMMLAEAGTGVGKT LGYLAPASLWAEANGPSVWVSTYTRALQRQIERESRSIYPDPKERARKAVVRKGRENYLCLLNFQEQINGAQLGNGDLIGLALT VRWARATRDGDMTGGDFPAWLPTLAAVPPSVQASPANLVDRRGECIHAGCQHYRICFIEKAVRASKRADLVIANHALVLTQAAF DGARTARGLKGDNETTSLKRIVFDEGHHLFEAADSAFSAALSGAEAAELRRWIRGPEGRGRRGRGLEARLLDILGDREGARGA MSQAIQAAAALPGEGWSGRVAPPDGQINPIGPIENFLVAVIEQLRARSGDRGGADLGLQCDARPATDLVRERAPEAAKALAAIEA PLLALARALEDVLDEDAEHLGASERARIEGALRGLDRRARMTLPAWRSILKAIEDDDVEPSDSDPDFVDWFEATFLYGRVVDAA CRRHWVDPTEPLRAAVLSPAHGVLVTSATLVDPALEDPFALAEMRTGAARLPTAPKVLRLVSPFDYENNAKAFVVTDVNKEDPR QVSAAMRELFLAAGGGGLGLFTAIRRLKAVHERIAAPLADQGLALYAQHVDPLEVGALVDIFRAEEDACLLGTDAIRDGVDVPG RSLRLLVFDRVPWPRPDVLHKARRLRFGGKGYDDAVARARISQAFGRLIRRADDRGVFVMLDAAAPTRLFSSLPEGVTLERVSL VEAIEATGAFLAKKEA
DNA	1	TTTTTTTTTTT
DNA	1	TTTTTTT
Ligand	1	ATP
Ion	3	Mg

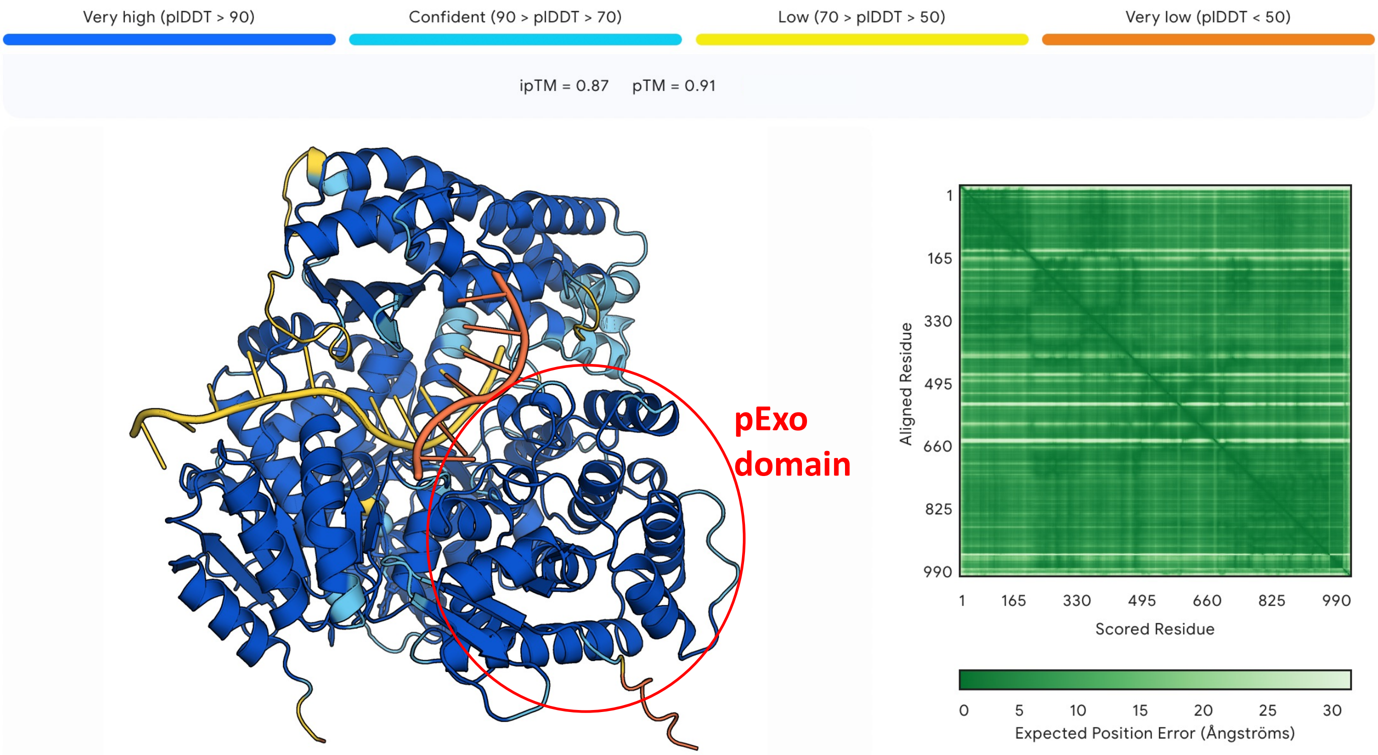

B

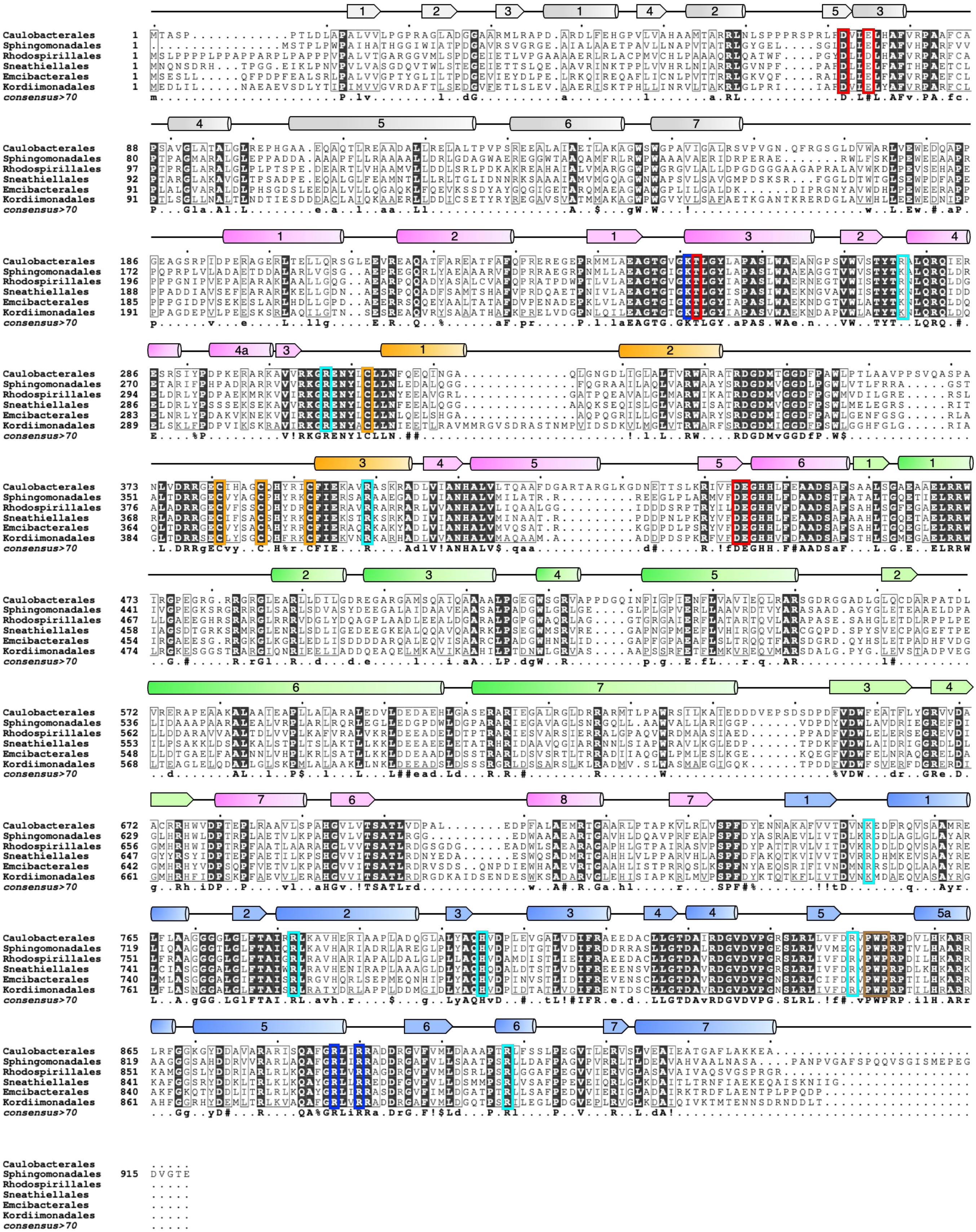
Caulobacterales
Sphingomonadales
Rhodospirillales
Sneathiellales
Emcibacterales
Kordiimonadales
consensus>70
