# Supplementary material for: Structure, function and evolution of the bacterial DinG-like proteins
Source: Comput Struct Biotechnol J. 2025 Mar 17;27:1124–39. doi: 10.1016/j.csbj.2025.03.023 (PMC11981726; doi:10.1016/j.csbj.2025.03.023)
Supplement: Figure S7 — Supplementary material [file mmc7.pdf]

Figure S7 Supplemental information for EndoDinG subgroup proteins.

A. The structural model of the *Bdellovibrio bacteriovorus* HD100 EndoDinG–ssDNA complex in the presence of ATP·Mg^{2+} was predicted using AlphaFold 3. The endonuclease domain of BbEndoDinG exhibits high sequence identity with that of human exonuclease V. The reported structure of human exonuclease V-DNA (PDB ID: 7LW8) demonstrates the ability to bind a 7-nucleotide ssDNA, with its active center likely utilizing a dual-metal ion catalytic mechanism. Therefore, an additional 7-nucleotide poly dT sequence and two Mg^{2+} ions were included in the input for the *Bb*EndoDinG–ssDNA complex structure prediction. The input parameters, including protein sequences, substrate information, and ligand specifications, are detailed in the corresponding figure. The model′s quality assessment metrics are presented alongside the structural prediction.

B. Multiple sequence alignment of EndoDinGs was performed using Clustal Omega and visualized by ESPript. The names of corresponding bacteria species, protein IDs, and protein sequences were provided in Table S1. Secondary structural elements were depicted based on the AlphaFold 3 predicted *Bb*EndoDinG–ssDNA complex structure, displayed at the top of the sequences, numbered, and colored according to domain arrangement. Critical residues for metal coordination, ATP binding, DNA binding, and the P motif were highlighted in red, blue, cyan and brown boxes, respectively.

A

Input	Copies	Sequence
*Bb*Endo DinG	1	MRKVSLDVRQFALPCPRRGSIELHSGYGAPPMSGQEIHMAIQRRRQREFDDYTPEKKMSWVFEAGPYEFHISGRADGITENPVQ<br>IEEIKTAFDVEELWRKLRSDDNHPYIWQLRTYGYFHYKETGRIPFLNLHLVSSRNFKSMDLRVELDIAHYELWLALRLDELVEET<br>KVKEKLFKARQKMAEEMSFPFATPRPGQRELIEGIEAQVADEHPLLVQAPTGLGKTVGVLYPNLKDSLSRGQKTVYVTPKNSQH<br>IVAEEAVEKLQEQGSKIRSLTLTAKSKMCLKAETLCNPGYCEFARDYYTKLAEHDLVNKLSKKRKLTQEKLVEMGKEFEVCPFEL<br>SVEAIERADVVIGDYNYAFAPRSLLGRLSEPLLEAGEKPNLVIDEAHNLPSRAQDYFSPSLSVQELDILEGDFTKLPPTFSLQAGSL<br>IRKAKSLIQEYGEDGGSRKVDIDIEPFLEHERSIRALTTEYLDSDTEIITRDPMLRLMNLWSEFIAALEYRGPEFFTTYQNSRFTEM<br>LKVTCCDASEQLKIAYKQFKNVVAFSATLKPFTYYQELLGFDLEKSKTLEFQSPFKPENRQLMIIPQISTKLSDRVVSSGKVAEVIS<br>RVTRVKAGNYIALFPSFEFLAQVEKQLQVPHLRVLRQERDMKQLDVQIYLEELKAANEPILLLGVQGGVFSEGVDFPGDMLIGA<br>FVIGPALPSFDFEREQIRTYFDGRYGKENGFNYTYVYPAMAKAIQSAGRVIRSETDKGVIILMDSRFLNPVYAEAMPQGWFKESP<br>RELVSQKILADLETFWKNSDTPCS
DNA	1	TTTTTTTTTTT
DNA	1	TTTTTT
Ligand	1	ATP
Ion	3	Mg

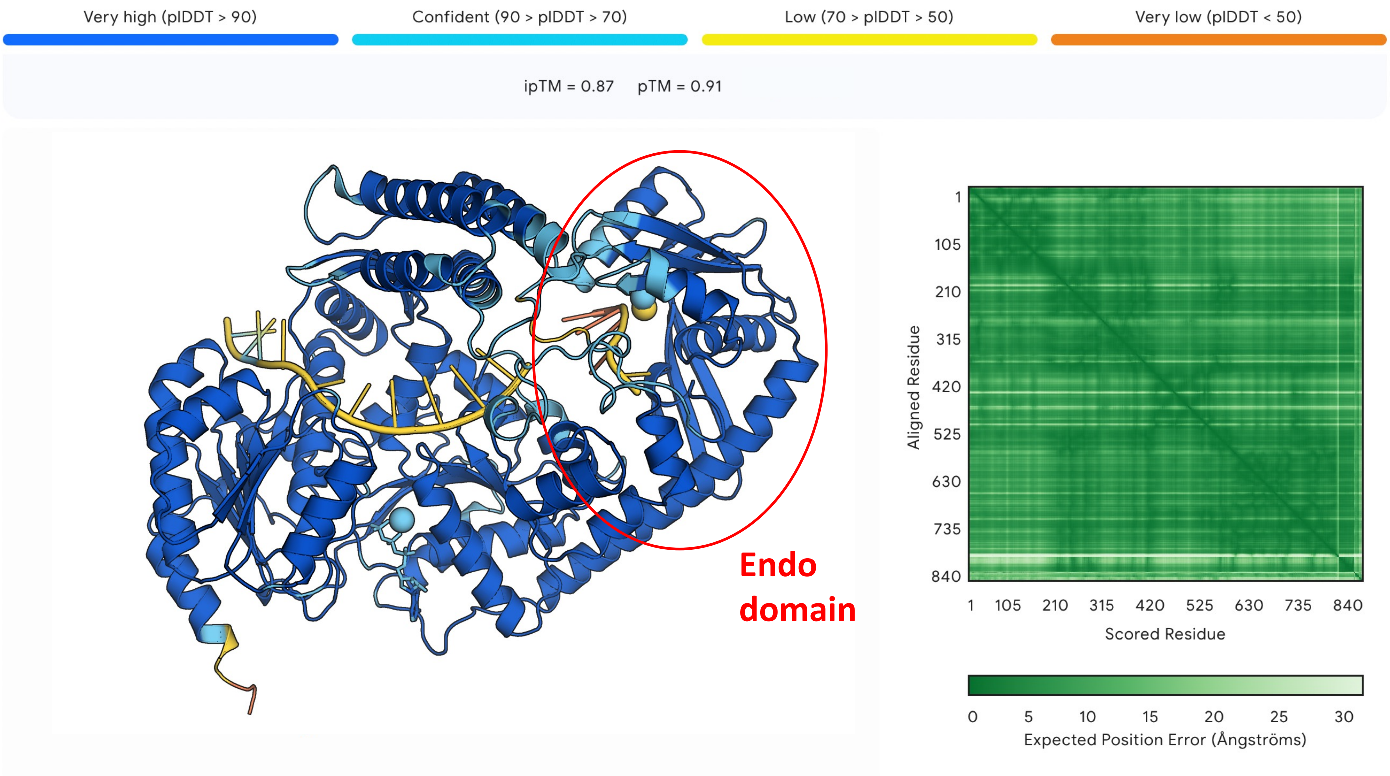

B

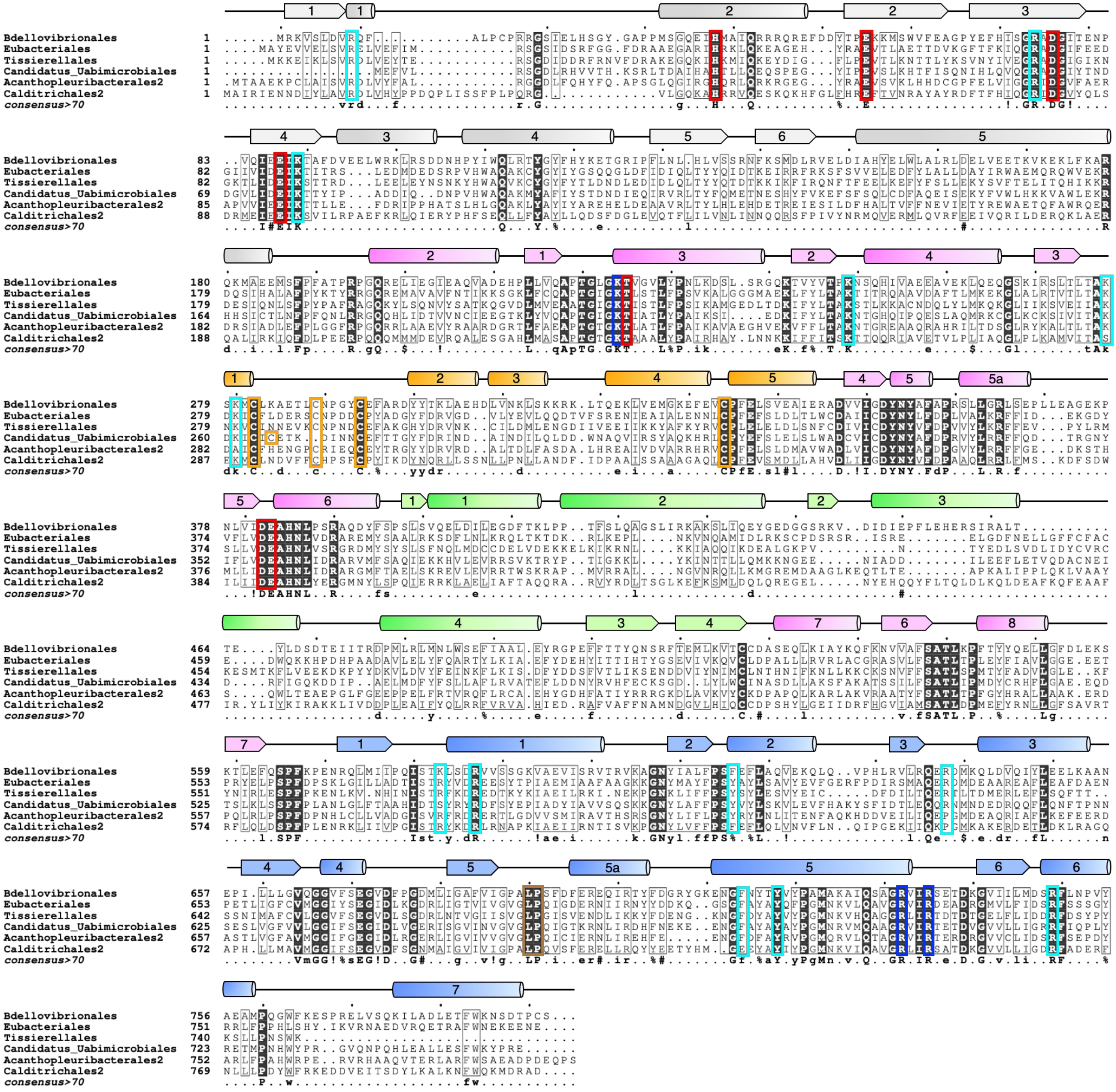
Bdellovibrionales
Eubacteriales
Tissierellales
Candidatus_Uabimicrobiales
Acanthopleuribacterales2
Calditrichales2
consensus>70
