# Supplementary material for: Structure, function and evolution of the bacterial DinG-like proteins
Source: Comput Struct Biotechnol J. 2025 Mar 17;27:1124–39. doi: 10.1016/j.csbj.2025.03.023 (PMC11981726; doi:10.1016/j.csbj.2025.03.023)
Supplement: Figure S8 — Supplementary material [file mmc8.pdf]

Figure S8 Supplemental information for RadC-like DinG subgroup proteins.

A. The structural model of the *Desulfuromonas soudanensis* RadC-like DinG–ssDNA complex in the presence of ATP·Mg^{2+} was predicted using AlphaFold 3. The RadC-like domain of DsRadC-like DinG exhibits high similarity of the topology with PfJAMM1. The reported structure of PfJAMM1 (PDB ID: 5LDA) coordinates a Zn^{2+} in its active site. Therefore, a Zn^{2+} ion was included in the input for the DsRadD-like DinG–ssDNA complex structure prediction. The input parameters, including protein sequences, substrate information, and ligand specifications, are detailed in the corresponding figure. The model′s quality assessment are presented alongside the structural prediction.

B. Multiple sequence alignment of RadC-like DinGs was performed using Clustal Omega and visualized by ESPript. The names of corresponding bacteria species, protein IDs, and protein sequences were provided in Table S1. Secondary structural elements were depicted based on the AlphaFold 3 predicted DeRadC-like DinG–ssDNA complex structure, displayed at the top of the sequences, numbered, and colored according to domain arrangement. Critical residues for metal coordination, ATP binding, DNA binding, and the P motif were highlighted in red, blue, cyan and brown boxes, respectively.

A

Input	Copies	Sequence
*De*RadC-like DinG	1	MEISYHQPALLAMRQAIAQAQGNEVFFLGRTDEERRVVEVEVLARGKEDAVPAILQLCRYGDVVIHNHPSGGLQPSSADIEIASR LGSLGVGFHIVDNSVGNVYKVVEAFAPREEQHLEPQRIGDILGPQGVVATTLPGYEDRPEQLRMAFAVGEAFNAGKLAVIEAGT GTGKSLAYLAPAILWALTNEERVVVSTNTINLQEQLVRKDLPFLQRATGLEFRAVLVKGRGNYLCLRRAENARLETGLFDEEHAA ELHSIVEWAERTADGSREELPFLPPEQVWEEVRCELDQCGRVRCPHYARCFFHKARRKAAQADILVVNHALLLSDLALRAQTDN YSAAAVLPPFDRIILDEAHHLEDVATNYFSSQVTRFAFARVLNRLRHPRQPDKGLLPRYLSALAKELPDSADELYRDLHGRIEKLL AGRQALFDLAVRDLESIGTDLSEGLSRPVREGEELKHRVLPAFTETPLWEGIAARVRALGLATNELAKGVRALLRAGDKIPEEYA EKLGNFFTDLRGIAGRLEGIAADLDFFVAKEEQTCAWFEVALGRVGRGTSLVTKLCTAPLEVADNLKAAVYDRFRTVVLTSATLA VGPSFDYFKCRVGLDRVEPGRVTELLLHSPFDFARQTLVAIPTDVPEPGRPGYQEMVRDLSEQAILSADGRTFVLFTAYSLLRRVH GELAPILSARGYHCLRQGDDNRHRLLKKFAADPTSVLFGTDSFWEGVDVPGRALEQVIIARLPFKVPTEPVLEARSEAIEKRGGD PFMEYTVPQAVIKFKQGFGRLIRHRDDRGVVMILDARVVKKGYGRVFLRSLPEARVVAAPSAEVLAEMRRFFAAPPPDAP
DNA	1	TTTTTTTTTTT
Ligand	1	ATP
Ion	1	Mg
Ion	2	Zn

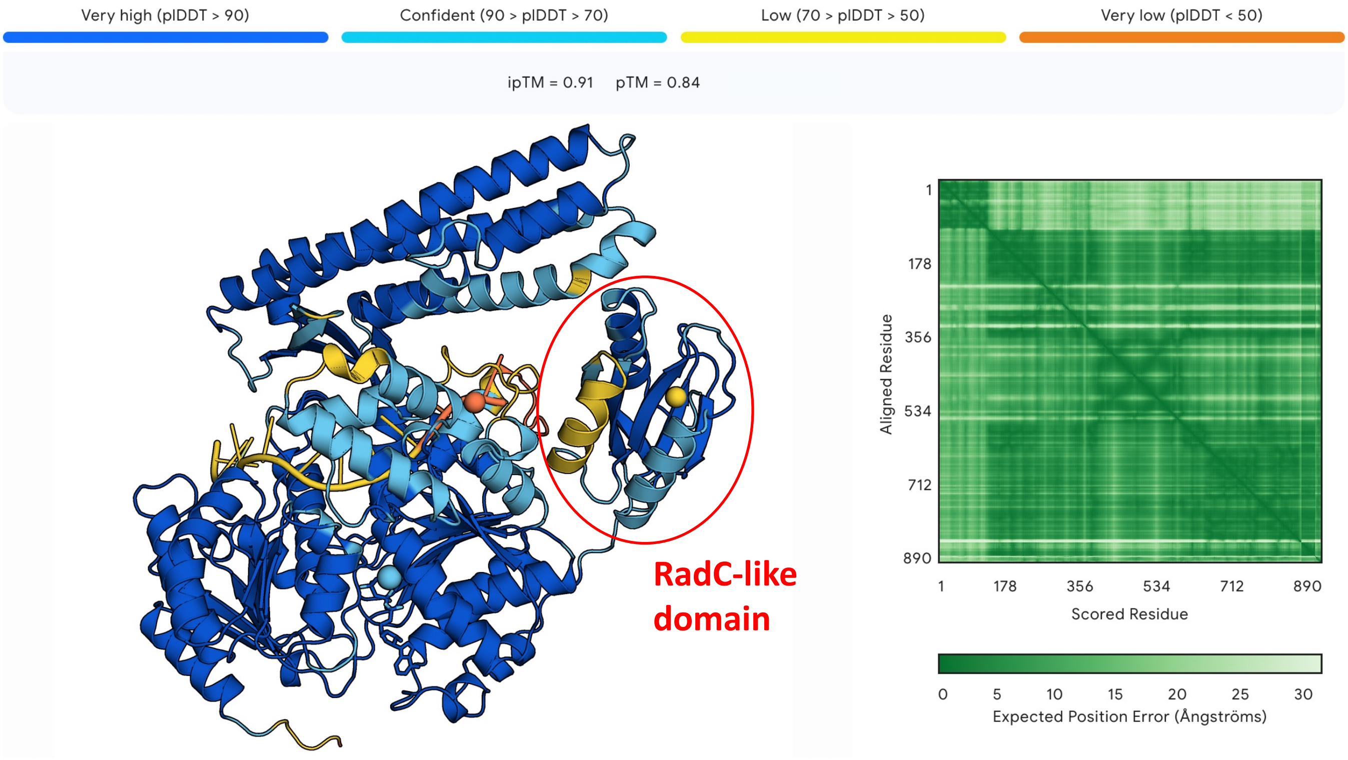

B

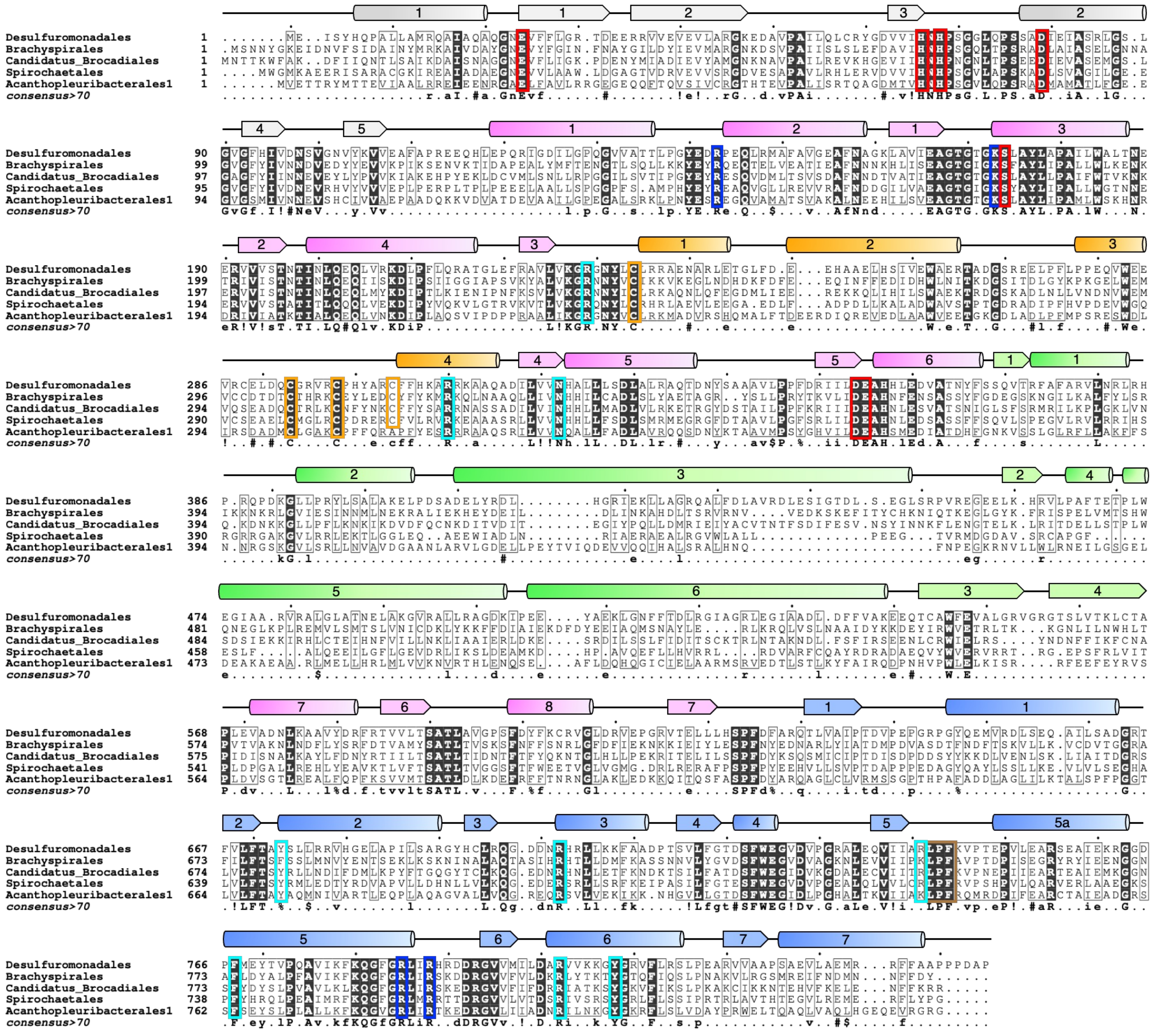
Desulfuromonadales
Brachyspirales
Candidatus_Brocadiales
Spirochaetales
Acanthopleuribacterales1
consensus>70
