# Supplementary material for: Structure, function and evolution of the bacterial DinG-like proteins
Source: Comput Struct Biotechnol J. 2025 Mar 17;27:1124–39. doi: 10.1016/j.csbj.2025.03.023 (PMC11981726; doi:10.1016/j.csbj.2025.03.023)
Supplement: Figure S9 — Supplementary material [file mmc9.pdf]

Figure S9 Supplemental information for sDinG subgroup proteins.

A. The structural model of the *Nostoc sp.* PCC 7120 sDinG–ssDNA complex in the presence of ATP·Mg^{2+} was predicted using AlphaFold 3. The input parameters, including protein sequences, substrate information, and ligand specifications, are detailed in the corresponding figure. The model′s quality assessment metrics are presented alongside the structural prediction. Despite setting DNA sequence and ATP·Mg as input, sDinG failed to incorporate high-quality models for DNA and ATP·Mg.

B. Multiple sequence alignment of sDinGs was performed using Clustal Omega and visualized by ESPript. The names of corresponding bacteria species, protein IDs, and protein sequences were provided in Table S1. Secondary structural elements were depicted based on the AlphaFold 3 predicted *Nostoc sp.* PCC 7120 sDinG–ssDNA complex structure, displayed at the top of the sequences, numbered, and colored according to domain arrangement. Critical residues for metal coordination, ATP binding, DNA binding, and the P motif were highlighted in red, blue, cyan and brown boxes, respectively.

A

Input	Copies	Sequence
Nostoc sp. PCC 7120 sDinG	1	MIEAEVHLSLHNFLRSQAGFPSWPHHLTMARLVARALRLGRSALIQVGAVCGYQGRYRTSFIASALMWHGPVIIVATETVQQLLLRVEIPRLQQWLQVNKPIRTGDAWPNPEFQGILLTSPEAWLRGQLTSADNFPQGIPTIIDGVDDLEDWVRHQLTQDIQPQDWDQLILACPEQAETIRLARIELTSELFKHPANPYECYLISPSETDILTKLHTALKSASGVPEVWQKFWQQLPSEQNHPPSSSPPLFWATIARRQGLFSLHYAPIELGDILSPIWQRQPVVLIGSALEPETEAPLFQERLGLDDLTCLKFASDSQSEAIQLYIPYKLPLPNTPEFQAAFIHKVRTLVCLSATAPGLTVLLVGDVPLKAQVGAILASEFGSRVQVEKTCLDENGILVSGWEFWREHQAVLPAPQLLVIATLPLPSLEHPLVAGRVAHYKRSHQDWFRLFLLPTALNELQRAVAPVRENQGIVALLDSRVVNRSYGSQILNVLSPLARLNYLDPSLFAPSGEENSA
DNA	1	TTTTTTTTTTT
Ligand	1	ATP
Ion	1	Mg

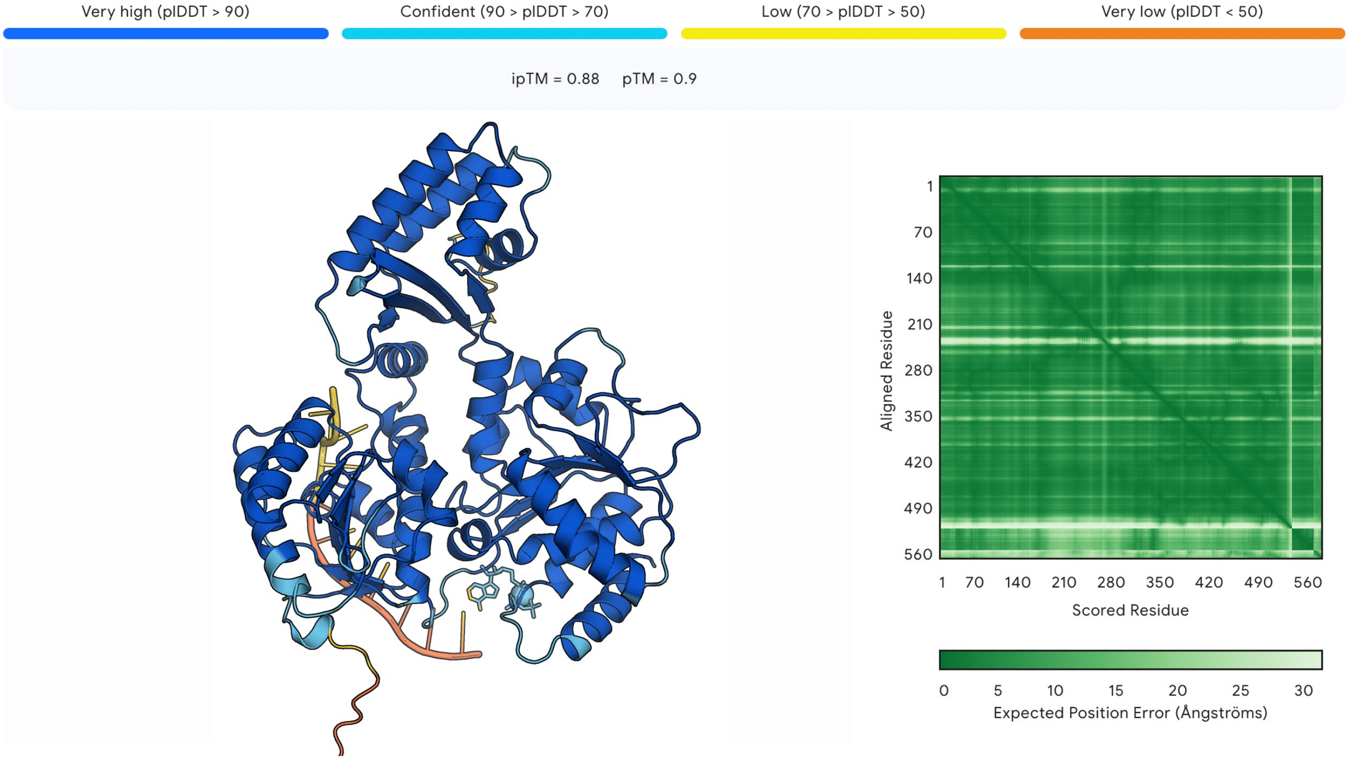

B

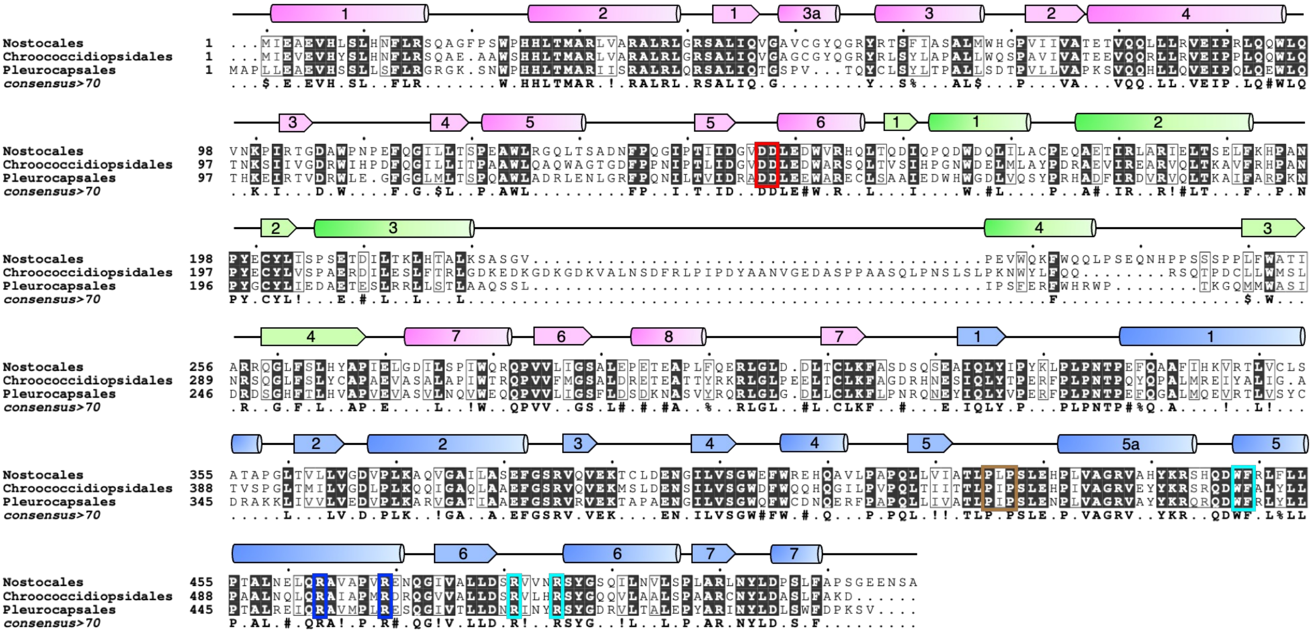
Nostocales
Chroococcidiopsidales
Pleurocapsales
consensus>70
